# Progress of Photocapacitors

**DOI:** 10.1021/acs.chemrev.2c00773

**Published:** 2023-06-09

**Authors:** Natalie Flores-Diaz, Francesca De Rossi, Aparajita Das, Melepurath Deepa, Francesca Brunetti, Marina Freitag

**Affiliations:** † School of Natural and Environmental Science, Bedson Building, Newcastle University, NE1 7RU Newcastle upon Tyne, United Kingdom; ‡ CHOSE (Centre for Hybrid and Organic Solar Energy), Department of Electronic Engineering, University of Rome “Tor Vergata”, via del Politecnico 1, 00133 Rome, Italy; ¶ Department of Chemistry, Indian Institute of Technology Hyderabad, Kandi, 502285 Sangareddy, Telangana, India

## Abstract

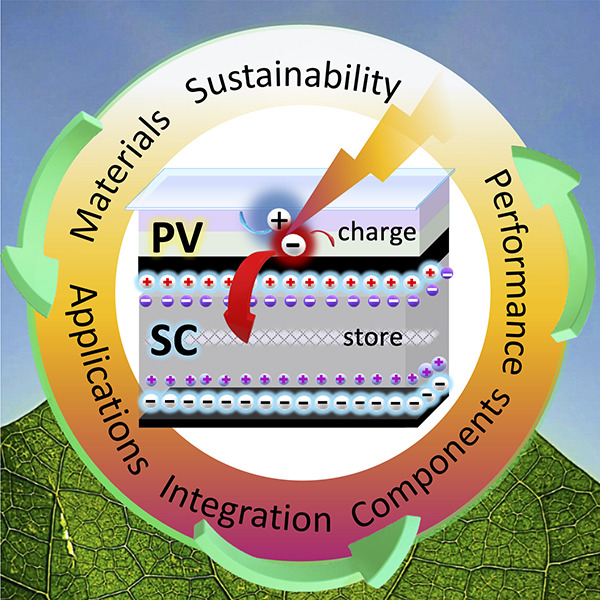

In response to the current trend of miniaturization of electronic devices and sensors, the complementary coupling of high-efficiency energy conversion and low-loss energy storage technologies has given rise to the development of photocapacitors (PCs), which combine energy conversion and storage in a single device. Photovoltaic systems integrated with supercapacitors offer unique light conversion and storage capabilities, resulting in improved overall efficiency over the past decade. Consequently, researchers have explored a wide range of device combinations, materials, and characterization techniques. This review provides a comprehensive overview of photocapacitors, including their configurations, operating mechanisms, manufacturing techniques, and materials, with a focus on emerging applications in small wireless devices, Internet of Things (IoT), and Internet of Everything (IoE). Furthermore, we highlight the importance of cutting-edge materials such as metal–organic frameworks (MOFs) and organic materials for supercapacitors, as well as novel materials in photovoltaics, in advancing PCs for a carbon-free, sustainable society. We also evaluate the potential development, prospects, and application scenarios of this emerging area of research.

## 1 Introduction

The urgent need to transition from fossil fuels to renewable energy sources has spurred the development of cutting-edge energy conversion and storage technologies.^1−12^ Photovoltaic (PV) systems have emerged as a leading solution to address the growing demand for carbon-free energy, with applications in various technical domains, such as photoelectrochemical water splitting (PEC),^13−16^ photocatalysis,^17−22^ and photoelectrochemical redox flow batteries.^23−26^ However, the inherent variability and unpredictability of solar radiation pose significant challenges to the widespread deployment of solar power, necessitating high-efficiency energy conversion and low-loss energy storage technologies.

Photocapacitors (PCs) offer an innovative energy conversion and storage technology by combining a photovoltaic or energy harvesting unit with a supercapacitor (SC) or an energy storage unit. This dual-use system allows for efficient generation and storage of power in a single device, making it suitable for a wide range of applications.^27,28^ PCs are based on the central concept of a self-charging capacitor that can directly store the electrical energy produced by photovoltaic cells, as proposed by Miyasaka and Murakami.^29−32^ This integrated system allows for efficient generation and storage of power, making PCs suitable for a wide range of applications in next-generation electronic devices and systems, particularly as energy demands increase and the need for sustainable, self-sufficient power sources becomes more critical.^33−37^

A photocapacitor comprises a photovoltaic or energy harvesting unit coupled to a supercapacitor (SC) or energy storage unit.^32,38−41^ The energy from a light source (solar or artificial) is transformed into electrical energy by the PV unit.^42−47^ The photogenerated charges are channeled into the SC unit, where they are stored at the electrodes of the supercapacitor.^48−52^ The components of the PV unit vary depending on the technology. First- and second-generation solar cells can be adapted to photocapacitors but on limited architectures.^9,53−57^ Third-generation photovoltaic technologies, including organic photovoltaics (OPVs),^58−61^ perovskite solar cells (PSCs),^62−65^ dye-sensitized solar cells (DSCs),^10,66−71^ and quantum-dot solar cells (QDSCs),^72−75^ are preferred for developing PCs due to their ease of fabrication, compatibility with various architectures, and cost-effectiveness. These technologies consist of a photoactive material or working electrode (WE), a redox electrolyte or hole transport material (HTM), and a counter electrode (CE). The supercapacitor unit is composed of two electrodes containing electroactive materials capable of storing charge as an electric double layer (EDL), a membrane separator, and an ion-conducting electrolyte.^76−79^

While the highest reported charge storage efficiency of an integrated photocapacitor is approximately 20%,^28^ further improvements in the intrinsic properties of the active materials, interface quality, and device integration are needed to enhance overall efficiency and commercial viability. Factors that influence a PC’s overall photoconversion and storage efficiency include the bandgaps of various semiconductors, hole–electron recombination, and the quality of multiple interfaces. Optimizing these factors is crucial for boosting the efficiency of PCs beyond 25% and enabling their commercial availability.^32^

Photocapacitors present an elegant solution for the development of self-powered electronic devices in various applications, including the Internet of Things (IoT) and the Internet of everything (IoE). The IoT encompasses sensors, actuators, wireless communication networks, and data processing, leading to energy regulation and the creation of intelligent buildings.^80−85^ The widespread deployment of IoT devices in agriculture, health, and business will significantly benefit society by enhancing energy efficiency.^86−89^ Typically, these electronic devices require an energy source, such as batteries, supercapacitors, or separate photovoltaic and energy storage units.^90^ PCs represent a compact and efficient alternative to these independent energy sources, enabling the development of self-powered devices and systems.^91−94^

**Figure 1:**
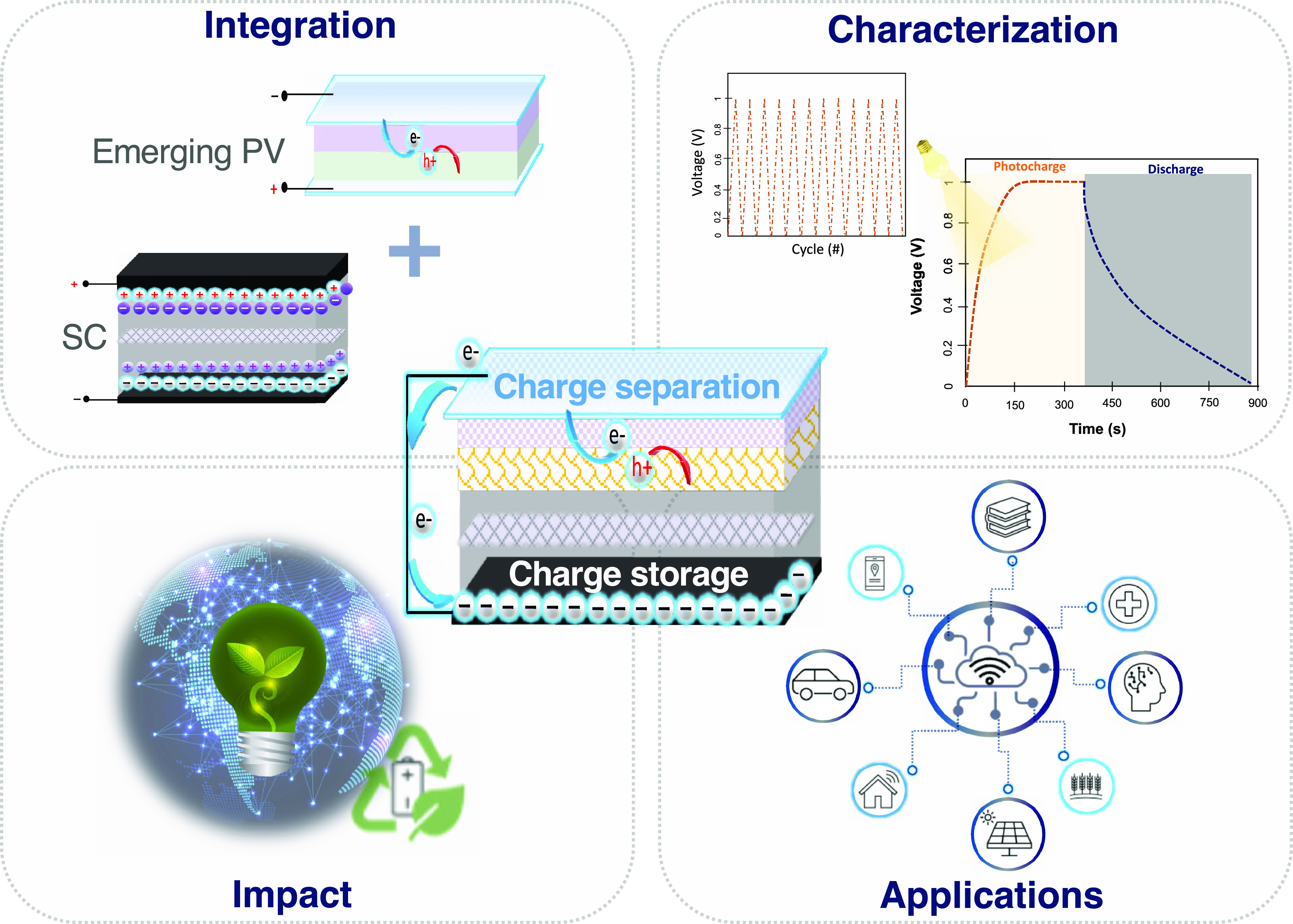
Schematic illustrating the integration of photocapacitors from individual components into a singular device capable of light harvesting and charge storage. Multiple applications are enabled by the use of photocapacitors, and their development will lead to more efficient and sustainable energy consumption.

The adoption of PCs also circumvents the need for two physically separate devices for energy conversion and storage, reducing space, cost, and weight while boosting the overall package efficiency. As illustrated in Figure 1, PCs can be designed as compact devices that facilitate the paradigm shift in the electronic era. The potential applications of PCs expand further when the concept of IoT is extended to the Internet of Everything (IoE), which encompasses intelligent connections among people, electronic gadgets, and data. Widespread adoption of photocapacitors will result in significant societal benefits, including energy savings, increased use of renewable energy, self-powered artificial intelligence, enhanced connectivity, and data transfer.

Moreover, the production of photocapacitors can be entirely adapted to a circular economy, making them an excellent sustainable alternative to current storage technologies. Their eco-friendly nature aligns with global efforts to mitigate climate change and promote sustainable development.

This review provides a comprehensive overview of photocapacitors by examining three aspects: photoelectrode and capacitive materials, PC characteristics, and photoelectronic device systems. First, we summarize the research status in materials, components, and device engineering of photocapacitors, including photovoltaic characteristics and supercapacitor materials. We also discuss the bandgaps of various semiconductors, hole–electron recombination, and factors that influence the overall efficiency of PCs. Second, we review known methodologies to determine and enhance the performance of photocapacitors. These approaches include optimizing the intrinsic properties of the active materials, improving interface quality, and refining device integration. We also highlight potential strategies for developing highly efficient light conversion integrated systems, paving the way for further advancements in photocapacitor technology. Lastly, we explore the future applications of photocapacitors in sustainability and their potential impact on various industries. We assess the prospects of PCs in IoT and IoE systems, intelligent buildings, agriculture, health, and business. We also discuss the role of PCs in promoting energy efficiency, self-powered artificial intelligence, enhanced connectivity, and data transfer, ultimately contributing to a more sustainable future.

In summary, photocapacitors represent a promising avenue for the development of efficient energy conversion and storage technologies. By providing a comprehensive overview of photocapacitor materials, characteristics, and systems, this review aims to inspire further research and development in this emerging field. The ongoing advancements in photocapacitor technology will not only contribute to the global transition toward renewable energy sources but also enable the realization of innovative applications in various sectors, paving the way for a sustainable future.

## 2 Applications of Photocapacitors

### 2.1 Indoor Applications

Lithium-ion batteries (LIBs) are the most widely used energy storage technology due to their high energy density. Despite this, they present several disadvantages, including relatively high prices, inadequate safety precautions, and a limited supply of lithium and cobalt, whose mining is frequently associated with exploitative working conditions and a significant environmental impact.^95^ Moreover, they cannot endure numerous charge/discharge cycles, limiting their lifespan and leading to replacement regularly, resulting in billions of hard-to-recycle batteries per year.

Employing an electrical double layer (EDL) or a supercapacitor (SC) in locations that demand fast, powerful, and secure energy storage devices can overcome these problems.^96^ Due to their lower energy density than batteries, supercapacitors can be charged entirely or discharged in seconds or minutes. However, larger power output can be achieved for brief periods.

Supercapacitors can be charged sustainably by coupling them with low-cost, solution-processed photovoltaic cells. The resulting device, a photocapacitor (PC), will pave the way for the development of self-sufficient gadgets that function as an independent power source, requiring nothing more than solar or indoor light and thus playing an essential role in the transition to renewable energy sources for a wide range of applications.^97−102^

The photovoltaic unit of the incorporated device rapidly charges the supercapacitor unit upon illumination by any available indoor light or solar irradiance (when outdoors). Small electronic devices, such as the digital display of a pregnancy test kit, biosensors, wearable electronics, and Bluetooth-enabled devices, can be powered by the energy produced and stored in the photocapacitor. Some other applications include power grids, emergency door sensors in transport media, pulse power in communication devices, large-scale energy supply systems, and photochromic applications.^103−113^ As a result, PCs can potentially replace batteries with the added benefit of substantially higher stability than standard coin cells or LIBs.

**Figure 2:**
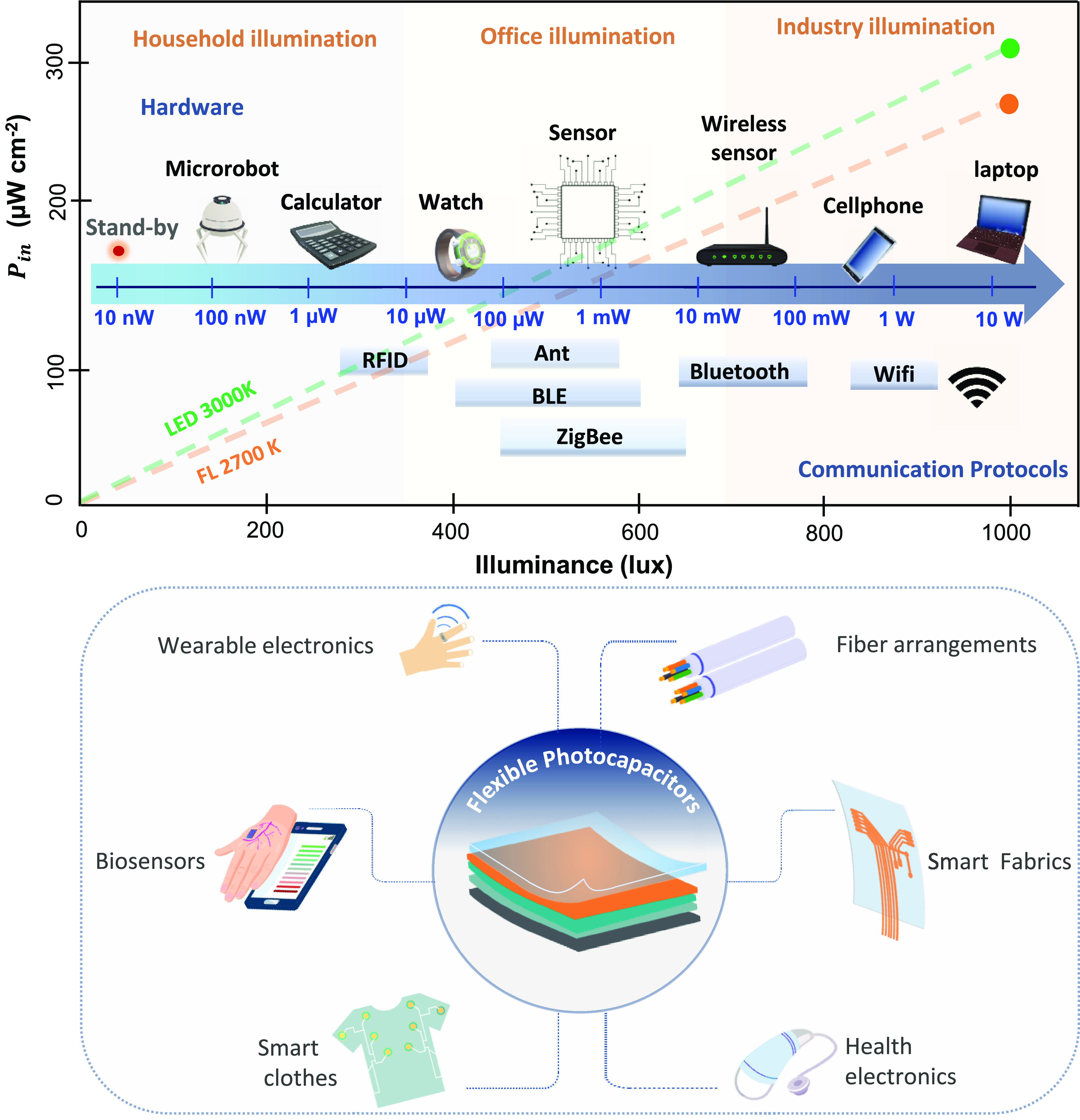
Schematic representation of the illuminance levels in different settings. As depicted in the diagram, most sensors and communications protocols require power from 10 μW to 100 mW. Efficient PCs with enough active areas can meet the power requirements of each application. The design of flexible photocapacitors broadens their ambient light applications and wearable electronics.

The Internet of Things devices and ecosystems need to be able to sense, process data, and communicate with each other.^114,115^ Wireless machine learning and artificial intelligence are achieved by smart sensing and local data processing. These can be performed with low-power microprocessors and microcontrollers, determining the power requirements of the IoT devices.^80,81^ For instance, the power consumption of passive and active radio frequency identification (RFID) tags, as well as a wireless communication protocol that ensures data connectivity ranges from μW to W (Figure 2).^102^ IoT devices with limited sizes ranging from 1 mm to 10 cm can be embedded on various substrates (glass, flexible substrates, clothing, fibers, etc.).^116,117^ As a result, their energy supply systems must be lightweight, adaptable, and miniaturized.

The light intensity attained by ambient light sources ranges from 50 to 300 lx in residential settings, 300–500 lx in offices, and 1000 lx in industrial environments, much lower compared to the illumination levels at 1 sun illumination of 100–110 000x (at AM1.5G, 1000 W m^–2^).^118^ The emission spectra vary between each lamp source. CFL Fluorescent and LED-based lights emit mainly in the visible region, which provides an excellent opportunity to develop photocapacitors tuned to harvest the light from their surroundings efficiently.

Modern LED sources offer efficacies up to 100 lm/W, with an irradiance around 300 μW cm^–2^ at 1000 lx conditions.^119^ Employing a highly efficient PV unit designed for indoor-light harvesting with a PCE of around 35% will readily deliver 105 μW cm^–2^.^120^ Conventional sensors require from 100 μW to 1 mW, depending on the operation mode.^121^ Ambient light sources from 100 to 1000 lx can provide from 25–300 μW cm^–2^, sufficient to power IoT devices.^98^ The Shockley–Queisser efficiency limit for indoor photovoltaics under 500 lx from a white-LED source is approximately 52%.^93^ Further advances in the following years will bring ambient photovoltaics closer to 50% PCE, allowing them to produce approximately 150 μW cm^–2^.

As indicated in Figure 2, the power consumption of electronic devices and communication systems can range from low power in the range of μW to high power depending on the application (hundreds of W). The purpose of photocapacitors in this context is to replace traditional energy supply systems with specialized power supplies for each application.

Many architectures have been used on rigid and flexible substrates to fabricate photocapacitors. The hybrid four-terminal (4-T) architecture comprises the supercapacitor and solar cell individually constructed and then connected via wiring (Figure 4). The key challenges with a hybrid design are the need for external wiring and solid-state electronic components that are undesirable for integrated applications. In contrast, the integrated 3-T and 2-T architectures comprise a single device in which the energy harvester and storage unit share two or more terminals (see Figure 4). Due to the compactness of the completed device, these architectures will be the focus of future research as they overcome the need for external wiring. To increase the overall efficacy of the photocapacitor device, however, the direct coupling or stacking of materials requires additional engineering to reduce charge losses between the interface of the PV and SC units. In addition, further research must address the need for a diode material between the photovoltaic and supercapacitor components to prevent charge recombination from the supercapacitor to the PV. Generally, using a diode will result in a significant voltage drop, and this issue must be addressed by developing novel materials with diode-like behavior.

Operating photocapacitors in high-power applications offers a more compact and dependable alternative to large-area and bulky battery systems. The PCs can be coupled to solar modules and used as a fast-response energy storage unit alongside battery units to increase the total power supply.^122^

### 2.2 Flexible Photocapacitors

As previously stated, the critical focus for numerous applications will be the construction of high-performance flexible photocapacitors. The PV community has prioritized the development of flexible photovoltaic cells in recent years, opening the path for flexible PCs.^123−127^ Flexible substrates have been widely investigated for photocapacitors as they enable applications where flexibility and lightweight are essential requirements. Despite current flexible integrated devices exhibiting lower total energy conversion and storage efficiency than their rigid counterparts, innovative architectures have been proposed and efforts made toward more efficient and stable self-powered devices.

The manufacturing of flexible devices typically involves roll-to-roll or slot-die coating techniques, which can be complemented by spray processing, inkjet printing, and 3D-printing techniques.^128^ Additionally, 3D printing can provide materials with enhanced surface area/charge storage properties and can be performed conveniently on top of the PV unit.^129^ For instance, porous carbon materials for energy storage applications have been successfully 3D-printed,^130^ highlighting the potential of this technique to integrate capacitive materials on top of the photovoltaic unit in a monolithic PC arrangement (2T or 3T) that benefits from compact packaging and requires fewer materials. In addition to producing membranes with custom-sized pores, 3D printing processes can be employed to deposit the solid electrolyte. The critical challenge is developing adequate inks/slurries of active materials, particularly those with rheological specifications that allow for high-quality printing.^131^

Moreover, employing conductive polymers for the SC unit and as components of the PV unit can facilitate the production of flexible photocapacitors.^115,132^ Typically, flexible devices employ polymer substrates such as polyethylene terephthalate (PET), polyethylene naphthalate (PEN),^133^ and polyimide (PI),^134^ with a thin layer of a transparent conductive oxide, usually ITO; however, fabric materials and different paper materials have also been investigated.^135,136^ Low-temperature techniques for the annealing of the components of the PV unit represent the bottleneck for high-performing flexible photovoltaics compared to their rigid substrate counterparts.^123,124,137−139^ For instance, the commonly employed semiconductor TiO_2_ requires high annealing temperatures, which are incompatible with flexible substrates.^125^ Alternative processes have been developed to meet the need for low-temperature annealing. One possible alternative is using Flash Infrared Annealing (FIRA), a cost-effective technique easily adapted to scale production lines.^140^

Flexible photocapacitors for IoT applications have been developed by coupling commercial a-Si and GaAs and next-generation solar cells (DSC, PSC, and OSC) to flexible supercapacitors. Fu et al. introduced a flexible fiber-PC to power an LED in 2013.^35^ They employed anodic deposition to coat a stainless steel wire with polyaniline. This fiber electrode served as an electrode for the DSC photovoltaic unit and the SC part. The integrated power supply presented an overall energy conversion efficiency of up to 2.1%. A series of four flexible fiber DSC and four flexible supercapacitors were connected through a hybrid approach. The integrated system was charged under 1 sun illumination and reached 2.34 V in 50 s.

A free-standing, highly conductive PEDOT:PSS film was developed as an effective alternative to metal-coated substrates as the common electrode for a flexible large area P3HT-ICBA OSC and a symmetric supercapacitor with a PVA-H_3_PO_4_ electrolyte. The 1 cm^2^ solar cell with the laminated PEDOT:PSS electrode delivered 3.84% PCE, the supercapacitor showed 58% energy storage efficiency, and the overall efficiency of the integrated photocapacitor was 2%. By adding an insulating layer on top of the nonshared PEDOT:PSS electrode of the supercapacitor, the energy storage unit could be folded on top of the solar cell, further reducing the metal dimensions of flexible photocapacitors.^141^

Moreover, flexible photocapacitors can facilitate the creation of a vast array of self-powered portable devices, including wristbands, wearable accessories, and skin sensors (Figure 2).^142^ Z. Wen et al. demonstrated a fiber-shaped PC with a DSC as the PV unit.^105^ The resulting textile can easily be woven into smart clothes. The system was connected through a 4-T approach.

Furthermore, a blocking diode was introduced to avoid undesired current discharge to the DSC unit. Three switches were used to control the charging process of the system; when the solar cell charges the series of supercapacitors, a voltage of 1.8 V is reached in 69 s.^105^

Another example of fiber-shaped integration of a flexible DSC and a microsupercapacitor has been reported by W. Song et al.^107^ Their system comprised three fiber DSCs connected in series between them and then connected to a series of microsupercapacitors through external wiring. The photocharging of the microsupercapacitor to 1.8 V was achieved in 30 s. Switches allow electricity to flow from the solar cell to the supercapacitor and then to the load, ensuring proper charging and discharging. The flexible energy supply system was used to power an electronic watch.^107^

Chen et al. coaxially integrated a DSC and a supercapacitor into a fiber-shaped photocapacitor: a titanium wire surface, modified with perpendicularly aligned TiO_2_ NTs and horizontally aligned CNT sheets acted as the electrodes for the energy conversion and storage units, completed by gel electrolytes. The DSC and SC showed an efficiency of 2.73% and 75.7%, respectively, with a maximum of 1.2% for the overall efficiency.^143^

Recently, Liu et al. presented a novel design consisting of a self-supported graphene/CNT hollow fiber: PANI was deposited on both the inner and outer surfaces of the fiber with high mass loading. Such structure enabled a supercapacitor with a large specific capacitance of 472 mF cm^–2^, which replaced the platinum wire in the fiber-shaped DSC that achieved 4.2% PCE; the integrated photocapacitor exhibited an overall efficiency of 2.1%.^144^

A similar approach to designing wearable electronics has been reported by C. Li et al.^113^ They demonstrated the versatility of flexible photocapacitors with a self-powered wearable gadget that monitors physiological signs when in contact with the skin. In their study, a series of flexible perovskite solar cells were integrated through wiring with a lithium-ion capacitor and connected to a strain sensor that monitors the pulse signal and finger motion. The integrated system delivers an overall efficiency of 8.41% with a high output voltage of 3 V at a discharge current density of 0.1 A g^−1^.

W. Jin et al. constructed flexible photocapacitors employing OSCs as the PV units connected to flexible supercapacitors. The materials were printed on different substrates with metal-embedded transparent conductive electrodes and then stacked vertically to form an integrated device.^103^ Under 1 sun illumination, the device charges with an effective photocharge rate of 1.86 mV s^–1^ (photocurrent output 3.8 mA). The SC unit discharged with an effective galvanostatic rate of 56 A s^–1^, leading to an overall efficiency of 5.02%. Since these performances were insufficient to power electronic devices effectively, a module consisting of (2 series x 4 parallel connections) was fabricated through nickel wiring and silver paste. The module demonstrated a charging rate under 1 sun illumination of 1.03 mV s^–1^ (photocurrent output 7.8 μA), with an overall efficiency of 4.94%.^103^

Gao et al. combined a high-performance, cotton-textile asymmetric supercapacitor with a flexible solar module to build a self-charging power pack using scalable roll-to-roll manufacturing.^104^ The integrated device was capable of delivering an open circuit voltage of 3 V under illumination and was able to power the LED for 10 min after the light was switched off.^104^

Recently, Liu et al. reported an efficient ultrathin flexible photocapacitor with an overall efficiency approaching 6% and a total thickness below 50 μm: a 3 μm thick OSC was integrated on top of a 40 μm CNT/polymer-based supercapacitor on a 1 μm PET substrate. Besides the high performance, the integrated device also showed high operational stability, retaining over 96% of its initial efficiency after 100 charge/discharge cycles and mechanical robustness, losing only 5% of its initial efficiency after 5000 times bending at a radius of around 2 mm.^145^

## 3 Integration of Photocapacitors

Numerous advantages are associated with using of photocapacitors as sustainable and compact solutions for self-powered devices, as evidenced by their multiple applications. Several research fields are required to design and manufacture the various components (PVs and SCs) of the dual-system, resulting in a more efficient and stable device. This section will discuss the operating principles of photovoltaics, supercapacitors, and other components.

### 3.1 Emerging Photovoltaics

**Figure 3:**
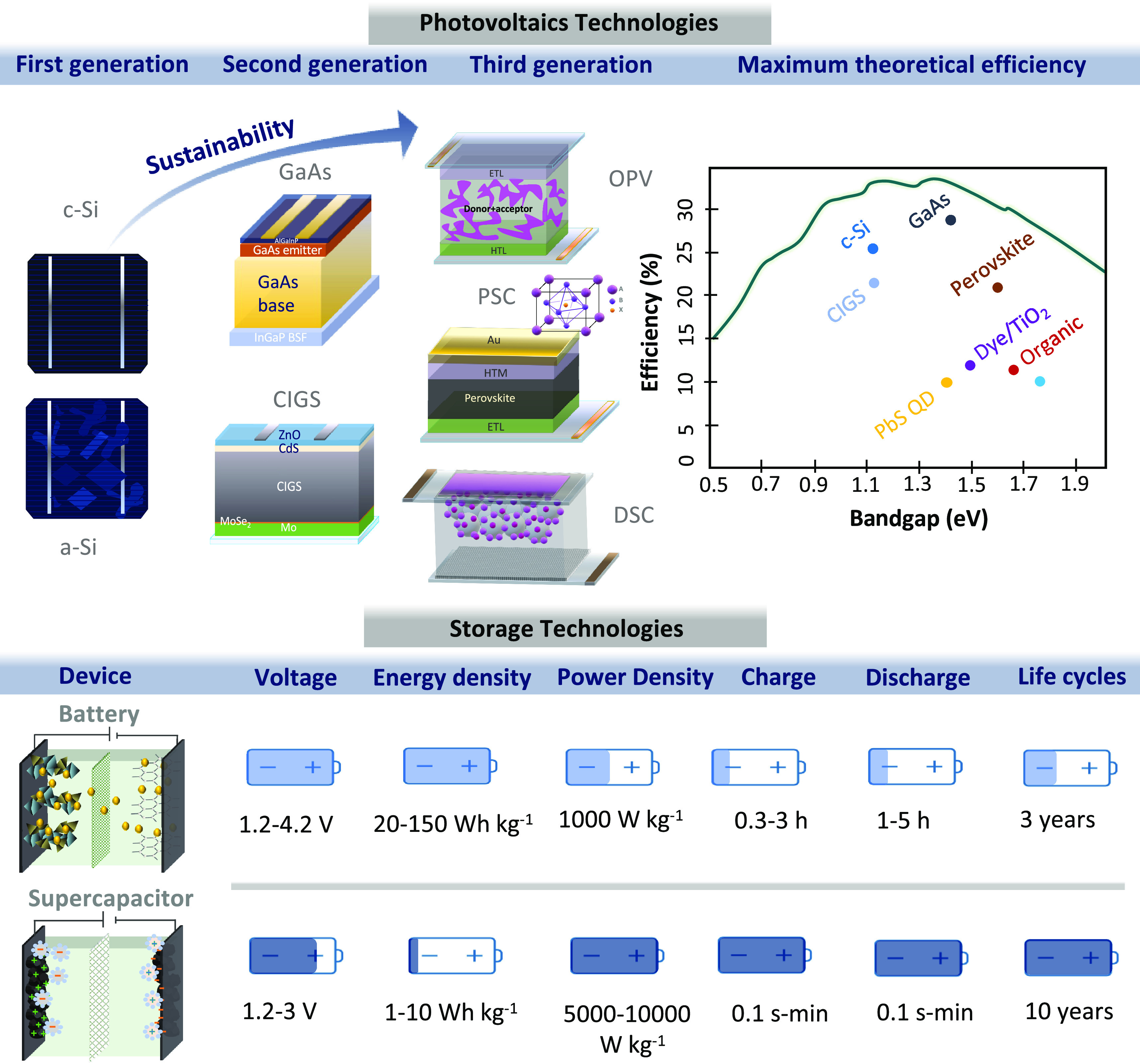
Top left: Different photovoltaic technologies. First-generation solar cells based on c-Si and a-Si, second-generation solar cells: GaAs and CIGS, among others, and third-generation or emerging solar cells: OPVs, PSC, and DSC or QDSC with similar structures. Top right: Shockley–Queisser limit for photovoltaic technologies. Bottom: comparison of the performance of batteries vs supercapacitors.

Solar or photovoltaic cell technologies can convert light directly into electricity due to the photovoltaic effect. When a semiconductor material is exposed to light, the absorption of photons with energy equal to or greater than the bandgap (*E*_g_) of the material leads to the excitation of an electron or other charge carriers to a higher-energy state. The photogenerated electron–hole pairs are separated, the electrons are extracted as current by an external circuit, and the holes are quenched at the positive terminal. Silicon-based technologies comprise the first generation of photovoltaic technologies (PVs), with crystalline silicon (c-Si) with a bandgap of *E*_g_ 1.1 eV showing the highest power conversion efficiency (PCE) of 26.7% under 1 sun illumination.^146^ Amorphous silicon (a-Si:H) with an *E*_g_ of 1.6 eV^147^ is frequently used despite the lower PCE of 11.9% due to it is lower cost compared to c-Si.^146^ Under low light conditions of 1000 lx, a-Si:H can deliver up to 21% PCE.^147^ Second generation photovoltaics include GaAs, CdTe, and CIGS, among others. Unfortunately, these materials are costly, require limited resources, and are difficult to integrate into photocapacitors.^148^ Third generation or emerging technologies (Figure 3), such as organic photovoltaics (OPVs), perovskite cells (PSCs), and dye-sensitized cells (DSCs), can be manufactured at lower cost, with Earth-abundant materials and solution-processed techniques, easily adapted to industrial production. As a result, they are the favored photovoltaic technology for producing photocapacitors. Table 1 summarizes the photovoltaic parameters of the most common PV technologies.^149^

**Table 1:** Comparison of Photovoltaic Parameters of Different PV Technologies under the Global AM1.5 Spectrum (1000 W m^–2^) at (ASTM G-173-03 Global or IEC 60904-3:2008)

Technology	Area (cm^2^)	*V*_OC_ (V)	*J*_SC_(mA cm^–2^)	Fill factor (%)	PCE (%)	Ref
c-Si	79	0.738	42.65	84.9	26.7	(146)
a-Si	1.001	0.896	16.36	69.8	10.2	(177)
GaAs (thin film)	0.998	1.1272	29.78	86.7	29.1	(178)
CIGS	1.043	0.734	39.58	80.4	23.35	(179)
OPV	0.0473	0.871	26.75	79	18.43	(180)
PSC	0.09597	1.179	25.8	84.6	25.7	(181)
DSC	0.1155	1.0648	18.049	78.97	15.178	(167)

Organic solar cells (OSCs) rely on carbon-based semiconductors, such as conjugated polymers, for their active layers. Organic semiconductor films generate strongly bound excitons with limited diffusion length upon light irradiation. OSCs comprise an electron donor, typically benzodithiophene and difluorobenzothiadiazole-based organic semiconductors, with fullerene-based electron acceptor semiconductors. Planar architectures can be achieved by successfully depositing the donor and acceptor, forming a heterojunction.^150^ In contrast, bulk heterojunctions are formed when the donor–acceptor molecules are mixed, reducing the path the exciton has to travel within its lifetime to be effectively separated.^151^ Due to their availability, nontoxicity, and low-temperature, low-cost manufacturing, OSCs have attracted significant interest. They can be printed on large areas and flexible substrates.^152−155^ Continuous efforts in the research and development of materials have pushed the single junction efficiencies to values as high as 18.2% under 1 sun illumination,^53^ and 28.8% under 1000 lx illumination from a 2600 K LED lamp.^156^

Perovskite solar cells (PSCs) have attracted significant interest from the academic and industrial sectors due to their ease of fabrication based on solution processing. They have reached remarkable power conversion efficiencies of over 25% in single junction and above 31% in tandem with silicon solar cells.^53^ Organic halide perovskites such as CH_3_NH_3_PbI_3_, which act as the light absorber, present unique advantages such as high extinction coefficient, bandgap tunability with perovskite composition, low carrier recombination rate, and carrier diffusion length on a micron scale.^157−159^ PSCs are composed of an electron transport layer (ETL), a hole transport material (HTM), and metal contacts. The ETL is the light-harvesting layer that generates charge carriers upon light absorption. The electron–hole pairs split, and the HTM scavenges the hole carriers. Different architectures such as mesoscopic, planar, and inverted, have been investigated in the past decade. Perovskite photovoltaics can reach PCEs above 40% under 1000 lx under indoor illumination.^160^ Furthermore, PSC technology has been demonstrated to be scalable to large-area modules, adopting deposition techniques compatible with industrial manufacturing,^161−163^ and compatible with flexible substrates.^65^

Dye-sensitized solar cells (DSCs) were the first solar cells among third-generation PV to be considered for integration with energy storage devices in photocapacitors.^29,38,164^ Due to their simple design, abundant, nontoxic materials, and relatively easy manufacturing process,^165^ they result in low production costs, variety in the choice of substrates (either rigid or flexible, e.g., lightweight plastic or metal foils, wires or fibers) and active materials with tunable optical properties, such as color and transparency.^10,166^ DSCs comprise a wide bandgap semiconductor such as TiO_2_ with dye molecules (sensitizers) adsorbed onto its surface, a counter electrode (CE) and a redox mediator between the electrodes. The dye molecule generates photoinduced electrons upon irradiation, which are injected into the TiO_2_ conduction band. The redox shuttle facilitates dye regeneration and the transfer of positive charges (holes) from the WE to the CE. Advanced molecular systems, including panchromatic rigid-structure dyes, alternative hole transport materials (HTMs), and design flexibility, have increased power conversion efficiencies (PCEs) up to 15% under AM1.5G conditions,^167^ and remarkably to a record 34.5% under 1000 lx ambient light illumination.^6^ The superior performance of DSCs under low light^6,168−171^ make them a suitable technology for indoor light harvesting and, ultimately, a technology with great potential for the development of highly efficient photocapacitors that harvest indoor light to power electronic devices located in ambient settings.

Quantum dot solar cells (QDSCs) employ nanocrystalline semiconductor quantum dots (QD) as sensitizers or photoactive material.^172−175^ QDs feature size-tunable bandgap and size-dependent behavior, absorbing light over a broad range of wavelengths and generating multiple excitons for each photon absorbed. They can be synthesized at relatively low temperatures and then processed by solution techniques,^176^ and have achieved record efficiencies as high as 18.1% employing Pb- and Cd-free-based QD sensitizers.^53^

### 3.2 Supercapacitors

The leading energy storage technologies used in a wide range of applications include batteries, conventional capacitors, and supercapacitors (SC), also known as ultracapacitors.^182^ Some of the applications employing these technologies as power supply include (but are not limited to) consumer electronic devices, household gadgets, power generation, computation, wireless devices and chargers, electric vehicles, stationary grid storage, industrial systems, medical sensors, among others. The primary difference between a battery and a supercapacitor is their capabilities: batteries offer a higher energy density (implying that they can store more energy per unit weight, typically a few hundred Wh kg^–1^). Supercapacitors offer a higher power density (they can release more power over a short duration of time, usually tens of thousands of W kg^–1^),^182^ as shown in Figure 3.

Supercapacitors store and release charge via electrical double layer (EDL) formation.^183^ During the charging process, the Helmholtz compact layer is formed at the electrode’s interface accumulating opposing charges from the ionic electrolyte (Figure 5), followed by a diffuse layer of charges in the immediate environment of the Helmholtz layer (hence double layer). A pseudocapacitive behavior is observed when there is faradaic charge accumulation from redox reactions at the electrolyte-electrode interface.^184^ Due to their fast charge/discharge rates, SCs are well-suited for applications such as regenerative braking-capturing and storing braking energy produced in trains, trucks, buses, and automobiles and then releasing it on a need basis. They are ideal for fast-charging backup power. One of the most significant advantages of supercapacitors over batteries is their capacity to provide short-term, high-power bursts.

In contrast, batteries store charge by diffusion-controlled faradaic oxidation and reduction.^185^ Ion diffusion over the active material layer slows charging and discharging to tens of minutes or hours, while SC may charge/discharge in seconds. This difference gives batteries a high energy density, making them appropriate for long-term power applications (e.g., mobile phones and laptops, among others).

The use of toxic or limited components such as cobalt and lithium can be avoided in SCs, which is one of the main concerns regarding the sustainability of lithium-ion batteries. In addition, SCs require minimal maintenance and have ultralong operational life as they can endure a million charge–discharge cycles without degradation. Furthermore, SCs can be safely operated over a vast temperature range of −40 to +60 °C without significant performance variation.^186,187^ When used in conjunction with batteries, supercapacitors complement and extend battery life at an affordable price, and under certain conditions, they can also act as an adequate battery replacement. One of these scenarios is when SCs are coupled to a PV unit to create photocapacitors that can store the charges produced by light harvesting and serve as a battery replacement.

In terms of material characterization, both batteries and supercapacitor materials can be tested in the classical three-electrode setup in an electrochemical cell with the active material as the working electrode, compared to a counter and reference electrode. They can also be tested in two-electrode systems, depositing the active materials in two electrodes (symmetrical supercapacitor) or studying different materials in each electrode (asymmetric supercapacitor).

Activated carbon (AC) is the predominant active material in commercial supercapacitors, with capacitance values ranging from 100 to 400 F g^–1^.^212^ Many alternative materials to activated carbon (AC), such as carbon nanotubes (CNTs), graphene (G), (reduced) graphene oxides (RGOs), transition metal oxides and chalcogenides such as RuO_2_, MnO_2_, and MoS_2_ offer higher capacitance. However, the synthetic ease, scale-up, and expense of these materials offer substantial barriers to their use.^213^ Carbon-based materials are usually characterized by a high effective surface area (a few thousand m^2^ g^–1^), resulting in high capacitance (few hundreds of F g^–1^) and high power (10 kW kg^–1^).^214−217^

Transition metal oxides such as RuO_2_, MnO_2_, NiO, and metal sulfides such as MoS_2_, Cu_2_S, among others, with nanostructured morphologies show high capacitance and energy density due to their pseudocapacitive behavior or their ability to reversible undergo oxidation and reduction processes (Table 2).^188,189,218−220^ These metal oxides or chalcogenides have redox active metal centers (e.g., Mo, Ni, Fe, V, Cu) with partially filled d orbitals, capable of undergoing reversible reduction or oxidation as shown in eq 1:
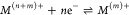
1

**Table 2:** Comparison of the Capacitive Performance of Different Materials

Positive electrode	Voltage (V)	Electrolyte	Capacitance (F g^–1^) (Current, A g^–1^)	Energy (Wh kg^–1^)	Power (kW kg^–1^)	Retention (%) (cycles)	Ref
NiO	0.4	KOH 1 M	1776 (1)	16.5	89	97.9 (1000)	(188)
CuS	0.3	KOH 6 M	833 (1)	–	–	75.4 (500)	(189)
PEDOT	0.8	H_2_SO_4_ 1 M	198 (0.5)	4.4	40.25	86 (12000)	(190)
MnO_2_	1.8	Na_2_SO_4_ 1 M	365 (0.25)	22.5	146.2	90.4 (3000)	(191)
MnO_2_/CNT	2	H_3_PO_4_/PVA	18 (0.267)	42	19.3	98 (500)	(192)
Mn_3_O_4_/G	1.8	KCl/PAAK	72.6 (0.5)	32.7	9.0	86 (10000)	(193)
RuO_2_/G	1.8	H_2_SO_4_/PVA	175 (0.5)	19.7	6.8	95 (2000)	(194)
PANI/CNT/G	1.6	H_2_SO_4_ 1 M	107 (1)	20.5	25	91 (5000)	(195)
MnO_2_/Ni foam	2.0	Na_2_SO_4_ 0.5M	41.7 (1)	23.2	1.0	83.4 (5000)	(196)
ZCOSH	0.43	KOH 2 M	116.3 (1)	41.4	0.80	92.4 (3000)	(197)
ZnO/AC	0.8	KOH 6 M	50.9 (2 mA cm^–2^)	4.52	1.62	–	(198)
PPy/GO/ZnO	0.9	KOH/PVA	123.8 (1)	–	–	92.7 (1000)	(199)
Ni(OH)_2_/AC/CNT	1.6	KOH 6 M	82.1 (0.5)	32.3	0.50	83.5 (1000)	(200)
NiO-CFP	1.8	KOH 2 M	240 (4)	105	12.7	68 (5000)	(201)
Fe_2_O_3_-CF	2	LiClO_4_/PVA	74	33.1	1.32	92 (10000)	(202)
Mn_2_O_3_/C	1.8	Na_2_SO_4_ 1 M	122 (2.5)	54.9	22.6	97 (5000)	(203)
NiCo_2_O_4_@GQD	1.6	KOH 2 M	107 (1)	38	0.8	71.8 (3000)	(204)
NiCo_2_O_4_@PPy	1.6	KOH/PVA	102.5 (8 mA cm^–2^)	58.8	10.2	82.9 (10000)	(205)
NiCoMn-S-1.5	1.6	KOH 6 M	111.6 (1)	36.3	0.75	93.9 (3000)	(206)
CuCo_2_O_4_/CuO	1.7	KOH 2 M	–	41.76	7.86	104.7 (5000)	(207)
NiSe-Se/Ni foam	1.6	KOH 1 M	84.1 (4 mA cm^–2^)	29.9	0.59	95.1 (10000)	(208)
Au@GGO-ZnCo_2_O_4_	1.4	KOH/PVA	113.8 (10 mA cm^–2^)	31	2.12	97 (5000)	(209)
NiB_*x*_O_*y*_	1.6	KOH (6 M)	–	42.4	0.8	97 (10000)	(210)
Co-Ni-S/CNT	1.6	KOH 6 M	178.6 (1)	63.5	0.8	83 (10000)	(211)

Conducting polymers (such as poly(3,4-ethylenedioxythiophene) (PEDOT), poly(aniline) (PANI), poly(pyrrole) (PPy)) are also capable of showing a pseudocapacitive behavior.^190,221,222^ By applying a voltage or current, they endure reversible doping by the electrolyte anions.^223^ Conducting polymers offer several benefits, including high electrical conductivity in the doped state, high chemical stability, and rapid charge–discharge kinetics. The swelling and shrinking of polymers over long charge/discharge cycles limit the system’s supercapacitance, making polymer-based supercapacitors challenging to commercialize.

Asymmetric supercapacitors use the same charge-storage mechanism with different active materials at the cathode and anode.^224^ In contrast, hybrid supercapacitors have distinct active materials with different charge-storage processes. They can have a different ratio of redox-active sites on the electrode material, separate redox-active electrolytes, or the same material with different surface functional groups.^225^ Combining a pseudocapacitive electrode with an EDL electrode provides a method for improving SC performance, as well as operating voltage and dark-discharge duration. The EDL electrode allows a high power capability, while the pseudocapacitor electrode provides a high energy density, thereby extending the device’s discharge time^226,227^

The electrolyte is essential for charge separation since it contains compensating anions and cations that diffuse to the electrode surface upon charging. The choice of the electrolyte and the ionic salts significantly influence the SC’s performance and considerably affects the operating window. Due to their strong ionic conductivity, low cost, and low toxicity, aqueous electrolytes are the most common choice for SCs. The thermodynamic breakdown of water limits the voltage window of aqueous-based electrolytes to 1.2 V. (water splitting).^228^ However, designing asymmetric supercapacitors with different materials in each electrode can boost the operational voltage further than 1.2 V even with aqueous electrolytes.^229^

Organic electrolytes benefit from a working voltage of 2.5–3 V and longer life cycles than aqueous electrolytes.^230^ However, organic electrolytes present lower specific capacitance due to lower ionic conductivity than their aqueous counterparts.^231−234^ The potential window can be extended up to 4.5 V by employing ionic liquid (IL) electrolytes. Additionally, their low volatility and flammability can boost the lifetime of the SCs.^231^ Most ILs present high viscosity and expensive costs, limiting their practical applications.^231,232^

Generally, the design of SCs with gel or solid electrolytes is preferred to increase the cycling stability, mechanical strength, and flexibility of the devices.^235^Table 3 presents typical examples of the capacitance parameters reached by supercapacitors employing different solvents for the electrolyte. Several binders and polymers can be employed to improve the contact of the hydrogels with the surface of the electrode, such as alginate, carboxy methyl cellulose (CMC), xanthan gum, chitosan, polyethylene glycol (PEO), poly(methyl methacrylate) (PMMA), poly(acrylic acid) (PAA), poly(amine-ester) (PAE), poly(vinyl alcohol) (PVA), among others.^236,237^

**Table 3:** Capacitance Parameters of Supercapacitors Employing Aqueous (aq), Organic (org), Ionic Liquid (IL), and Solid-State Electrolytes (SSEs)

Electrolyte	Electrode material	Voltage (V)	Capacitance (F g^–1^) (Current, A g^–1^)	Power (kW kg^–1^)	Retention (%) (cycles)	Ref
H_2_SO_4_ 0.5 M (aq)	RuO_2_/G	1.2	479(0.25)	0.6	94 (1000)	(238)
H_2_SO_4_ 1 M (aq)	PANI/RGO/CeO_2_	1.7	684(1)	0.85	92(6000)	(239)
H_2_SO_4_ 1 M (aq)	RuO_2_	1.5	150(1.25)	0.937	95 (10000)	(240)
KCl 3 M (aq)	PPy/ASA	3	804 (2)	6	93.6(5000)	(241)
Na_2_SO_4_ 1 M+ K_3_Fe(CN)_6_ 0.3 M (aq)	CoMoP_4_@MnO_2_/Ni	2.4	116.7 (1)	0.984	96.8(10000)	(242)
Na_2_SO_4_ 1 M (aq)	C@Mn_3_O_4_	2.7	109 (1)	1.35	94.2(6000)	(243)
LiTFSI 3 m + PEO 30g/L (aq)	AC	2.4	125(0.5)	11.45	92(10000)	(244)
KOH 1 M + Choline 0.1 M (aq)	SC-Se-750-M	1.3	48.9(0.5)	20.4	94.1(10000)	(245)
LiClO_4_ 1 M (aq)	MnO_2_–NPG	1.8	193(2)	25	85(2000)	(246)
NaClO_4_ 17 m (aq)	AC	2.3	33.0 (1)	16.7	85(20000)	(247)
LiTFSI 0.5 M (GBL) (org)	MnO_2_/RGO/CNT	2	41 (0.1)	6.3	35(5000)	(248)
TEABF_4_ 0.7 M (AND) (org)	AC	3.5	20	3.1	74 (35000)	(249)
EMIM-TFSI (1 M) (ACN) (org)	Mo_2_Ti_2_C_3_	3	152	22	86(5000)	(250)
SBPBF_4_ 1 M (EC:MPN) (org)	AC	2.3	104	–	98(1500)	(251)
SBPBF_4_ 1.5 M (PrC) (org)	AC	3.5	122(0.1)	6.938	59.5(2500)	(252)
TEMABF_4_ 1 M (PrC) (org)	AC	3.2	104(0.1)	–	44.2(2500)	(253)
LiPF_6_ 1 M (EC:DEC) (org)	AC	3	126(1)	2.243	–	(254)
NaPF_6_ 1 M (EC:DMC:PrC:EA) (org)	C(Mo_2_C)	3.4	120	90	–	(255)
DmFc 0.2 M + TBAClO_4_ 1 M (THF) (org)	CNTs	2.1	61.3(1)	1.04	88.4(3000)	(256)
EMIMBF_4_ (IL)	G	3.5	192(5)	–	90(1E6)	(257)
EMIMFSI (IL)	AC	3	120(0.5)	ND	90(5000)	(258)
PMPFSI (IL)	AC	3.5	98(0.5)	ND	80 (5000)	(258)
P_4444_FuA (IL)	AC/CNTs	3	10(1)	13.3	82(1000)	(259)
BMIMPF_6_ (IL)	Csponge	4	290(0.1)	ND	90(5000)	(260)
[EMIM+TMA]^+^[BF_4_]^−^ (IL)	AC	3.5	182(1)	7.5	84(5000)	(261)
BQ/PYR_14_TFSI (IL)	AC	3	156	ND	50(1000)	(262)
DEMEBF_4_ (IL)	AC	2.5	25.4		85(5000)	(263)
PVA/LiCl (SSE)	NiCo_2_S_4_/CF	0.7	360	0.3	90(5000)	(264)
PVA/KOH (SSE)	CuMnO_2_	0.7	272(0.5)	7.56	80(18000)	(265)
PHPA/LiClO_4_ (SSE)	AC	2.5	111(0.25)	6.51	80(11000)	(266)
PVA/H_2_PO_4_ (SSE)	N-carbon	1	260(0.5)	5.8	86(10000)	(267)
PANI/Zr-MOF (SSE)	AC	0.8	647(1)	1	91(5000)	(268)
PEGBEM-*g*-PAEMA/EMIMBF_4_ (SSE)	AC	2	55.5(1)	0.9	75(5000)	(269)
PEO-NBR/EMIMTFSI (SSE)	G	2.5	208(1)	5.87	93.7(10000)	(270)
PVDF-*co*-HFP/EMIMNTf_2_ (SSE)	AC	2.5	153(0.05)	6.25	97(10000)	(271)
PVDF/TEABF_4_ (SSE)	G	3	28.46(1 mA)	7.5	91(10000)	(272)
TBAPF_6_/PMMA/PrC/ACN (SSE)	CNTs	2	34.2(0.63)	21.1	94(500)	(273)
PEO/MgTFSI/PrC (SSE)	AC	2	22.3(0.1)	6	100(2000)	(274)

The membrane, whose primary function is to separate the electrodes to prevent the stored charges on each electrode from recombining and avoid short circuits, is another crucial component of a well-performing SC. Membranes must be nonconductive and offer minimal resistance to the diffusion of ions in the electrolyte. They must be chemically and thermally stable, inert to electrode materials and species or redox mediators in the electrolyte, and thermally stable. A high level of mechanical stability is essential to prevent shrinking. In addition, wettability is crucial because it affects the stability under lengthy cycles of the SC unit, affecting the internal resistance and, consequently, the lifetime of the SC.^228,275−277^

The membrane’s pore size impacts its conductivity and affinity for the electrolyte solvent. Good swelling can be obtained with pore sizes around 1 μm with an overall porosity between 40 and 60%.^275^ Several materials have been used as membranes for SC, such as glass, different kinds of paper, ceramic materials, ion-exchange membranes such as Nafion, PVDF, PVDF-HP, polyethylene (PE), polypropylene (PP), cross-linked polymers, chitosan, cellulose, and chitin among others.^275,278^ The membrane thickness should be lower than 25 μm, but thinner films may not have mechanical strength and present low cyclability. Employing some of the solid electrolytes mentioned above can avoid the need for membranes acting as a separator between the electrodes.^230^

### 3.3 Integration of Photocapacitors

As previously discussed, photocapacitors (PCs) can harvest light by employing a photovoltaic unit. So far, third/next-generation photovoltaics (DSC, QDSC, OSC, PSC) have been demonstrated to be promising candidates to design photocapacitors in a vast range of architectures, including rigid and flexible substrates, thereby expanding the range of potential applications to include wearable and portable electronics.^279−281^

**Figure 4:**
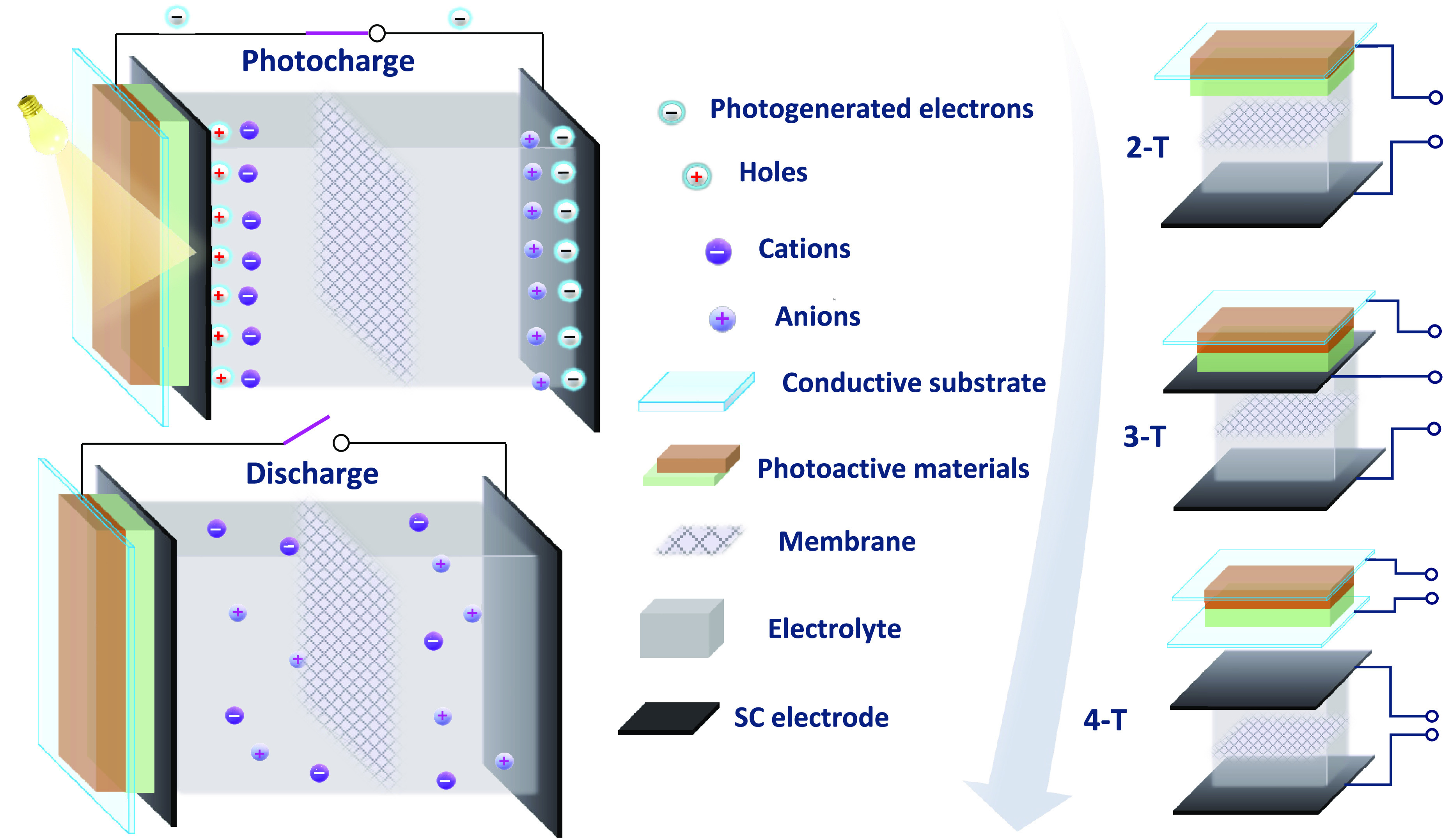
Schematic representation of the photocharging and dark discharge processes in a photocapacitor, and integration of a PC into different terminal configurations: 2-terminal, 3-terminal, 4-terminal.

The main components of a PC are depicted in Figure 4. The PV unit requires a transparent conducting substrate (TCO, e.g., ITO or FTO) that can be rigid or flexible. The photoactive layers are deposited on the substrate, followed by the deposition of the redox couple, gel electrolyte, or a hole transport layer (HTL) such as spiro-OMeTAD. Depending on the PC configuration, the counter-electrode (CE) of the PV unit can also be shared with the SC unit. The CE should preferably be a conducting material coated onto a current collector, such as transparent conductive oxides (TCOs), carbon paper or foam, or any inert metal foam. The SC electrode is usually a “Janus”-type electrode with the capacitive material (carbon-based, metal oxides, chalcogenides, conducting polymers, among others) coated over the rear side. The bottom electrode of the supercapacitor unit serves as the energy storage layer. The electrodes are separated by a membrane, which is essential to prevent charge recombination in the SC. The SC device is filled with an ion-conducting electrolyte and sealed.

The photocharging of the device is induced by light absorption by the PV unit. Charge separation occurs in the photoactive material (dye, organic semiconductor, QD, or metal-halide perovskite). The photogenerated electrons are injected from the LUMO/conduction band (CB) of the dye/QD/perovskite into the CB of the metal oxide (e.g., TiO_2_) and subsequently transported to the current collector. The holes in the HOMO/valence band (VB) of dye/QD/perovskite are scavenged by the hole transport layer (HTL) or reduced by the species in the redox electrolyte. Photocharging requires connecting the photovoltaic electrode to the supercapacitor unit and storing the photogenerated electrons as charge, balanced by cations from the SC unit’s electrolyte. The dark-discharge is achieved by disconnecting the photovoltaic unit from the supercapacitor part of the integrated device. This procedure transfers the stored energy to an external load, supplying power to an electronic device. A rectifying diode should be installed between the PV and SC units to prevent charges from recombining with the PV unit rather than the external load. However, most investigations on photocapacitors avoid utilizing the rectifying diode since it causes a voltage drop of several hundred millivolts. To advance the rapidly expanding field of photocapacitors, developing novel materials or PC designs with a diode-like terminal and minimal voltage loss is essential. To maximize the benefits of a photocapacitor, it is essential to extract all charges during the dark discharge through an external circuit that delivers the power needs of an electronic device, thereby avoiding the supercapacitor’s self-discharge to the electrolyte. Table 4 summarizes the photovoltaic and capacitance parameters of photocapacitors incorporating different photovoltaic technologies for the light-harvesting unit and various capacitive materials for the energy-storage unit.

PCs can be categorized based on the wiring between their components (PV and SC). The most immediate strategy to charge an energy storage system (such as batteries and supercapacitors) by an energy generation system (solar cell, thermo/piezo/tribo-electric device, among others) is to connect the two individual devices through an external wire in a 4-terminal (4-T) configuration as depicted in Figure 4.

**Table 4:** Comparison of Photocapacitors with Hybrid Photovoltaics and Various Supercapacitor Materials

PV type	PCE (%) (sun)	Supercapacitor electrode	Electrolyte	Voltage (V) (sun)	Dark discharge (s)	Capacitance (mF cm^–2^)	Ref
Si	17.8 (1)	a-MoOx	NaSO_4_ 0.1M	0.6 (0.6)	330	34	(284)
Si	–	Si	EMIMBF_4_/PC/PEO	0.58 (0.65)	∼8	0.014	(306)
Si	15.69 (1)	RGO	SiO_2_-BMIMTFSI	0.38 (1)	>10 day	0.0002	(307)
SiNW/PEDOT:PSS	13 (1)	PPy	H_3_PO_4_/PVA	0.55 (1)	>40000	234	(308)
SiNW/PEDOT:PSS	12.37 (1)	G	H_2_SO_4_ 1M	0.5 (1)	12	16.37	(309)
DSC	0.048	CNTs/MnO_2_	TEABF_4_ 1M/ACN	0.932 (1)	∼380	13.1	(310)
DSC	9.5 (1)	MoS_2_	H_3_PO_4_/PVA	∼0.65 (1)	∼25	18.51	(311)
DSC	–	AC	TEABF_4_ 15 wt %/PC	0.45 (1)	∼1500	690	(38)
DSC	6.10 (1)	CNTs/PANI	H_3_PO_4_/PVA	0.72 (1)	144	–	(296)
DSC	4.9 (1)	Co-NiO_*x*_	KOH 1M	0.8 (1)	∼270	32 F g^–1^	(31)
DSC	4.37 (1)	PEDOT	LiClO_4_ 0.5M/MPN	0.69 (1)	150	520	(312)
DSC	3.17 (1)	TiO_2_	Li_2_SO_4_ 2M	0.61 (1)	∼18	1.289	(313)
DSC	2.8 (1)	RGO	BMIMTFSI/THF	0.6 (1)	20	0.14	(314)
DSC	–	PEDOT/CNTs	LiOTf/PC	0.88	67	610	(315)
DSC	2.4 (1)	PPy/RGO	KOH/PVA	0.5 (1)	∼20	124.7 F g^–1^	(316)
DSC	2.25 (1)	AC	Pyr_14_TFSI/PEO/benzophenone	2.45 (1)	1000	–	(317)
DSC	2.25 (1)	AC	Pyr_14_TFSI	2.45 (1)	∼1.75 h	–	(318)
QD/DSC	6.11 (1)	PEDOP/MnO_2_	PMMA/BMIMOTf 1M/PC	0.72 (1)	132	183 F g^–1^	(294)
QDSC	2.75 (1)	NiCo-MOF	S 1M/Na_2_S 1M	0.83 (1)	∼175	588	(319)
QDSC	3.45 (1)	CNTs	PMMA/LiOTf/PC	0.5 (0.1)	75	150 F g^–1^	(293)
QDSC	1.83 (1)	Ni/C	Na_2_S 0.8M/S 0.8M/KCl 2M	0.16 (1)	140	–	(288)
QDSC	3.94 (1)	AC	KOH 2M	0.62 (1)	>120	132.83	(295)
QDSC	–	PEDOT	HEMIMBF_4_/PVP	0.33 (1)	55	0.667	(320)
OSC	1.01 (1)	CNTs	H_3_PO_4_	0.4 (1)	∼310	77 μF cm^–1^	(321)
OSC	3.44 (1)	PEDOT:PSS	H_3_PO_4_/PVA	0.80 (1)	100	–	(141)
OSC	1.8 (1)	CNTs	NaCl 1M	0.92 (388 lx)	3.57 h	–	(322)
OSC	3.39 (1)	CNTs	H_3_PO_4_/PVA	0.6 (1)	∼35	28 F g^–1^	(299)
OSC	7.85 (1)	RGO	H_3_PO_4_/PVA	0.727 (1)	∼110	144 F g^–1^	(297)
OSC	9.75 (1)	PEDOT:PSS/CNTs	H_2_SO_4_/PVA	0.734 (1)	183	250	(323)
OSC	2.5 (1)	Ti_3_C_2_T_*x*_	TT/PEGDA/EMIMTFSI	0.8 (1)	∼320	410 F cm^–3^	(324)
OSC	1.57 (1)	G	TEABF_4_/PC	2.3 (1)	240	–	(325)
OSC	6.7 (1)	PEDOT:PSS/Ti_3_C_2_T_*x*_	H_2_SO_4_ 1M	3 (45k lx)	250	93	(326)
PSC	11 (1)	AC/RGO-PEDOT	H_2_SO_4_/PVA	0.9 (1)	>400	71.54	(327)
PSC	7.79 (1)	AC/MnO_2_	LiCl/PVA	0.84 (1)	>500	61.01	(289)
PSC	12.5 (1)	N-MC	H_2_SO_4_/PVA	1.0 (1)	>75	31	(282)
PSC	22.44 (1)	AC	KOH 6M	1.1 (1)	211.5	–	(28)
PSC	2.5 (1)	PANI/CNTs	H_2_SO_4_/PVA	0.7 (1)	275	–	(328)
PSC	13.66 (1)	RGO	H_3_PO_4_/PVA	0.91 (1)	∼130	142 F g^–1^	(297)
PSC	8.9 (1)	AC	H_3_PO_4_/PVA	0.91 (1)	44	∼17.5	(329)
PSC	14.14 (1)	AC	KOH/PVA	0.68 (1)	20	13.6 F g^–1^	(330)
PSC	5.6 (1)	Co_9_S_8_-MnO_2_	H_3_PO_3_/PVA	0.63 (1)	70	–	(331)
PSC	6.37 (1)	AC/PEDOT	LiClO_4_/MAI/iPrOH	0.7 (1)	38	12	(332)
PSC	12.54 (1)	MoO_3_	H_2_SO_4_/PVA	0.68 (1)	∼200	43	(333)
PSC	7.79 (1)	AC/MnO_2_	LiCl/PVA	0.84 (1)	∼20	61.01	(334)
PSC	14.13 (1)	RGO	H_2_SO_4_/PVA	0.75 (1)	45	–	(335)
PSC	6.1 (1)	NC	TEOS/TEABF_4_/H_3_PO_4_	1.2 (1)	∼50	–	(336)

Even though in a 4-T arrangement, each unit can be improved separately, this strategy presents some drawbacks, such as intricate packaging, energy losses due to the resistance of the external wires, and lower overall efficiency.^282^ In addition, such externally connected systems tend to be bulky and not flexible, reducing the range of possible application fields.

Similar to tandem photovoltaics, photocapacitors can be integrated into a 2-terminal (2-T) configuration, often referred as monolithic arrangement, where the materials from the PV unit and SC are stacked together or just or separated by a membrane as shown in Figure 4.^283^ Miyasaka et al. introduced the first 2-T photocapacitor in 2004.^38^ They incorporated a DSC and a carbon-based supercapacitor counter-electrode. The system was photocharged at 1 sun illumination to a voltage of 0.45 V and showed a capacitance of 0.69 F cm^–2^.

The 2-T layout has the benefit of requiring fewer materials and substrates. Due to the limitations of the 2-terminal structure, which include low charging voltage, high resistance, low charge–discharge efficiency, and self-discharge to the photoactive side of the device, 2-T architectures are typically not employed.^284^ These limitations prompted the development of the 3-terminal (3-T) architecture, in which the PV and SC devices share a common electrode but operate independently.^284^

The early 2-T structure, adopted in 2004 in the first demonstration of a photocapacitor with a DSC as the photogeneration device,^38^ was rapidly surpassed by the more effective 3-T design.^29^ As a result of improved electron and hole transfer in the charge–discharge process, the 3-T design showed a photocharged voltage under 1 sun of 0.8 V and energy density of 47 μWh cm^–2^. Since then, a plethora of materials and configurations have been investigated.^14,285−288^

Bagheri et al. developed a 3-T PC employing a DSC and an asymmetric SC with cobalt-doped nickel oxide (Ni(Co)O_*x*_) and AC as positive and negative electrodes.^31^ The PC generated a photovoltage of 0.8 V and overall energy conversion of 0.6% when exposed to 1 sun illumination for 500 s. A monolithic PC with a perovskite light harvester and a carbon-based supercapacitor was reported by Liu et al.^289^ Under 1 sun illumination, the integrated stacked device achieved a voltage of 0.84 V with an overall conversion efficiency of 5.26% and an energy storage efficiency of 76%.

Skunik-Nuckowska et al. developed a 3-T PC employing a solid-state DSC and ruthenium oxide as the intermediate electrode and ruthenium oxide/FTO as the second supercapacitor electrode.^290^ The system delivered 3.26 F cm^–2^ with Coulombic efficiency of 88% and a charged state at 0.88 V when illuminated at 1 sun. A 3-T Pc employing a symmetric PProDOT-Et_2_ supercapacitor and an N3-based DSC was reported by Hsu et al.^291^ The PC exhibited a photocharged voltage of 0.75 V under 1 sun illumination, with a capacitance of 0.48 F cm^–2^ and an energy density of 22 uWh cm^–2^. A double-sided electrodeposited PPy/RGO as an intermediate electrode in a 3-T architecture was employed by Lau et al.^292^ The PC delivered 124.7 F g^–1^ under 1 sun illumination, with a retention of 70% after 50 consecutive cycles.

Narayanan et al. reported an integrated 3-T PC with a plasmonic QDSC delivering a 3.45% PCE. They employed a functionalized MWCNT symmetric EDL supercapacitor delivering a specific capacitance of 150 F g^–1^ and reached a photocharged voltage of 0.5 V under 0.1 mW cm^–2^ illumination.^293^

Das et al. presented another 3-T PC design, combining a QDSC (with CdS QDs and hibiscus dye as sensitizers) with a bifunctional poly(3,4-ethylenedioxypyrrole) (PEDOP)@manganese dioxide (MnO_2_) electrode as CE and symmetric supercapacitor.^294^ The solar cell unit yielded a PCE of 6.11%. The PC reached a photocharged voltage of 0.72 V under 1 sun illumination and delivered 183 F g^–1^ and power density of 360 W kg^–1^ at a discharge current density of 1 A g^–1^. Zheng et al. recently employed a similar device architecture in a PC that combines a CdS/CdSe QDSC and an AC-based supercapacitor with a common electrode.^295^ Under 1 sun, the QDSC delivered a 3.94% PCE and could charge the supercapacitor up to a voltage of 0.62 V. The integrated device achieved 2.66% overall efficiency and demonstrated good stability, retaining 76.7% of its initial overall efficiency after 100 charge and discharge cycles.

The development of solid-state photocapacitors will accelerate the widespread adoption of IoT applications that are economically viable on a commercial scale. An all-solid-state 3-T integrated device using a DSC and an MWCNT-based supercapacitor, with a PVA/H_3_PO_4_ gel electrolyte, was reported by Yang et al.^296^ The PC was photocharged to 0.72 V under 1 sun illumination with an energy storage efficiency of 84%. Adding polyaniline (PANI) to the MWCNT film boosted the specific capacitance from 48 to 208 F g^–1^. An alternative solid-state photocapacitor was developed by Kim et al. They monolithically integrated a high-performance OSC PV unit based on PTB7-Th/PC71BM, with a carbon-based SC and a PVA/H_3_PO_4_-based solid-state electrolyte. The PC achieved a storage efficiency and overall efficiency of 64.59% and 5.07%, respectively, with a photocharged voltage of around 0.9 V under 1 sun illumination.^297^

Another solid-state photocapacitor with a PSC as a PV unit and a PEDOT-carbon composite material for the common electrode was reported by Xu et al.^298^ The PC delivered a maximum overall energy conversion and storage efficiency of 4.7%. Liu et al. integrated a PSC and a solid-state supercapacitor on the same FTO glass substrate through a common carbon-based electrode,^289^ reaching an energy storage efficiency of 76% and an overall efficiency of 5.26%. Combining a PVA/H_3_PO_4_-based solid-state supercapacitor and a PSC with 1 cm^2^ active area, Kim et al. achieved a high storage efficiency of 80.3% and overall efficiency of 10.97%.^297^

The highest overall efficiency for a quasi-solid state photocapacitor integrating a PSC reached 11.5% in a monolithic stacked architecture with an N-doped carbon SC. This remarkable overall efficiency derives from the high PCE of the solar cells, i.e. 12.5%, the high storage efficiency of the supercapacitor, i.e. 92%, and the minimized internal energy losses due to the monolithic integration.^282^ In general, the performance of solid-state photocapacitors is poor. This is because gel and polymer electrolytes have limited conductivity, which reduces capacitance and charge storage efficiency. Future research should concentrate on creating solid electrolytes with enhanced conductivity and electrode-wetting properties.^299^

Low ambient light levels are anticipated for many applications of photocapacitors, such as sensors for the Internet of Things and wearable electronics. The spectrum of ambient light varies depending on the source, so the design of photocapacitors must account for the IoT application’s power requirements and match the spectrum of the nearby light source. Lechêne et al. reported an example of PCs optimized for indoor operation. They designed a 4-T PC based on an OSC with PCDTBT:PC71BM and a carbon-based SC. The overall efficiency increased from 1.57% under 1 sun to 2.92% under simulated indoor lighting of 310 μW cm^–2^ from a CFL lamp.^300^ A similar improvement with indoor illumination was observed by Jin et al.^301^ They employed an OSC module (ITO/ZnO/PBDB-T:ITIC) with PEDOT:PSS and an asymmetric supercapacitor sharing the PEDOT:PSS electrode. The integrated photocapacitor achieved a photocharged voltage of 1.5 V under 1000 lx from a LED light source with an integrated power of 304.6 μW cm^–^.^2^

Typically, the photocharged voltage measured in the storage unit is below 1 V. This voltage might be insufficient to power most applications, including sensors or low-power electronics. An effective strategy to get higher voltages is to connect several PV units in series, design an integrated PC with PV and SC modules, and employ microsupercapacitors with higher surface areas. Sun et al. designed a 2-T configurated photocapacitor delivering a high energy density (up to 32.3 μWh cm^–2^) and high output voltages of around 2 V.^106^ The system employed a PSC integrated with a micro-SC. The power pack powered an array of red and white LEDs, a micromotor, and a timer. Gao et al. reported a 3-T integrated system delivering an open-circuit voltage up to 1.8 and 4.7 V for an array of three PC in series, with an energy density of 0.18 Wh m^–2^, and an overall efficiency of 7.0%.^39^ In a 3-T planar configuration on rigid substrates, Xu et al. combined a CH_3_NH_3_PbI_3_-based PV with a polypyrrole-based supercapacitor; the energy pack had an open circuit voltage of 1.45 V and an overall efficiency of 10%.^302^

Scalia et al. demonstrated high-voltage (2.45 V) photocapacitors enabled by four series-connected DSC modules with an IL electrolyte and activated carbon SC unit.^303^ Chien et al. used a string of 8 OSCs based on the P3HT:PC60BM bulk-heterojunction structure, connected in series on the same ITO glass substrate to increase the *V*_OC_ up to 5 V. The photovoltaic unit was integrated with graphene-based supercapacitors, providing about 2.5 mF cm^–2^ of capacitance on the same substrate, with graphene as the common electrode.^304^ A different configuration was proposed by Dong et al.,^305^ in which a flexible printable DSC and a supercapacitor with reduced graphene oxide electrodes and a polymer electrolyte were fabricated side by side on the same PEN-ITO substrate. The device showed charging potentials up to 1.8 V and exceptionally stable performance under various bending and tilting tests in outdoor conditions.

Song et al. obtained the highest overall efficiency to date among all reported investigations on various photocapacitor configurations. They presented a novel approach to manufacturing photocapacitors with low-loss energy storage. The PC was charged to 1.1 V under 1 sun illumination and demonstrated a storage efficiency of 20.53% and an overall efficiency of 18.34%.^28^ Future research on engineering should combine cutting-edge materials with lower energy loss integration methods between the PV and SC units, enhancing performance beyond 25% under direct sunlight and 40% under ambient light conditions.

## 4 Methods and Techniques for Characterizing Photocapacitors

When describing and characterizing the performance of a photocapacitor, several aspects come into play, ranging from the overall efficiency of the photocapacitor to the system operating voltage. Each parameter ultimately defines the potential application scenario or optimization pathway of a photocapacitor. In this section, we discuss the characterization of the PV and SC units, the behavior of the integrated PC device, and the need for a protocol to measure and report results in the growing photocapacitor area.

### 4.1 Performance Assessment

**Figure 5:**
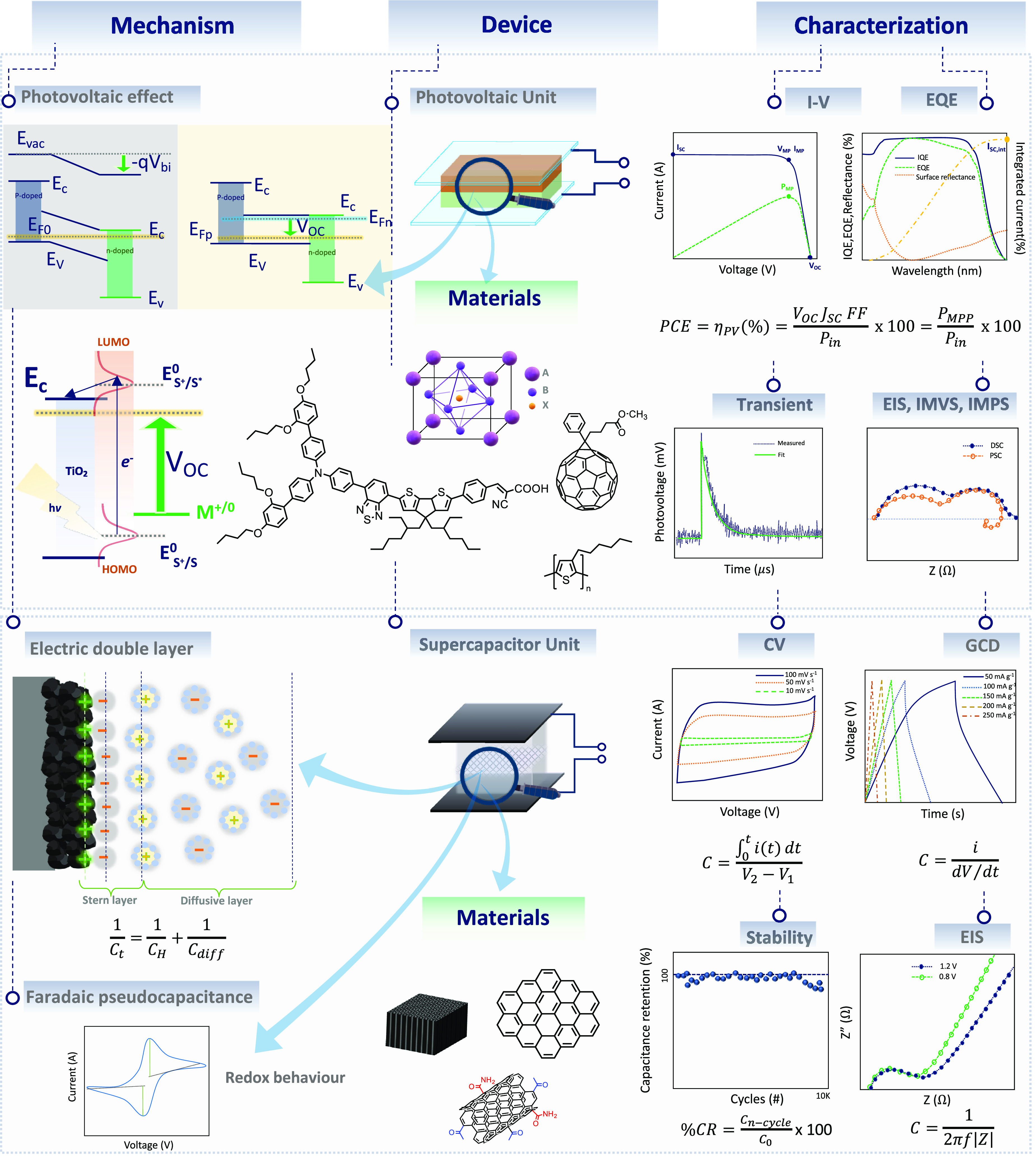
Schematic guideline showing the mechanism, materials, and characterization techniques of photovoltaic and supercapacitor devices.

The overall efficiency of the integrated photocapacitor is derived by multiplying the independent efficiencies of the solar cell and supercapacitor components.^280^Equation 2 is used to determine the power conversion standard current–voltage measurements (*I*–*V* curves).
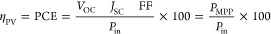
2where *V*_OC_, *J*_SC_, FF, and *P*_in_ represent the open-circuit voltage, the short-circuit current density, the fill factor, and the incident light power density, respectively. As shown in Figure 5, *V*_OC_ is the maximum voltage at open circuit conditions (at *J* = 0). At the same time, the *J*_SC_ denotes the highest current attainable by the solar cells when the applied bias is 0. Several techniques, such as external quantum efficiency (EQE), transient measurements, electron impedance spectroscopy (EIS), intensity modulated photovoltage spectroscopy (IMVS), intensity modulated photocurrent spectroscopy (IMPS), can be employed to characterize the behavior of photovoltaic cells.^337^

In determining the total efficiency of the integrated PC module, the capacitance of the SC also plays a significant role. The capacitance of a charged electrode can be estimated by the contributions of the Helmholtz double layer. Following the Stern model, the ions are assumed to be point charges attracted by electrostatic forces in the inner layer. In the near vicinity, the diffusive layer is composed of solvated anions and cations that counterbalance the charged electrode. In eq 3 C_H_ and C_diff_ represent the inner layer and diffusive layer capacitance, respectively.
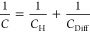
3

The specific capacitance can be calculated from different electrochemical methods. The mathematical equations below (eqs 4–6) deliver the specific capacitance obtained from galvanostatic charge–discharge measurements, cyclic voltammetry, and electrochemical impedance spectroscopy (EIS), respectively.^226,338^
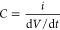
4
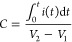
5
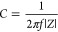
6

In eq 4, *i* represents the constant current applied during the GCD measurement and d*V*/d*t* represents the slope of the GCD discharge. In eq 5, the capacitance is obtained by integrating the typical *I*–*V* voltammogram, divided by the potential window. Alternatively, the capacitance can be determined from EIS measurements, where in eq 6*f* represents the frequency and *Z* the real part of the impedance. The capacitance of a symmetric supercapacitor with equal mass loading of the same material can be obtained from measurements on a single electrode as shown in eq 7.
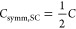
7

The following eqs 8–10 can be used to calculate the output energy (*E*_output_), the energy density (*E*_A_, in Wh g^–1^) normalized by the mass loading, and the power density (*P*_A_, in W g^–1^) of the supercapacitor during charging, where *V* represents the potential window of the SC and *R* the internal resistance of the supercapacitor.
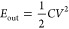
8
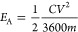
9
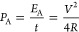
10

The total efficiency of the combined photocapacitor module can be calculated using eq 11, where *A*_PV_ is the solar cell’s surface area and *E*_in_ is the amount of energy received during charging (eq 12). The overall efficiency is calculated by dividing the energy produced during illumination by the energy stored during photocharging.^97,282,339^
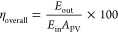
11
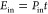
12

The overall efficiency of a PC is determined by the photoconversion efficiency, the supercapacitor performance, and the interface between them, which is crucial for advanced stacked systems. Assuming that the supercapacitor unit presents a constant capacitance, the maximum efficiency of a photocapacitor system is proportional to the solar cell’s PCE. Therefore, an efficient solar cell is a prerequisite for an efficient photocapacitor system. The Shockley-Queisser limit determines the performance of a PV unit, which depends on the material’s bandgap as shown in Figure 5.^340^ The photovoltaic community efforts in recent years have resulted in very efficient technologies with efficiencies over 25% under sunlight and indoor PCEs reaching around 40% as shown in Figure 5. The integration of the SC affects the time to reach the maximum value of the PC efficiency, excluding parasitic currents in the supercapacitor unit.^341^ The low overall efficiencies of integrated PCs are mainly caused by energy losses between the PV and energy storage units. Future research should focus on performance coupling and new circuit design.^342^

It is feasible to enhance the performance of the energy storage device by increasing its energy and power density. Hybrid SCs that employ pseudocapacitive materials to build asymmetric SCs can provide superior energy storage performance. Additionally, increasing the effective surface area of electrode materials can boost the energy density of SCs.^343^ An increase in surface area can be achieved by improving the electrode’s 3D porous structure to maximize the electrode/electrolyte interface (wettability), ion diffusion, and charge transfer.^344^ Nanoporous materials with pore sizes between 2 and 50 nm can reach high capacitance and effective ion pathways for fast kinetics, making them ideal for surface utilization and performance enhancement.^282^ EIS measurements can be implemented to gain a better understanding of the interfacial process and estimate energy losses in the PV and SC units, and for proposing further approaches to decrease the energy losses in the integrated device.

Optimizing the PV unit materials, structure, and interfaces reduces bulk and nonradiative recombination and increases charge transfer. Additionally, optimizing the architecture and arrangement of the integrated devices can reduce the series resistance of stacked or parallel-connected devices, reducing energy losses. Monolithic devices with a common electrode reduce energy losses and provide enough energy storage and charge extraction for high energy conversion efficiency and reliability.^345^ The absence of wiring between the two devices simplifies the architecture and reduces the PC’s internal resistance.^299^

### 4.2 Operating Voltage of the Integrated Devices

The voltage of a supercapacitor device is determined by the charge separation at each electrode, resulting in a potential difference across the whole device.^346^ Techniques such as CV and GCD can measure the maximum operating voltage of supercapacitor materials in a three-electrode setup or on entire devices (2-electrode setup).^327,346^ However, maximal voltage testing can result in cell damage. An alternative option is to apply a lower voltage to the device and gradually increase it until a change appears at the potential window’s edge. As discussed earlier in section 3.2, the electrolyte solvent and the supercapacitor’s design significantly influence the operating voltage of the SC. For instance, the thermal breakdown potential of water at room temperature restricts the operating voltage of SCs employing aqueous electrolytes to values lower than 1.2 V.^225^ In traditional symmetric supercapacitors, the electrode materials and mass loading are identical; as a result, its stable potential window includes only a narrow potential range. Using the differing potential windows of asymmetric electrodes can maximize their working voltage during the charge and discharge cycles to voltages greater than 2 V.^225,347^ Employing organic solvent electrolytes can expand the potential window of supercapacitor devices between 2.3 and 4 V. Using room temperature ionic liquids, the potential window can further shift from 3 to 6 V.

The open-circuit voltage of a PV unit depends on several components but mainly on its technology. For instance, in a DSC the *V*_OC_ depends on the energy difference between the Nernst redox potential (*E*^0^) of the redox mediator and the quasi-Fermi level (*E*_F,q_) of the electrons in the TiO_2_. At the same time, this is dependent on the photocurrent and the electron recombination rates.^348−352^ For OPVs, the *V*_OC_ depends on the energy difference between the HOMO (highest occupied molecular orbital) of the donor molecule and the LUMO (lowest unoccupied molecular orbital) of the acceptor molecule and also relies on the external quantum efficiency (EQE).^353−358^ The *V*_OC_ of PSCs depends on the bandgap of the photoactive material (perovskite)^359,360^ and is greatly affected by defects and traps.^361−363^

**Figure 6:**
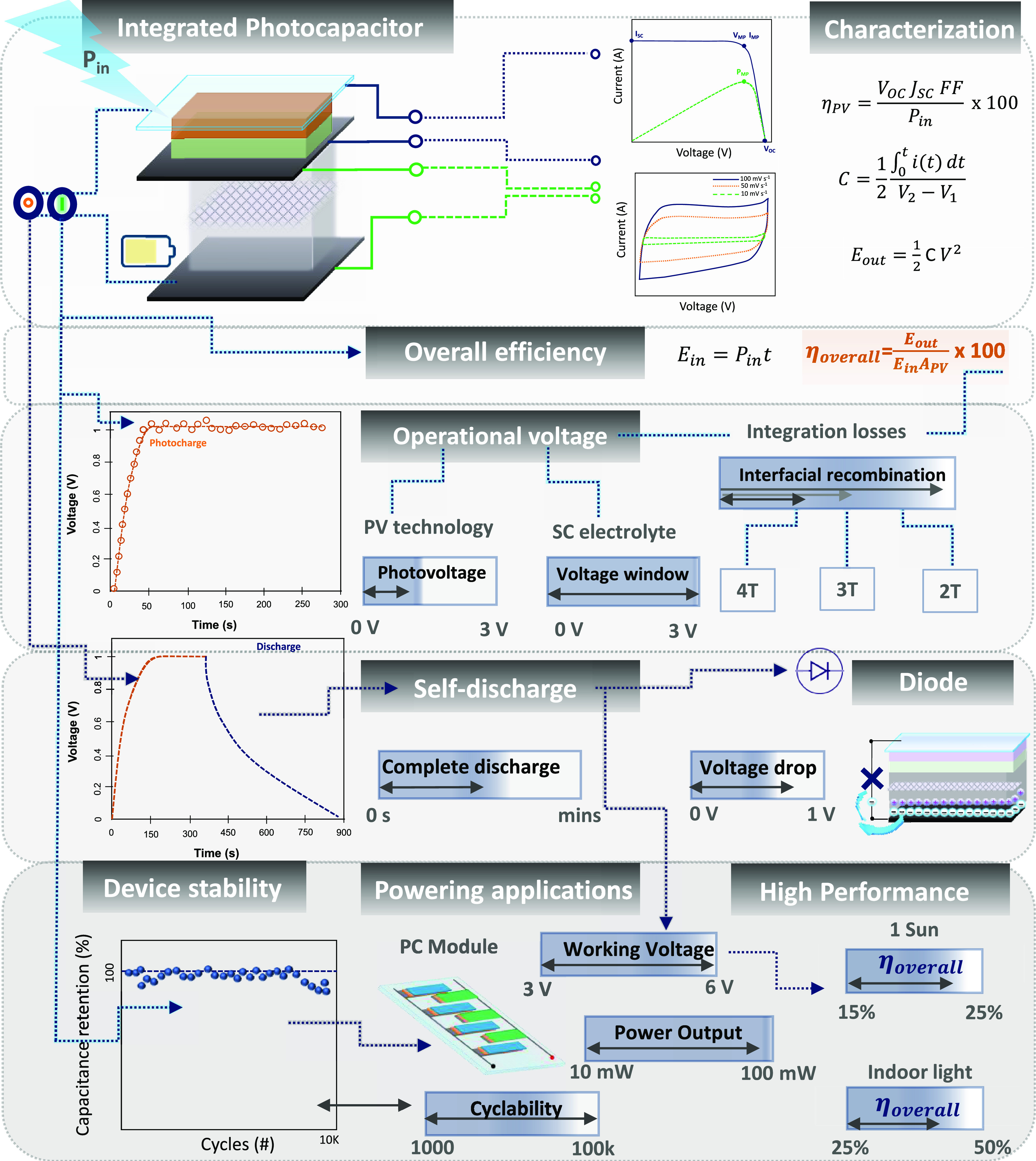
Schematic overview of the characterization of an integrated PC. The photovoltaic and charge storage efficiencies must be measured for the entire system to calculate a PC’s overall efficiency. The dark discharge must be recorded under strict dark conditions, and the stability must be reported following multiple photocharge–discharge cycles.

The supercapacitor unit essentially determines the operating voltage of an integrated PC in an ideal photocapacitor with minimal energy losses. This can be inferred since the current densities transferred from the PV to the SC during the photocharging process are sufficient to reach the supercapacitor’s maximum voltage (Figure 6). However, most PC studies show operating voltages lower than the SC unit’s capability. This voltage loss can be attributed to energy losses at the interface or wiring between the PV and SC unit or recombination of the stored charges in the SC unit with the PV unit. As mentioned earlier, charge recombination from the SC to the PV unit can be avoided by connecting the PV and SC through a rectifying diode. Unfortunately, the diode will inherently cause a voltage drop across the system, compromising the integrated PC device’s ultimate voltage output. New approaches must be studied in the future to develop PCs able to exploit the maximum voltage range of the SC unit.

Using photocapacitors to power electronic devices will require a constant operating voltage of 3–6 V, requiring the connection of various SCs in series to increase the operating voltage of the individual units. Overcharging may harm the SC’s lifespan, and a malfunctioning unit may influence the output voltage. This emphasizes the urgency to design photocapacitors with high voltage ranges.^280,302,343^ Moreover, one of the most significant difficulties that must be addressed is the design of PC devices that can maintain a constant voltage for longer discharging intervals than a few minutes, as shown in the majority of photocapacitors described in the literature.

One approach to increase the discharging time of a photocapacitor is to employ hybrid supercapacitors with a capacitive electrode and a battery-type electrode with ion intercalation. This strategy can boost energy storage and discharge times to achieve the needed 6–8 h dark lapse duration when ambient light is not available. One approach to greatly enhance the energy density of the supercapacitor unit is the design of a sodium/potassium/zinc or magnesium-ion supercapacitors, which can reach a high energy density ranging from 5 to 200 Wh kg^–1^ and high power from 0.1 to 30 kW kg^–1^.^113,343,364−370,226,227^ A wide variety of materials such as MoO_2_, Fe_4_O_4_, Li_4_Ti_5_O_12_ (LTO), and carbon-based materials doped with Li-ions, among others, can be employed for ion intercalation and thus can be employed to develop hybrid SCs.^371,372^

### 4.3 Protocol and Standardization

While standard methods for PV characterization and electrochemical procedures for SCs are well established, the new field of integrated photocapacitors requires a standardized methodology for evaluating and reporting the integrated devices’ energy harvesting and storage properties.^281^ Regardless of the photocapacitor’s architecture, the independent photovoltaic behavior of the PC unit when it is merely harvesting light without being coupled to the SC unit must be reported (*J–V*, IPCE, etc.) as depicted in Figure 6. Additionally, the SC unit’s independent capacitive or pseudocapacitive behavior when it is charged by a potentiostat (CV, GCD, EIS) must also be reported with the electrode’s mass loading, thickness, and active area. These parameters should be compared to the characterization of the complete photocapacitor to address how the photovoltaic and capacitive behaviors change when the PV and SC are coupled. Special attention must be given to the dark self-discharge time after charging the SC unit via light harvesting (photocharging). The photogenerated charges under various ambient light conditions are sufficient to charge the SC (at a lower voltage than under 1 sun conditions). As a result, if strict dark conditions are not followed to study the self-discharge of photocapacitor devices, a “constant” voltage can be measured over an extended period of time.

The capacitance retention and cyclability of the integrated PC device should be investigated, as the vast majority of research only describes the stability of the SC unit. In addition, it is crucial to determine the overall performance throughout a broad temperature and voltage operating range. This information is required to address safety concerns, specifically because energy storage devices can induce heat losses due to the increased resistance of electrodes and connections between PV and SC units and electrolyte degradation during extended charge/discharge cycles.^373^

## 5 Future Outlook

As we look toward the future of photocapacitor technology, a multitude of opportunities and challenges lie ahead.^374^ To unlock the full potential of photocapacitors and ensure their widespread adoption,^375^ future research could encompass key technical issues and continue to push the boundaries of our understanding of the underlying chemistry and materials science.^376−380^

**Figure 7:**
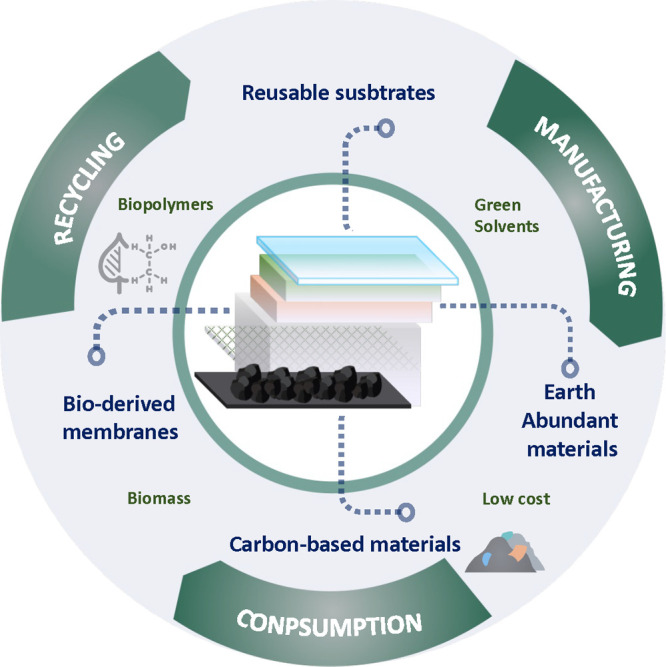
Sustainability of photocapacitors. Through Earth-abundant materials, third-generation photovoltaics can be manufactured with green chemistry techniques. Additionally, the charge storage unit can integrate multiple waste and bioderived materials. These features contribute to the viability of PC production and integration into a circular economy.

One critical area of focus could be the development of advanced materials for both the photovoltaic and supercapacitor components of photocapacitors. Novel materials, such as metal halide perovskites, organic semiconductors, and two-dimensional materials, offer unique properties that could significantly improve the performance and sustainability of photocapacitor devices.^381−391^ Furthermore, exploring the potential of sustainable, abundant, and low-cost materials, such as Earth-abundant metal oxides and sulfides, carbon-based materials, and bioderived polymers, for use in photocapacitor components might be a fruitful direction (Figure 7).^392−399^

Fundamental research will play a vital role in driving the progress of photocapacitors.^115,400^ By gaining a deeper understanding of the underlying chemical reactions, charge transfer mechanisms, and material properties, researchers can optimize device performance and identify novel materials and configurations to further improve the efficiency and stability of photocapacitors. This knowledge will also facilitate the development of predictive models, which can help guide the rational design of photocapacitor devices with tailored properties for specific applications.

Another important avenue of research is the investigation of novel device architectures and integration strategies that enable the efficient coupling of photovoltaic and supercapacitor components. Optimizing the interfacial charge transfer and minimizing energy losses will be crucial for enhancing the overall performance of integrated devices.^280^ The development of innovative fabrication techniques and scalable manufacturing processes will also be essential in making photocapacitors a viable option for a wide range of applications.

The potential of photocapacitors in powering the Internet of Things (IoT) and the Internet of Everything (IoE) is immense. As the electronic industry expands and the demand for portable, sustainable power sources grows, the commercial prospects for photocapacitors become increasingly favorable. The deployment of photocapacitors in self-powered electronic devices could lead to significant societal benefits, such as energy savings, increased use of renewable energy, enhanced connectivity, and improved data transfer.

The integration of ambient photocapacitors with machine learning (ML) and artificial intelligence (AI) technologies offers immense potential for developing self-powered, intelligent systems that can harness ambient light to power the next generation of smart IoT devices. This combination of cutting-edge technologies has the potential to revolutionize various sectors, such as agriculture, health, business, and environmental monitoring, ultimately leading to a more sustainable and connected future.

Ambient photocapacitors, which capture and store energy from ambient light sources, provide a sustainable and distributed energy solution for IoT devices. As electronic miniaturization and energy storage advancements continue, these devices can enable IoT networks to rely on renewable energy sources. Concurrently, ML and AI advancements are transforming data processing, analysis, and decision-making in IoT networks. By embedding these capabilities into photocapacitor-powered IoT devices, we can create intelligent systems that autonomously learn, adapt, and respond to their environment.

The convergence of ambient photocapacitors, ML, and AI allows for the development of self-powered, context-aware devices that actively respond to their surroundings. For example, in agriculture, these smart IoT devices could optimize irrigation and fertilization strategies based on soil conditions, crop health, and weather patterns. In healthcare, wearable devices powered by ambient photocapacitors could continuously track vital signs for early detection of potential health issues.

To fully harness the potential of integrating ambient photocapacitors with ML and AI, future research should focus on refining materials, fabrication methods, and device architectures for photocapacitors, as well as optimizing algorithms and computational resources for on-device ML and AI processing. Overcoming these challenges and fostering interdisciplinary collaboration will drive the development of self-powered, intelligent systems that will shape the future of IoT applications and contribute to a more sustainable, connected world.

In conclusion, the future of photocapacitor technology is bright, with abundant opportunities for innovation, growth, and positive impact. By addressing the key technical challenges, conducting fundamental research, and exploring novel materials and device configurations, we can unlock the true potential of photocapacitors and make them an integral part of our sustainable energy landscape. By exploring new materials, refining device architectures, and integrating with advanced technologies such as machine learning and artificial intelligence, photocapacitors have the potential to reshape numerous sectors and contribute to a more sustainable and connected future. Further research and collaboration across various disciplines will be crucial in unlocking the full potential of this versatile technology.

## References

[ref1] Boukortt N. E. I.; Patanè S. Single junction-based thin-film CIGS solar cells optimization with efficiencies approaching 24.5. Optik 2020, 218, 165240 10.1016/j.ijleo.2020.165240.

[ref2] Bailie C. D.; Christoforo M. G.; Mailoa J. P.; Bowring A. R.; Unger E. L.; Nguyen W. H.; Burschka J.; Pellet N.; Lee J. Z.; Grätzel M.; Noufi R.; Buonassisi T.; Salleo A.; McGehee M. D. Semi-transparent perovskite solar cells for tandems with silicon and CIGS. Energy Environ. Sci. 2015, 8, 956–963. 10.1039/C4EE03322A.

[ref3] Feurer T.; Reinhard P.; Avancini E.; Bissig B.; Löckinger J.; Fuchs P.; Carron R.; Weiss T. P.; Perrenoud J.; Stutterheim S.; Buecheler S.; Tiwari A. N. Progress in thin film CIGS photovoltaics – Research and development, manufacturing, and applications. Progress in Photovoltaics: Research and Applications 2017, 25, 645–667. 10.1002/pip.2811.

[ref4] Wang S.; Tan L.; Zhou J.; Li M.; Zhao X.; Li H.; Tress W.; Ding L.; Graetzel M.; Yi C. Over 24 perovskite solar cells. Joule 2022, 6, 1344–1356. 10.1016/j.joule.2022.05.002.

[ref5] Jeong J.; Kim M.; Seo J.; Lu H.; Ahlawat P.; Mishra A.; Yang Y.; Hope M. A.; Eickemeyer F. T.; Kim M.; Yoon Y. J.; Choi I. W.; Darwich B. P.; Choi S. J.; Jo Y.; Lee J. H.; Walker B.; Zakeeruddin S. M.; Emsley L.; Rothlisberger U.; Hagfeldt A.; Kim D. S.; Gratzel M.; Kim J. Y. Pseudo-halide anion engineering for α-FAPbI3 perovskite solar cells. Nature 2021, 592, 381–385. 10.1038/s41586-021-03406-5. 33820983

[ref6] Zhang D.; Stojanovic M.; Ren Y.; Cao Y.; Eickemeyer F. T.; Socie E.; Vlachopoulos N.; Moser J.-e.; Zakeeruddin S. M.; Hagfeldt A.; Grätzel M. A molecular photosensitizer achieves a Voc of 1.24 V enabling highly efficient and stable dye-sensitized solar cells with copper(II/I)-based electrolyte. Nat. Commun. 2021, 12, 1777 10.1038/s41467-021-21945-3. 33741953 PMC7979847

[ref7] Ren Y.; Flores-Díaz N.; Zhang D.; Cao Y.; Decoppet J. D.; Fish G. C.; Moser J. E.; Zakeeruddin S. M.; Wang P.; Hagfeldt A.; Grätzel M. Blue Photosensitizer with Copper(II/I) Redox Mediator for Efficient and Stable Dye-Sensitized Solar Cells. Adv. Funct. Mater. 2020, 30, 2004804 10.1002/adfm.202004804.

[ref8] Jung H. S.; Park N. G. Perovskite solar cells: From materials to devices. Small 2015, 11, 10–25. 10.1002/smll.201402767. 25358818

[ref9] Green M. A.; Ho-Baillie A.; Snaith H. J. The emergence of perovskite solar cells. Nat. Photonics 2014, 8, 506–514. 10.1038/nphoton.2014.134.

[ref10] Muñoz-García A. B.; Benesperi I.; Boschloo G.; Concepcion J. J.; Delcamp J. H.; Gibson E. A.; Meyer G. J.; Pavone M.; Pettersson H.; Hagfeldt A.; Freitag M. Dye-sensitized solar cells strike back. Chem. Soc. Rev. 2021, 50, 12450–12550. 10.1039/D0CS01336F. 34590638 PMC8591630

[ref11] Wang A.; Zhao J.; Green M. A. 24 Applied Physics Letters 1990, 57, 602–604. 10.1063/1.103610.

[ref12] Rong Y.; Hu Y.; Mei A.; Tan H.; Saidaminov M. I.; Seok S. I.; McGehee M. D.; Sargent E. H.; Han H. Challenges for commercializing perovskite solar cells. Science 2018, 361, 361 10.1126/science.aat8235. 30237326

[ref13] Minggu L. J.; Wan Daud W. R.; Kassim M. B. An overview of photocells and photoreactors for photoelectrochemical water splitting. Int. J. Hydrogen Energy 2010, 35, 5233–5244. 10.1016/j.ijhydene.2010.02.133.

[ref14] Zeng Q.; Lai Y.; Jiang L.; Liu F.; Hao X.; Wang L.; Green M. A. Integrated Photorechargeable Energy Storage System: Next-Generation Power Source Driving the Future. Adv. Energy Mater. 2020, 10, 1903930 10.1002/aenm.201903930.

[ref15] Wang G.; Wang H.; Ling Y.; Tang Y.; Yang X.; Fitzmorris R. C.; Wang C.; Zhang J. Z.; Li Y. Hydrogen-treated TiO2 nanowire arrays for photoelectrochemical water splitting. Nano Lett. 2011, 11, 3026–3033. 10.1021/nl201766h. 21710974

[ref16] Niu F.; Wang D.; Li F.; Liu Y.; Shen S.; Meyer T. J. Hybrid Photoelectrochemical Water Splitting Systems: From Interface Design to System Assembly. Adv. Energy Mater. 2020, 10, 1900399 10.1002/aenm.201900399.

[ref17] Wang H.; Tian Y.-M.; König B. Energy- and atom-efficient chemical synthesis with endergonic photocatalysis. Nature Reviews Chemistry 2022, 6, 745–755. 10.1038/s41570-022-00421-6. 37117495

[ref18] Arcudi F.; D̵ord̵ević L.; Schweitzer N.; Stupp S. I.; Weiss E. A. Selective visible-light photocatalysis of acetylene to ethylene using a cobalt molecular catalyst and water as a proton source. Nat. Chem. 2022, 14, 1007–1012. 10.1038/s41557-022-00966-5. 35681045

[ref19] BINDING D.; STEINBACH F. Homogeneous Photocatalysis by Organic Dyes in the Liquid Phase. Nature 1970, 227, 832–833. 10.1038/227832a0. 16058171

[ref20] Fujishima A.; Rao T. N.; Tryk D. A. Titanium dioxide photocatalysis. Journal of Photochemistry and Photobiology C: Photochemistry Reviews 2000, 1, 1–21. 10.1016/S1389-5567(00)00002-2.

[ref21] Chen B.; Meng Y.; Sha J.; Zhong C.; Hu W.; Zhao N. Preparation of MoS2/TiO2 based nanocomposites for photocatalysis and rechargeable batteries: Progress, challenges, and perspective. Nanoscale 2018, 10, 34–68. 10.1039/C7NR07366F. 29211094

[ref22] Wang R.; Liu H.; Zhang Y.; Sun K.; Bao W. Integrated Photovoltaic Charging and Energy Storage Systems: Mechanism, Optimization, and Future. Small 2022, 18, 2203014 10.1002/smll.202203014. 35780491

[ref23] Azevedo J.; Seipp T.; Burfeind J.; Sousa C.; Bentien A.; Araújo J. P.; Mendes A. Unbiased solar energy storage: Photoelectrochemical redox flow battery. Nano Energy 2016, 22, 396–405. 10.1016/j.nanoen.2016.02.029.

[ref24] Liao S.; Zong X.; Seger B.; Pedersen T.; Yao T.; Ding C.; Shi J.; Chen J.; Li C. Integrating a dual-silicon photoelectrochemical cell into a redox flow battery for unassisted photocharging. Nat. Commun. 2016, 7. 10.1038/ncomms11474 PMC4857481 27142885

[ref25] Urbain F.; Murcia-López S.; Nembhard N.; Vázquez-Galván J.; Flox C.; Smirnov V.; Welter K.; Andreu T.; Finger F.; Morante J. R. Solar vanadium redox-flow battery powered by thin-film silicon photovoltaics for efficient photoelectrochemical energy storage. J. Phys. D: Appl. Phys. 2019, 52, 044001 10.1088/1361-6463/aaeab9.

[ref26] Li W.; Fu H.-C.; Li L.; Cabán-Acevedo M.; He J.-H.; Jin S. Integrated Photoelectrochemical Solar Energy Conversion and Organic Redox Flow Battery Devices. Angew. Chem. 2016, 128, 13298–13302. 10.1002/ange.201606986. 27654317

[ref27] Song Z.; et al. Photocapacitor integrating voltage-adjustable hybrid supercapacitor and silicon solar cell generating a Joule efficiency of 86. Energy Environmental Science 2022, 15, 4247–4258. 10.1039/D2EE01744J.

[ref28] Song Z.; Wu J.; Sun L.; Zhu T.; Deng C.; Wang X.; Li G.; Du Y.; Chen Q.; Sun W.; Fan L.; Chen H.; Lin J.; Lan Z. Photocapacitor integrating perovskite solar cell and symmetrical supercapacitor generating a conversion storage efficiency over 20. Nano Energy 2022, 100, 107501 10.1016/j.nanoen.2022.107501.

[ref29] Murakami T. N.; Kawashima N.; Miyasaka T. A high-voltage dye-sensitized photocapacitor of a three-electrode system. Chem. Commun. 2005, 3346 10.1039/b503122b. 15983669

[ref30] Zhang X.; Huang X.; Li C.; Jiang H. Dye-sensitized solar cell with energy storage function through PVDF/ZnO nanocomposite counter electrode. Adv. Mater. 2013, 25, 4093–4096. 10.1002/adma.201301088. 23740719 PMC3796954

[ref31] Bagheri N.; Aghaei A.; Ghotbi M. Y.; Marzbanrad E.; Vlachopoulos N.; Häggman L.; Wang M.; Boschloo G.; Hagfeldt A.; Skunik-Nuckowska M.; Kulesza P. J. Combination of asymmetric supercapacitor utilizing activated carbon and nickel oxide with cobalt polypyridyl-based dye-sensitized solar cell. Electrochim. Acta 2014, 143, 390–397. 10.1016/j.electacta.2014.07.125.

[ref32] Cohn A. P.; Erwin W. R.; Share K.; Oakes L.; Westover A. S.; Carter R. E.; Bardhan R.; Pint C. L. All Silicon Electrode Photocapacitor for Integrated Energy Storage and Conversion. Nano Lett. 2015, 15, 2727–2731. 10.1021/acs.nanolett.5b00563. 25806838

[ref33] Ng C.; Lim H.; Hayase S.; Harrison I.; Pandikumar A.; Huang N. Potential active materials for photo-supercapacitor: A review. J. Power Sources 2015, 296, 169–185. 10.1016/j.jpowsour.2015.07.006.

[ref34] Liu R.; Liu Y.; Zou H.; Song T.; Sun B. Integrated solar capacitors for energy conversion and storage. Nano Res. 2017, 10, 1545–1559. 10.1007/s12274-017-1450-5.

[ref35] Fu Y.; Wu H.; Ye S.; Cai X.; Yu X.; Hou S.; Kafafy H.; Zou D. Integrated power fiber for energy conversion and storage. Energy Environ. Sci. 2013, 6, 805–812. 10.1039/c3ee23970e.

[ref36] Sun R.; Qin Z.; Li Z.; Fan H.; Lu S. Binary zinc-cobalt metal-organic framework derived mesoporous ZnCo2O4@NC polyhedron as a high-performance lithium-ion battery anode. Dalton Transactions 2020, 49, 14237–14242. 10.1039/D0DT03132A. 33026024

[ref37] Yu M.; Ren X.; Ma L.; Wu Y. Integrating a redox-coupled dye-sensitized photoelectrode into a lithium-oxygen battery for photoassisted charging. Nat. Commun. 2014, 5, 1–6. 10.1038/ncomms6111. 25277368

[ref38] Miyasaka T.; Murakami T. N. The photocapacitor: An efficient self-charging capacitor for direct storage of solar energy. Appl. Phys. Lett. 2004, 85, 3932–3934. 10.1063/1.1810630.

[ref39] Gao K.; Ti D.; Zhang Z. A photocapacitor with high working voltage and energy density. Sustainable Energy Fuels 2019, 3, 1937–1942. 10.1039/C9SE00325H.

[ref40] Jin W.; Ovhal M. M.; Lee H. B.; Tyagi B.; Kang J. Scalable, All-Printed Photocapacitor Fibers and Modules based on Metal-Embedded Flexible Transparent Conductive Electrodes for Self-Charging Wearable Applications. Adv. Energy Mater. 2021, 11, 2003509 10.1002/aenm.202003509.

[ref41] Melikov R.; Srivastava S. B.; Karatum O.; Dogru-Yuksel I. B.; Bahmani Jalali H.; Sadeghi S.; Dikbas U. M.; Ulgut B.; Kavakli I. H.; Cetin A. E.; Nizamoglu S. Plasmon-Coupled Photocapacitor Neuromodulators. ACS Appl. Mater. Interfaces 2020, 12, 35940–35949. 10.1021/acsami.0c09455. 32667186 PMC7598729

[ref42] Minnaert B.; Veelaert P. A proposal for typical artificial light sources for the characterization of indoor photovoltaic applications. Energies 2014, 7, 1500–1516. 10.3390/en7031500.

[ref43] Mubiru J.; Banda E. J. Estimation of monthly average daily global solar irradiation using artificial neural networks. Sol. Energy 2008, 82, 181–187. 10.1016/j.solener.2007.06.003.

[ref44] Sarwar J.; Georgakis G.; LaChance R.; Ozalp N. Description and characterization of an adjustable flux solar simulator for solar thermal, thermochemical and photovoltaic applications. Sol. Energy 2014, 100, 179–194. 10.1016/j.solener.2013.12.008.

[ref45] Michaels H.; Benesperi I.; Freitag M. Challenges and prospects of ambient hybrid solar cell applications. Chemical Science 2021, 12, 5002–5015. 10.1039/D0SC06477G. 34168767 PMC8179625

[ref46] Tan Y. K.; Panda S. K. Energy harvesting from hybrid indoor ambient light and thermal energy sources for enhanced performance of wireless sensor nodes. IEEE Transactions on Industrial Electronics 2011, 58, 4424–4435. 10.1109/TIE.2010.2102321.

[ref47] Kurnik J.; Jankovec M.; Brecl K.; Topic M. Outdoor testing of PV module temperature and performance under different mounting and operational conditions. Sol. Energy Mater. Sol. Cells 2011, 95, 373–376. 10.1016/j.solmat.2010.04.022.

[ref48] Liu C.; Yu Z.; Neff D.; Zhamu A.; Jang B. Z. Graphene-based supercapacitor with an ultrahigh energy density. Nano Lett. 2010, 10, 4863–4868. 10.1021/nl102661q. 21058713

[ref49] Zhang L.; Zhao X. S. Carbon-based materials as supercapacitor electrodes. Chem. Soc. Rev. 2009, 38, 2520–2531. 10.1039/b813846j. 19690733

[ref50] Yu Z.; Tetard L.; Zhai L.; Thomas J. Supercapacitor electrode materials: Nanostructures from 0 to 3 dimensions. Energy Environ. Sci. 2015, 8, 702–730. 10.1039/C4EE03229B.

[ref51] Naoi K.; Naoi W.; Aoyagi S.; Miyamoto J. I.; Kamino T. New generation ”nanohybrid supercapacitor. Acc. Chem. Res. 2013, 46, 1075–1083. 10.1021/ar200308h. 22433167

[ref52] Iro Z. S.; Subramani C.; Dash S. S. A brief review on electrode materials for supercapacitor. Int. J. Electrochem. Sci. 2016, 11, 10628–10643. 10.20964/2016.12.50.

[ref53] NREL. Best Research-Cell Efficiency Chart. NREL, 2022; https://www.nrel.gov/pv/cell-efficiency.html.

[ref54] Nelson J. A. The Physics Of Solar Cells; World Scientific Publishing Company, 2003.

[ref55] Chopra K. L.; Paulson P. D.; Dutta V. Thin-film solar cells: An overview. Progress in Photovoltaics: Research and Applications 2004, 12, 69–92. 10.1002/pip.541.

[ref56] Kaliyannan G. V.; Gunasekaran R.; Sivaraj S.; Jaganathan S.; Rathanasamy R. Fundamentals of Solar Cell Design; Wiley, 2021; pp 103–115.

[ref57] Chopra K. L.; Das S. R. Thin Film Solar Cells; Springer US: Boston, MA, 1983; pp 1–18.

[ref58] Sims L.; Egelhaaf H. J.; Hauch J. A.; Kogler F. R.; Steim R. Plastic solar cells. Comprehensive Renewable Energy 2012, 1, 439–480. 10.1016/B978-0-08-087872-0.00120-7.

[ref59] Wohrle B. D.; Meissner D. Organic solar cells. Springer Series in Materials Science 2014, 208, 67–214. 10.1007/978-3-319-10097-5_3.

[ref60] Kippelen B.; Brédas J. L. Organic photovoltaics. Energy Environ. Sci. 2009, 2, 251–261. 10.1039/b812502n.

[ref61] Mazzio K. A.; Luscombe C. K. The future of organic photovoltaics. Chem. Soc. Rev. 2015, 44, 78–90. 10.1039/C4CS00227J. 25198769

[ref62] Green M. A.; Ho-Baillie A.; Snaith H. J. The emergence of perovskite solar cells. Nat. Photonics 2014, 8, 506–514. 10.1038/nphoton.2014.134.

[ref63] Correa-Baena J. P.; Saliba M.; Buonassisi T.; Grätzel M.; Abate A.; Tress W.; Hagfeldt A. Promises and challenges of perovskite solar cells. Science 2017, 358, 739–744. 10.1126/science.aam6323. 29123060

[ref64] Zhou H.; Chen Q.; Li G.; Luo S.; Song T.-b.; Duan H.-S.; Hong Z.; You J.; Liu Y.; Yang Y. Interface engineering of highly efficient perovskite solar cells. Science 2014, 345, 542–546. 10.1126/science.1254050. 25082698

[ref65] Zeng P.; Deng W.; Liu M. Recent Advances of Device Components toward Efficient Flexible Perovskite Solar Cells. Solar RRL 2020, 4, 1900485 10.1002/solr.201900485.

[ref66] Hagfeldt A.; Boschloo G.; Sun L.; Kloo L.; Pettersson H. Dye-Sensitized Solar Cells. Chem. Rev. 2010, 110, 6595–6663. 10.1021/cr900356p. 20831177

[ref67] Benesperi I.; Michaels H.; Freitag M. The researcher’s guide to solid-state dye-sensitized solar cells. Journal of Materials Chemistry C 2018, 6, 11903–11942. 10.1039/C8TC03542C.

[ref68] Jiang R.; Michaels H.; Vlachopoulos N.; Freitag M. Dye-Sensitized Solar Cells: Mathematical Modelling, and Materials Design and Optimization; Elsevier Inc., 2019; pp 285–323.

[ref69] Banin U.; et al. Nanotechnology for catalysis and solar energy conversion. Nanotechnology 2021, 32, 042003 10.1088/1361-6528/abbce8. 33155576

[ref70] Freitag M.; Teuscher J.; Saygili Y.; Zhang X.; Giordano F.; Liska P.; Hua J.; Zakeeruddin S. M.; Moser J.-E.; Grätzel M.; Hagfeldt A. Dye-sensitized solar cells for efficient power generation under ambient lighting. Nat. Photonics 2017, 11, 372–378. 10.1038/nphoton.2017.60.

[ref71] Freitag M.; Teuscher J.; Saygili Y.; Zhang X.; Giordano F.; Liska P.; Hua J.; Zakeeruddin S. M.; Moser J. E.; Grätzel M.; Hagfeldt A. Dye-sensitized solar cells for efficient power generation under ambient lighting. Nat. Photonics 2017, 11, 372–378. 10.1038/nphoton.2017.60.

[ref72] Kamat P. V. Quantum dot solar cells. The next big thing in photovoltaics. J. Phys. Chem. Lett. 2013, 4, 908–918. 10.1021/jz400052e. 26291355

[ref73] Raffaelle R. P.; Castro S. L.; Hepp A. F.; Bailey S. G. Quantum dot solar cells. Progress in Photovoltaics: Research and Applications 2002, 10, 433–439. 10.1002/pip.452.

[ref74] Aroutiounian V.; Petrosyan S.; Khachatryan A.; Touryan K. Quantum dot solar cells. J. Appl. Phys. 2001, 89, 2268–2271. 10.1063/1.1339210.

[ref75] Carey G. H.; Abdelhady A. L.; Ning Z.; Thon S. M.; Bakr O. M.; Sargent E. H. Colloidal Quantum Dot Solar Cells. Chem. Rev. 2015, 115, 12732–12763. 10.1021/acs.chemrev.5b00063. 26106908

[ref76] Li J.; Qiao J.; Lian K. Hydroxide ion conducting polymer electrolytes and their applications in solid supercapacitors: A review. Energy Storage Materials 2020, 24, 6–21. 10.1016/j.ensm.2019.08.012.

[ref77] Hashmi S. A.; Latham R. J.; Linford R. G.; Schlindwein W. S. Conducting Polymer-based Electrochemical Redox Supercapacitors Using Proton and Lithium Ion Conducting Polymer Electrolytes. Polym. Int. 1998, 47, 28–33. 10.1002/(SICI)1097-0126(199809)47:1<28::AID-PI3>3.0.CO;2-C.

[ref78] Aravindan V.; Gnanaraj J.; Madhavi S.; Liu H. K. Lithium-ion conducting electrolyte salts for lithium batteries. Chem. Eur. J. 2011, 17, 14326–14346. 10.1002/chem.201101486. 22114046

[ref79] Kumar D.; Hashmi S. A. Ionic liquid based sodium ion conducting gel polymer electrolytes. Solid State Ionics 2010, 181, 416–423. 10.1016/j.ssi.2010.01.025.

[ref80] Cheour R.; Khriji S.; Abid M.; Kanoun O. Microcontrollers for IoT: Optimizations, Computing Paradigms, and Future Directions. Proceedings of the IEEE 6th World Forum on Internet of Things (WF-IoT); IEEE: New York, 2020; pp 1–7.

[ref81] Ojo M. O.; Giordano S.; Procissi G.; Seitanidis I. N. A Review of Low-End, Middle-End, and High-End Iot Devices. IEEE Access 2018, 6, 70528–70554. 10.1109/ACCESS.2018.2879615.

[ref82] Ibro M.; Marinova G. Review on Low-Power Consumption Techniques for FPGA-based designs in IoT technology. Proceedings of the 16th International Conference on Telecommunications, ConTEL 2021; IEEE: New York, 2021; pp 110–114.

[ref83] Cvitić I.; Peraković D.; Periša M.; Gupta B. Ensemble machine learning approach for classification of IoT devices in smart home. Int. J. Mach. Learn. Cybernetics 2021, 12, 3179 10.1007/s13042-020-01241-0.

[ref84] Vyas D. A.; Bhatt D.; Jha D. IoT: Trends, Challenges and Future Scope. Int. J. Comp. Sci. Commun. 2016, 7, 186–197.

[ref85] Jan M. A.; Khan F.; Alam M. In Recent Trends and Advances in Wireless and IoT-enabled Networks; Jan M. A., Khan F., Alam M., Eds.; EAI/Springer Innovations in Communication and Computing; Springer International Publishing: Cham, 2019.

[ref86] Gubbi J.; Buyya R.; Marusic S.; Palaniswami M. Internet of Things (IoT): A vision, architectural elements, and future directions. Future Generation Computer Systems 2013, 29, 1645–1660. 10.1016/j.future.2013.01.010.

[ref87] Mohammadi M.; Al-Fuqaha A. Enabling Cognitive Smart Cities Using Big Data and Machine Learning: Approaches and Challenges. IEEE Communications Magazine 2018, 56, 94–101. 10.1109/MCOM.2018.1700298.

[ref88] Whitmore A.; Agarwal A.; Da Xu L. The Internet of Things—A survey of topics and trends. Information Syst. Front. 2015, 17, 261–274. 10.1007/s10796-014-9489-2.

[ref89] Liouane Z.; Lemlouma T.; Roose P.; Weis F.; Messaoud H. An improved extreme learning machine model for the prediction of human scenarios in smart homes. Applied Intelligence 2018, 48, 2017–2030. 10.1007/s10489-017-1062-5.

[ref90] IoTNews. IoT technology will save eight times the energy it consumes by 2030, new report shows. 2022; https://iottechnews.com/news/2021/apr/21/iot-technology-will-save-eight-times-the-energy-it-consumes-by-2030-new-report-shows/.

[ref91] Sharma H.; Haque A.; Jaffery Z. A. Solar energy harvesting wireless sensor network nodes: A survey. J. Renewable Sustainable Energy 2018, 10, 023704 10.1063/1.5006619.

[ref92] Antunez P. D.; Bishop D. M.; Luo Y.; Haight R. Efficient kesterite solar cells with high open-circuit voltage for applications in powering distributed devices. Nature Energy 2017, 2, 884–890. 10.1038/s41560-017-0028-5.

[ref93] Mathews I.; Kantareddy S. N.; Buonassisi T.; Peters I. M. Technology and {{Market Perspective}} for {{Indoor Photovoltaic Cells. Joule 2019, 3, 1415–1426. 10.1016/j.joule.2019.03.026.

[ref94] Michaels H.; Rinderle M.; Benesperi I.; Freitag R.; Gagliardi A.; Freitag M. Emerging indoor photovoltaics for self-powered and self-aware IoT towards sustainable energy management. Chem. Sci. 2023, 14, 5350 10.1039/D3SC00659J. 37234887 PMC10207895

[ref95] Kim T.; Song W.; Son D. Y.; Ono L. K.; Qi Y. Lithium-ion batteries: outlook on present, future, and hybridized technologies. Journal of Materials Chemistry A 2019, 7, 2942–2964. 10.1039/C8TA10513H.

[ref96] Shao Y.; El-Kady M. F.; Sun J.; Li Y.; Zhang Q.; Zhu M.; Wang H.; Dunn B.; Kaner R. B. Design and Mechanisms of Asymmetric Supercapacitors. Chem. Rev. 2018, 118, 9233–9280. 10.1021/acs.chemrev.8b00252. 30204424

[ref97] K N.; Rout C. S. Photo-powered integrated supercapacitors: a review on recent developments, challenges and future perspectives. J. Mater. Chem. A 2021, 9, 8248–8278. 10.1039/D1TA00444A.

[ref98] Jahandar M.; Kim S.; Lim D. C. Indoor Organic Photovoltaics for Self-Sustaining IoT Devices: Progress, Challenges and Practicalization. ChemSusChem 2021, 14, 3449–3474. 10.1002/cssc.202100981. 34056847 PMC8519124

[ref99] Michaels H.; Rinderle M.; Freitag R.; Benesperi I.; Edvinsson T.; Socher R.; Gagliardi A.; Freitag M. Dye-sensitized solar cells under ambient light powering machine learning: towards autonomous smart sensors for the internet of things. Chemical Science 2020, 11, 2895–2906. 10.1039/C9SC06145B. 34122790 PMC8157489

[ref100] Polyzoidis C.; Rogdakis K.; Kymakis E. Indoor Perovskite Photovoltaics for the Internet of Things—Challenges and Opportunities toward Market Uptake. Adv. Energy Mater. 2021, 11, 2101854 10.1002/aenm.202101854.

[ref101] Hou X.; Wang Y.; Lee H. K. H.; Datt R.; Uslar Miano N.; Yan D.; Li M.; Zhu F.; Hou B.; Tsoi W. C.; Li Z. Indoor application of emerging photovoltaics - Progress, challenges and perspectives. Journal of Materials Chemistry A 2020, 8, 21503–21525. 10.1039/D0TA06950G.

[ref102] Mathews I.; Kantareddy S. N.; Buonassisi T.; Peters I. M. Technology and Market Perspective for Indoor Photovoltaic Cells. Joule 2019, 3, 1415–1426. 10.1016/j.joule.2019.03.026.

[ref103] Jin W. Y.; Ovhal M. M.; Lee H. B.; Tyagi B.; Kang J. W. Scalable, All-Printed Photocapacitor Fibers and Modules based on Metal-Embedded Flexible Transparent Conductive Electrodes for Self-Charging Wearable Applications. Adv. Energy Mater. 2021, 11, 2003509 10.1002/aenm.202003509.

[ref104] Gao Z.; Bumgardner C.; Song N.; Zhang Y.; Li J.; Li X. Cotton-textile-enabled flexible self-sustaining power packs via roll-to-roll fabrication. Nat. Commun. 2016, 7, 1–12. 10.1038/ncomms11586. PMC4873971 27189776

[ref105] Wen Z.; Yeh M. H.; Guo H.; Wang J.; Zi Y.; Xu W.; Deng J.; Zhu L.; Wang X.; Hu C.; Zhu L.; Sun X.; Wang Z. L. Self-powered textile for Wearable electronics by hybridizing fiber-shaped nanogenerators, solar cells, and supercapacitors. Sci. Adv. 2016, 2. 10.1126/sciadv.1600097 PMC5091355 27819039

[ref106] Sun J.; Gao K.; Lin X.; Gao C.; Ti D.; Zhang Z. Laser-Assisted Fabrication of Microphotocapacitors with High Energy Density and Output Voltage. ACS Applied Materials Interfaces 2021, 13, 419–428. 10.1021/acsami.0c16677. 33386055

[ref107] Song W.; Bie Z.; Yan W.; Zhu J.; Ma W. Interfacial engineering of nanostructured photoanode in fiber dye-sensitized solar cells for self-charging power systems. EcoMat 2022, 4, 1–9. 10.1002/eom2.12177.

[ref108] Li C.; Islam M. M.; Moore J.; Sleppy J.; Morrison C.; Konstantinov K.; Dou S. X.; Renduchintala C.; Thomas J. Wearable energy-smart ribbons for synchronous energy harvest and storage. Nat. Commun. 2016, 7, 1–10. 10.1038/ncomms13319. PMC5114596 27834367

[ref109] Navarrete-Astorga E.; Solís-Cortés D.; Rodríguez-Moreno J.; Dalchiele E. A.; Schrebler R.; Martín F.; Ramos-Barrado J. R. A new concept of a transparent photocapacitor. Chem. Commun. 2018, 54, 10762–10765. 10.1039/C8CC06112B. 30198534

[ref110] Plebankiewicz I.; Bogdanowicz K. A.; Iwan A. Photo-Rechargeable Electric Energy Storage Systems Based on Silicon Solar Cells and Supercapacitor-Engineering Concept. Energies 2020, 13, 3867 10.3390/en13153867.

[ref111] Hariraman K.; Selvam J.; Alagirisamy M.; Sivaraju S. Asymmetrical Supercapacitor based solar powered automatic emergency light. Proceedings of the 8th International Conference on Smart Structures and Systems (ICSSS); IEEE: New York, 2022; pp 1–5.

[ref112] Ling H.; Wu J.; Su F.; Tian Y.; Jun Liu Y. High performance electrochromic supercapacitors powered by perovskite-solar-cell for real-time light energy flow control. Chemical Engineering Journal 2022, 430, 133082 10.1016/j.cej.2021.133082.

[ref113] Li C.; Cong S.; Tian Z.; Song Y.; Yu L.; Lu C.; Shao Y.; Li J.; Zou G.; Rümmeli M. H.; Dou S.; Sun J.; Liu Z. Flexible perovskite solar cell-driven photo-rechargeable lithium-ion capacitor for self-powered wearable strain sensors. Nano Energy 2019, 60, 247–256. 10.1016/j.nanoen.2019.03.061.

[ref114] Annapureddy V.; Palneedi H.; Hwang G. T.; Peddigari M.; Jeong D. Y.; Yoon W. H.; Kim K. H.; Ryu J. Magnetic energy harvesting with magnetoelectrics: An emerging technology for self-powered autonomous systems. Sustainable Energy and Fuels 2017, 1, 2039–2052. 10.1039/C7SE00403F.

[ref115] Lee J.-H.; Yang G.; Kim C.-H.; Mahajan R. L.; Lee S.-Y.; Park S.-J. Flexible solid-state hybrid supercapacitors for the internet of everything (IoE). Energy Environ. Sci. 2022, 15, 2233–2258. 10.1039/D1EE03567C.

[ref116] Hussain A. M.; Hussain M. M. CMOS-Technology-Enabled Flexible and Stretchable Electronics for Internet of Everything Applications. Adv. Mater. 2016, 28, 4219–4249. 10.1002/adma.201504236. 26607553

[ref117] Wang P.; Hu M.; Wang H.; Chen Z.; Feng Y.; Wang J.; Ling W.; Huang Y. The Evolution of Flexible Electronics: From Nature, Beyond Nature, and To Nature. Adv. Sci. 2020, 7, 2001116 10.1002/advs.202001116. PMC7578875 33101851

[ref118] De Rossi F.; Pontecorvo T.; Brown T. M. Characterization of photovoltaic devices for indoor light harvesting and customization of flexible dye solar cells to deliver superior efficiency under artificial lighting. Applied Energy 2015, 156, 413–422. 10.1016/j.apenergy.2015.07.031.

[ref119] Morgan Pattison P.; Hansen M.; Tsao J. Y. LED lighting efficacy: Status and directions. Comptes Rendus Physique 2018, 19, 134–145. 10.1016/j.crhy.2017.10.013.

[ref120] Zhang D.; Stojanovic M.; Ren Y.; Cao Y.; Eickemeyer F. T.; Socie E.; Vlachopoulos N.; Moser J.-E.; Zakeeruddin S. M.; Hagfeldt A.; Grätzel M. A molecular photosensitizer achieves a Voc of 1.24{\thinspace}V enabling highly efficient and stable dye-sensitized solar cells with copper(II/I)-based electrolyte. Nat. Commun. 2021, 12, 1777 10.1038/s41467-021-21945-3. 33741953 PMC7979847

[ref121] Haight R.; Haensch W.; Friedman D. Solar-powering the Internet of Things. Science 2016, 353, 124–125. 10.1126/science.aag0476. 27387939

[ref122] Hassan Q.; Jaszczur M.; Abdulateef A. M.; Abdulateef J.; Hasan A.; Mohamad A. An analysis of photovoltaic/supercapacitor energy system for improving self-consumption and self-sufficiency. Energy Reports 2022, 8, 680–695. 10.1016/j.egyr.2021.12.021.

[ref123] Sun Y.; Liu T.; Kan Y.; Gao K.; Tang B.; Li Y. Flexible Organic Solar Cells: Progress and Challenges. Small Science 2021, 1, 2100001 10.1002/smsc.202100001. PMC11935921 40212042

[ref124] Fukuda K.; Yu K.; Someya T. The Future of Flexible Organic Solar Cells. Adv. Energy Mater. 2020, 10, 2000765 10.1002/aenm.202000765.

[ref125] Li G.; Sheng L.; Li T.; Hu J.; Li P.; Wang K. Engineering flexible dye-sensitized solar cells for portable electronics. Sol. Energy 2019, 177, 80–98. 10.1016/j.solener.2018.11.017.

[ref126] Yang L.; Feng J.; Liu Z.; Duan Y.; Zhan S.; Yang S.; He K.; Li Y.; Zhou Y.; Yuan N.; Ding J.; Liu S. Record-Efficiency Flexible Perovskite Solar Cells Enabled by Multifunctional Organic Ions Interface Passivation. Adv. Mater. 2022, 34, 2201681 10.1002/adma.202201681. 35435279

[ref127] Meng X.; Zhang L.; Xie Y.; Hu X.; Xing Z.; Huang Z.; Liu C.; Tan L.; Zhou W.; Sun Y.; Ma W.; Chen Y. A General Approach for Lab-to-Manufacturing Translation on Flexible Organic Solar Cells. Adv. Mater. 2019, 31, 1970294 10.1002/adma.201970294. 31423693

[ref128] Huang C.; Zhang J.; Young N. P.; Snaith H. J.; Grant P. S. Solid-state supercapacitors with rationally designed heterogeneous electrodes fabricated by large area spray processing for wearable energy storage applications. Sci. Rep. 2016, 6, 1–15. 10.1038/srep25684. 27161379 PMC4861981

[ref129] Browne M. P.; Redondo E.; Pumera M. 3D Printing for Electrochemical Energy Applications. Chem. Rev. 2020, 120, 2783–2810. 10.1021/acs.chemrev.9b00783. 32049499

[ref130] Areir M.; Xu Y.; Zhang R.; Harrison D.; Fyson J.; Pei E. A study of 3D printed active carbon electrode for the manufacture of electric double-layer capacitors. Journal of Manufacturing Processes 2017, 25, 351–356. 10.1016/j.jmapro.2016.12.020.

[ref131] Benzigar M. R.; Dasireddy V. D.; Guan X.; Wu T.; Liu G. Advances on Emerging Materials for Flexible Supercapacitors: Current Trends and Beyond. Adv. Funct. Mater. 2020, 30, 2002993 10.1002/adfm.202002993.

[ref132] Zhao Z.; Xia K.; Hou Y.; Zhang Q.; Ye Z.; Lu J. Designing flexible, smart and self-sustainable supercapacitors for portable/wearable electronics: From conductive polymers. Chem. Soc. Rev. 2021, 50, 12702–12743. 10.1039/D1CS00800E. 34643198

[ref133] Xu Y.; Lin Z.; Wei W.; Hao Y.; Liu S.; Ouyang J.; Chang J. Recent Progress of Electrode Materials for Flexible Perovskite Solar Cells. Nano-Micro Lett. 2022, 14, 1–30. 10.1007/s40820-022-00859-9. PMC9056588 35488940

[ref134] Xu Y.; Lin Z.; Zhang J.; Hao Y.; Ouyang J.; Liu S.; Chang J. Flexible perovskite solar cells: Material selection and structure design. Appl. Phys. Rev. 2022, 9, 021307 10.1063/5.0084596.

[ref135] Brunetti F.; Operamolla A.; Castro-Hermosa S.; Lucarelli G.; Manca V.; Farinola G. M.; Brown T. M. Printed Solar Cells and Energy Storage Devices on Paper Substrates. Adv. Funct. Mater. 2019, 29, 1806798 10.1002/adfm.201806798.

[ref136] Yun M. J.; Cha S. I.; Seo S. H.; Lee D. Y. Highly flexible dye-sensitized solar cells produced by sewing textile electrodes on cloth. Sci. Rep. 2014, 4, 1–6. 10.1038/srep05322. PMC4067621 24957920

[ref137] Wang X.; Zhao B.; Kan W.; Xie Y.; Pan K. Review on Low-Cost Counter Electrode Materials for Dye-Sensitized Solar Cells: Effective Strategy to Improve Photovoltaic Performance. Adv. Mater. Interfaces 2022, 9, 2101229 10.1002/admi.202101229.

[ref138] Murali B.; Gireesh Baiju K.; Krishna Prasad R.; Jayanarayanan K.; Kumaresan D. Fabrication of high-efficiency PET polymer-based flexible dye-sensitized solar cells and tapes via heat sink-supported thermal sintering of bilayer TiO2 photoanodes. Sustainable Energy and Fuels 2022, 6, 2503–2513. 10.1039/D2SE00111J.

[ref139] Wu C.; Chen B.; Zheng X.; Priya S. Scaling of the flexible dye sensitized solar cell module. Sol. Energy Mater. Sol. Cells 2016, 157, 438–446. 10.1016/j.solmat.2016.07.021.

[ref140] Sánchez S.; Vallés-Pelarda M.; Alberola-Borràs J. A.; Vidal R.; Jerónimo-Rendón J. J.; Saliba M.; Boix P. P.; Mora-Seró I. Flash infrared annealing as a cost-effective and low environmental impact processing method for planar perovskite solar cells. Mater. Today 2019, 31, 39–46. 10.1016/j.mattod.2019.04.021.

[ref141] Jin Y.; Li Z.; Qin L.; Liu X.; Mao L.; Wang Y.; Qin F.; Liu Y.; Zhou Y.; Zhang F. Laminated Free Standing PEDOT:PSS Electrode for Solution Processed Integrated Photocapacitors via Hydrogen-Bond Interaction. Advanced Materials Interfaces 2017, 4, 1700704 10.1002/admi.201700704.

[ref142] Devadiga D.; Selvakumar M.; Shetty P.; Santosh M. S. The integration of flexible dye-sensitized solar cells and storage devices towards wearable self-charging power systems: A review. Renewable and Sustainable Energy Reviews 2022, 159, 112252 10.1016/j.rser.2022.112252.

[ref143] Chen X.; Sun H.; Yang Z.; Guan G.; Zhang Z.; Qiu L.; Peng H. A novel ”energy fiber” by coaxially integrating dye-sensitized solar cell and electrochemical capacitor. Journal of Materials Chemistry A 2014, 2, 1897–1902. 10.1039/C3TA13712K.

[ref144] Liu K.; Chen Z.; Lv T.; Yao Y.; Li N.; Li H.; Chen T. A Self-supported Graphene/Carbon Nanotube Hollow Fiber for Integrated Energy Conversion and Storage. Nano-Micro Lett. 2020, 12, 1–11. 10.1007/s40820-020-0390-x. PMC7770695 34138272

[ref145] Liu R.; Takakuwa M.; Li A.; Inoue D.; Hashizume D.; Yu K.; Umezu S.; Fukuda K.; Someya T. Supercapacitors: An Efficient Ultra-Flexible Photo-Charging System Integrating Organic Photovoltaics and Supercapacitors. Adv. Energy Mater. 2020, 10, 2070090 10.1002/aenm.202070090.

[ref146] Yoshikawa K.; Kawasaki H.; Yoshida W.; Irie T.; Konishi K.; Nakano K.; Uto T.; Adachi D.; Kanematsu M.; Uzu H.; Yamamoto K. Silicon heterojunction solar cell with interdigitated back contacts for a photoconversion efficiency over 26. Nat. Energy 2017, 2, 17032 10.1038/nenergy.2017.32.

[ref147] Reich N. H.; van Sark W. G.; Turkenburg W. C. Charge yield potential of indoor-operated solar cells incorporated into Product Integrated Photovoltaic (PIPV). Renewable Energy 2011, 36, 642–647. 10.1016/j.renene.2010.07.018.

[ref148] Pecunia V.; Occhipinti L. G.; Hoye R. L. Emerging Indoor Photovoltaic Technologies for Sustainable Internet of Things. Adv. Energy Mater. 2021, 11, 2100698 10.1002/aenm.202100698.

[ref149] Green M. A.; Dunlop E. D.; Hohl-Ebinger J.; Yoshita M.; Kopidakis N.; Bothe K.; Hinken D.; Rauer M.; Hao X. Solar cell efficiency tables (Version 60). Progress in Photovoltaics: Research and Applications 2022, 30, 687–701. 10.1002/pip.3595.

[ref150] Tang C. W. Two-layer organic photovoltaic cell. Appl. Phys. Lett. 1986, 48, 183–185. 10.1063/1.96937.

[ref151] Hiramoto M.; Fujiwara H.; Yokoyama M. Three-layered organic solar cell with a photoactive interlayer of codeposited pigments. Appl. Phys. Lett. 1991, 58, 1062–1064. 10.1063/1.104423.

[ref152] Riede M.; Spoltore D.; Leo K. Organic Solar Cells—The Path to Commercial Success. Adv. Energy Mater. 2021, 11, 2002653 10.1002/aenm.202002653.

[ref153] Di Carlo Rasi D.; Janssen R. A. J. Advances in Solution-Processed Multijunction Organic Solar Cells. Adv. Mater. 2019, 31, 1806499 10.1002/adma.201806499. 30589124

[ref154] Tong Y.; et al. Progress of the key materials for organic solar cells. Science China Chemistry 2020, 63, 758–765. 10.1007/s11426-020-9726-0.

[ref155] Zhang G.; Zhao J.; Chow P. C. Y.; Jiang K.; Zhang J.; Zhu Z.; Zhang J.; Huang F.; Yan H. Nonfullerene Acceptor Molecules for Bulk Heterojunction Organic Solar Cells. Chem. Rev. 2018, 118, 3447–3507. 10.1021/acs.chemrev.7b00535. 29557657

[ref156] Bai F.; Zhang J.; Zeng A.; Zhao H.; Duan K.; Yu H.; Cheng K.; Chai G.; Chen Y.; Liang J.; Ma W.; Yan H. A highly crystalline non-fullerene acceptor enabling efficient indoor organic photovoltaics with high EQE and fill factor. Joule 2021, 5, 1231–1245. 10.1016/j.joule.2021.03.020.

[ref157] Ansari M. I. H.; Qurashi A.; Nazeeruddin M. K. Frontiers, opportunities, and challenges in perovskite solar cells: A critical review. Journal of Photochemistry and Photobiology C: Photochemistry Reviews 2018, 35, 1–24. 10.1016/j.jphotochemrev.2017.11.002.

[ref158] Kim J. Y.; Lee J. W.; Jung H. S.; Shin H.; Park N. G. High-Efficiency Perovskite Solar Cells. Chem. Rev. 2020, 120, 7867–7918. 10.1021/acs.chemrev.0c00107. 32786671

[ref159] Correa-Baena J. P.; Saliba M.; Buonassisi T.; Grätzel M.; Abate A.; Tress W.; Hagfeldt A. Promises and challenges of perovskite solar cells. Science 2017, 358, 739–744. 10.1126/science.aam6323. 29123060

[ref160] Dong C.; Li X.; Ma C.; Yang W.; Cao J.; Igbari F.; Wang Z.; Liao L. Lycopene-Based Bionic Membrane for Stable Perovskite Photovoltaics. Adv. Funct. Mater. 2021, 31, 2011242 10.1002/adfm.202011242.

[ref161] Li D.; Zhang D.; Lim K. S.; Hu Y.; Rong Y.; Mei A.; Park N. G.; Han H. A Review on Scaling Up Perovskite Solar Cells. Adv. Funct. Mater. 2021, 31, 2008621 10.1002/adfm.202008621.

[ref162] Dai T.; Cao Q.; Yang L.; Aldamasy M. H.; Li M.; Liang Q.; Lu H.; Dong Y.; Yang Y. Strategies for high-performance large-area perovskite solar cells toward commercialization. Crystals 2021, 11, 295 10.3390/cryst11030295.

[ref163] Reddy S. H.; Di Giacomo F.; Di Carlo A. Low-Temperature-Processed Stable Perovskite Solar Cells and Modules: A Comprehensive Review. Adv. Energy Mater. 2022, 12, 2103534 10.1002/aenm.202103534.

[ref164] Morales-Acevedo A.; Miyasaka T.; Muradami T. N. Comment on ”the photocapacitor: An efficient self-charging capacitor for direct storage of solar energy” [Appl. Phys. Lett. 85, 3932 (2004)]. Appl. Phys. Lett. 2005, 86, 196101 10.1063/1.1922589.

[ref165] Fakharuddin A.; Jose R.; Brown T. M.; Fabregat-Santiago F.; Bisquert J. A perspective on the production of dye-sensitized solar modules. Energy Environ. Sci. 2014, 7, 3952–3981. 10.1039/C4EE01724B.

[ref166] Kokkonen M.; Talebi P.; Zhou J.; Asgari S.; Soomro S. A.; Elsehrawy F.; Halme J.; Ahmad S.; Hagfeldt A.; Hashmi S. G. Advanced research trends in dye-sensitized solar cells. Journal of Materials Chemistry A 2021, 9, 10527–10545. 10.1039/D1TA00690H. 33996094 PMC8095349

[ref167] Ren Y.; Zhang D.; Suo J.; Cao Y.; Eickemeyer F. T.; Vlachopoulos N.; Zakeeruddin S. M.; Hagfeldt A.; Grätzel M. Hydroxamic acid preadsorption raises efficiency of cosensitized solar cells. Nature 2023, 613, 60 10.1038/s41586-022-05460-z. 36288749

[ref168] De Rossi F.; Pontecorvo T.; Brown T. M. Characterization of photovoltaic devices for indoor light harvesting and customization of flexible dye solar cells to deliver superior efficiency under artificial lighting. Applied Energy 2015, 156, 413–422. 10.1016/j.apenergy.2015.07.031.

[ref169] Cao Y.; Liu Y.; Zakeeruddin S. M.; Hagfeldt A.; Grätzel M. Direct Contact of Selective Charge Extraction Layers Enables High-Efficiency Molecular Photovoltaics. Joule 2018, 2, 1108–1117. 10.1016/j.joule.2018.03.017.

[ref170] Jilakian M.; Ghaddar T. H. Eco-Friendly Aqueous Dye-Sensitized Solar Cell with a Copper(I/II) Electrolyte System: Efficient Performance under Ambient Light Conditions. ACS Applied Energy Materials 2022, 5, 257–265. 10.1021/acsaem.1c02789.

[ref171] Devadiga D.; Selvakumar M.; Shetty P.; Santosh M. S. Dye-Sensitized Solar Cell for Indoor Applications: A Mini-Review. J. Electron. Mater. 2021, 50, 3187–3206. 10.1007/s11664-021-08854-3.

[ref172] Pan Z.; Rao H.; Mora-Seró I.; Bisquert J.; Zhong X. Quantum dot-sensitized solar cells. Chem. Soc. Rev. 2018, 47, 7659–7702. 10.1039/C8CS00431E. 30209490

[ref173] Kouhnavard M.; Ikeda S.; Ludin N. A.; Ahmad Khairudin N. B.; Ghaffari B. V.; Mat-Teridi M. A.; Ibrahim M. A.; Sepeai S.; Sopian K. A review of semiconductor materials as sensitizers for quantum dot-sensitized solar cells. Renewable and Sustainable Energy Reviews 2014, 37, 397–407. 10.1016/j.rser.2014.05.023.

[ref174] Mora-Seró I. Current Challenges in the Development of Quantum Dot Sensitized Solar Cells. Adv. Energy Mater. 2020, 10, 2001774 10.1002/aenm.202001774.

[ref175] Kumar P. N.; Das A.; Kolay A.; Deepa M. Simple strategies deployed for developing efficient and stable solution processed quantum dot solar cells. Materials Advances 2022, 3, 2249–2267. 10.1039/D1MA00851J.

[ref176] Singh S.; Khan Z. H.; Khan M. B.; Kumar P.; Kumar P. Quantum dots-sensitized solar cells: a review on strategic developments. Bull. Mater. Sci. 2022, 45. 10.1007/s12034-022-02662-z

[ref177] Matsui T.; Bidiville A.; Maejima K.; Sai H.; Koida T.; Suezaki T.; Matsumoto M.; Saito K.; Yoshida I.; Kondo M. High-efficiency amorphous silicon solar cells: Impact of deposition rate on metastability. Appl. Phys. Lett. 2015, 106, 053901 10.1063/1.4907001.

[ref178] Kayes B. M.; Nie H.; Twist R.; Spruytte S. G.; Reinhardt F.; Kizilyalli I. C.; Higashi G. S. 27.6 for single-junction solar cells under 1 sun illumination. 2011 37th IEEE Photovoltaic Specialists Conference; IEEE: New York, 2011; pp 000004–000008.

[ref179] Nakamura M.; Yamaguchi K.; Kimoto Y.; Yasaki Y.; Kato T.; Sugimoto H. Cd-Free Cu(In,Ga)(Se,S) 2 Thin-Film Solar Cell With Record Efficiency of 23.35. IEEE J. Photovoltaics 2019, 9, 1863–1867. 10.1109/JPHOTOV.2019.2937218.

[ref180] He C.; Bi Z.; Chen Z.; Guo J.; Xia X.; Lu X.; Min J.; Zhu H.; Ma W.; Zuo L.; Chen H. Compromising Charge Generation and Recombination with Asymmetric Molecule for High-Performance Binary Organic Photovoltaics with Over 18% Certified Efficiency. Adv. Funct. Mater. 2022, 32, 2112511 10.1002/adfm.202112511.

[ref181] Jeong M.; Choi I. W.; Go E. M.; Cho Y.; Kim M.; Lee B.; Jeong S.; Jo Y.; Choi H. W.; Lee J.; Bae J. H.; Kwak S. K.; Kim D. S.; Yang C. Stable perovskite solar cells with efficiency exceeding 24.8 voltage loss. Science 2020, 369, 1615–1620. 10.1126/science.abb7167. 32973026

[ref182] Winter M.; Brodd R. J. What Are Batteries, Fuel Cells, and Supercapacitors?. Chem. Rev. 2004, 104, 4245–4270. 10.1021/cr020730k. 15669155

[ref183] Wu J. Understanding the Electric Double-Layer Structure, Capacitance, and Charging Dynamics. Chem. Rev. 2022, 122, 10821–10859. 10.1021/acs.chemrev.2c00097. 35594506

[ref184] Simon P.; Gogotsi Y. Materials for electrochemical capacitors. Nat. Mater. 2008, 7, 845–854. 10.1038/nmat2297. 18956000

[ref185] Eftekhari A. The mechanism of ultrafast supercapacitors. Journal of Materials Chemistry A 2018, 6, 2866–2876. 10.1039/C7TA10013B.

[ref186] Simon P.; Gogotsi Y.; Dunn B. Where Do Batteries End and Supercapacitors Begin?. Science 2014, 343, 1210–1211. 10.1126/science.1249625. 24626920

[ref187] Huang S.; Zhu X.; Sarkar S.; Zhao Y. Challenges and opportunities for supercapacitors. APL Materials 2019, 7, 100901 10.1063/1.5116146.

[ref188] Liang K.; Tang X.; Hu W. High-performance three-dimensional nanoporous NiO film as a supercapacitor electrode. J. Mater. Chem. 2012, 22, 11062 10.1039/c2jm31526b.

[ref189] Huang K.-j.; Zhang J.-z.; Fan Y. One-step solvothermal synthesis of different morphologies CuS nanosheets compared as supercapacitor electrode materials. J. Alloys Compd. 2015, 625, 158–163. 10.1016/j.jallcom.2014.11.137.

[ref190] Rajesh M.; Raj C. J.; Manikandan R.; Kim B. C.; Park S. Y.; Yu K. H. A high performance PEDOT/PEDOT symmetric supercapacitor by facile in-situ hydrothermal polymerization of PEDOT nanostructures on fl exible carbon fi bre cloth electrodes. Mater. Today Energy 2017, 6, 96–104. 10.1016/j.mtener.2017.09.003.

[ref191] Huang M.; Zhang Y.; Li F.; Zhang L.; Ruoff R. S.; Wen Z.; Liu Q. Self-assembly of mesoporous nanotubes assembled from interwoven ultrathin birnessite-type MnO2 nanosheets for asymmetric supercapacitors. Sci. Rep. 2014, 4, 1–8. 10.1038/srep03878. PMC3902441 24464344

[ref192] Su F.; Miao M. Asymmetric carbon nanotube-MnO2 two-ply yarn supercapacitors for wearable electronics. Nanotechnology 2014, 25, 135401 10.1088/0957-4484/25/13/135401. 24583526

[ref193] Gao H.; Xiao F.; Ching C. B.; Duan H. Flexible All-Solid-State Asymmetric Supercapacitors Based on Free-Standing Carbon Nanotube/Graphene and Mn3O4 Nanoparticle/Graphene Paper Electrodes. ACS Appl. Mater. Interfaces 2012, 4, 7020–7026. 10.1021/am302280b. 23167563

[ref194] Choi B. G.; Chang S.-J.; Kang H.-W.; Park C. P.; Kim H. J.; Hong W. H.; Lee S.; Huh Y. S. High Performance of a Solid-State Flexible Asymmetric Supercapacitor Based on Graphene Films. Nanoscale 2012, 4, 4983–4988. 10.1039/c2nr30991b. 22751863

[ref195] Shen J.; Yang C.; Li X.; Wang G. High-Performance Asymmetric Supercapacitor Based on Nanoarchitectured Polyaniline/Graphene/Carbon Nanotube and Activated Graphene Electrodes. ACS Appl. Mater. Interfaces 2013, 5, 8467–8476. 10.1021/am4028235. 23931572

[ref196] Gao H.; Xiao F.; Ching C. B.; Duan H. High-Performance Asymmetric Supercapacitor Based on Graphene Hydrogel and Nanostructured MnO2. ACS Appl. Mater. Interfaces 2012, 4, 2801–2810. 10.1021/am300455d. 22545683

[ref197] Li Y.; Gao H.; Sun Z.; Li Q.; Xu Y.; Ge C.; Cao Y. Tuning Morphology and Conductivity in Two-Step Synthesis of Zinc-Cobalt Oxide and Sulfide Hybrid Nanoclusters as Highly-Performed Electrodes for Hybrid Supercapacitors. J. Solid State Electrochem 2018, 22, 3197–3207. 10.1007/s10008-018-4035-7.

[ref198] Yadav M. S.; Singh N.; Bobade S. M. Zinc Oxide Nanoparticles and Activated Charcoal-Based Nanocomposite Electrode for Supercapacitor Application. Ionics 2018, 24, 3611–3630. 10.1007/s11581-018-2527-1.

[ref199] Chee W. K.; Lim H. N.; Huang N. M. Electrochemical Properties of Free-Standing Polypyrrole/Graphene Oxide/Zinc Oxide Flexible Supercapacitor. Int. J. Energy Res. 2015, 39, 111–119. 10.1002/er.3225.

[ref200] Sui L.; Tang S.; Chen Y.; Dai Z.; Huangfu H.; Zhu Z.; Qin X.; Deng Y.; Haarberg G. M. An Asymmetric Supercapacitor with Good Electrochemical Performances Based on Ni(OH)2/AC/CNT and AC. Electrochim. Acta 2015, 182, 1159–1165. 10.1016/j.electacta.2015.09.111.

[ref201] Tang Q.; Wang W.; Wang G. The Perfect Matching between the Low-Cost Fe2O3 Nanowire Anode and the NiO Nanoflake Cathode Significantly Enhances the Energy Density of Asymmetric Supercapacitors. J. Mater. Chem. A 2015, 3, 6662–6670. 10.1039/C5TA00328H.

[ref202] Cho S.; Patil B.; Yu S.; Ahn S.; Hwang J.; Park C.; Do K.; Ahn H. Flexible, Swiss Roll, Fiber-Shaped, Asymmetric Supercapacitor Using MnO2 and Fe2O3 on Carbon Fibers. Electrochim. Acta 2018, 269, 499–508. 10.1016/j.electacta.2018.03.020.

[ref203] Nagamuthu S.; Ryu K.-S. MOF-derived Microstructural Interconnected Network Porous Mn2O3/C as Negative Electrode Material for Asymmetric Supercapacitor Device. CrystEngComm 2019, 21, 1442–1451. 10.1039/C8CE01683F.

[ref204] Luo J.; Wang J.; Liu S.; Wu W.; Jia T.; Yang Z.; Mu S.; Huang Y. Graphene Quantum Dots Encapsulated Tremella-like NiCo2O4 for Advanced Asymmetric Supercapacitors. Carbon 2019, 146, 1–8. 10.1016/j.carbon.2019.01.078.

[ref205] Kong D.; Ren W.; Cheng C.; Wang Y.; Huang Z.; Yang H. Y. Three-Dimensional NiCo2O4@Polypyrrole Coaxial Nanowire Arrays on Carbon Textiles for High-Performance Flexible Asymmetric Solid-State Supercapacitor. ACS Appl. Mater. Interfaces 2015, 7, 21334–21346. 10.1021/acsami.5b05908. 26372533

[ref206] Zhang J.; Li C.; Fan M.; Ma T.; Chen H.; Wang H. Two-Dimensional Nanosheets Constituted Trimetal Ni-Co-Mn Sulfide Nanoflower-like Structure for High-Performance Hybrid Supercapacitors. Appl. Surf. Sci. 2021, 565, 150482 10.1016/j.apsusc.2021.150482.

[ref207] Sun J.; Du X.; Wu R.; Mao H.; Xu C.; Chen H. Simple Synthesis of Honeysuckle-like CuCo2O4/CuO Composites as a Battery Type Electrode Material for High-Performance Hybrid Supercapacitors. Int. J. Hydrogen Energy 2021, 46, 66–79. 10.1016/j.ijhydene.2020.09.206.

[ref208] Subhadarshini S.; Pavitra E.; Rama Raju G. S.; Chodankar N. R.; Goswami D. K.; Han Y.-K.; Huh Y. S.; Das N. C. One-Dimensional NiSe–Se Hollow Nanotubular Architecture as a Binder-Free Cathode with Enhanced Redox Reactions for High-Performance Hybrid Supercapacitors. ACS Appl. Mater. Interfaces 2020, 12, 29302–29315. 10.1021/acsami.0c05612. 32525302

[ref209] Patil S. J.; Dubal D. P.; Lee D.-W. Gold Nanoparticles Decorated rGO-ZnCo2O4 Nanocomposite: A Promising Positive Electrode for High Performance Hybrid Supercapacitors. Chemical Engineering Journal 2020, 379, 122211 10.1016/j.cej.2019.122211.

[ref210] Qin W.; Liu Y.; Liu X.; Yang G. Facile and Scalable Production of Amorphous Nickel Borate for High Performance Hybrid Supercapacitors. J. Mater. Chem. A 2018, 6, 19689–19695. 10.1039/C8TA07385F.

[ref211] Ma Z.; Zheng R.; Liu Y.; Ying Y.; Shi W. Carbon Nanotubes Interpenetrating MOFs-derived Co-Ni-S Composite Spheres with Interconnected Architecture for High Performance Hybrid Supercapacitor. J. Colloid Interface Sci. 2021, 602, 627–635. 10.1016/j.jcis.2021.06.027. 34147753

[ref212] Fischer J.; Thümmler K.; Fischer S.; Gonzalez Martinez I. G.; Oswald S.; Mikhailova D. Activated Carbon Derived from Cellulose and Cellulose Acetate Microspheres as Electrode Materials for Symmetric Supercapacitors in Aqueous Electrolytes. Energy Fuels 2021, 35, 12653–12665. 10.1021/acs.energyfuels.1c01449.

[ref213] Wang G.; Zhang L.; Zhang J. A review of electrode materials for electrochemical supercapacitors. Chem. Soc. Rev. 2012, 41, 797–828. 10.1039/C1CS15060J. 21779609

[ref214] Dubey R.; Guruviah V. Review of carbon-based electrode materials for supercapacitor energy storage. Ionics 2019, 25, 1419–1445. 10.1007/s11581-019-02874-0.

[ref215] Liu C.; Yu Z.; Neff D.; Zhamu A.; Jang B. Z. Graphene-Based Supercapacitor with an Ultrahigh Energy Density. Nano Lett. 2010, 10, 4863–4868. 10.1021/nl102661q. 21058713

[ref216] Gund G. S.; Dubal D. P.; Shinde S. S.; Lokhande C. D. Architectured Morphologies of Chemically Prepared NiO/MWCNTs Nanohybrid Thin Films for High Performance Supercapacitors. ACS Applied Materials Interfaces 2014, 6, 3176–3188. 10.1021/am404422g. 24548054

[ref217] Fan Y.; Yang X.; Zhu B.; Liu P.-f.; Lu H.-t. Micro-mesoporous carbon spheres derived from carrageenan as electrode material for supercapacitors. J. Power Sources 2014, 268, 584–590. 10.1016/j.jpowsour.2014.06.100.

[ref218] Zang J.; Bao S.-J.; Li C. M.; Bian H.; Cui X.; Bao Q.; Sun C. Q.; Guo J.; Lian K. Well-Aligned Cone-Shaped Nanostructure of Polypyrrole/RuO 2 and Its Electrochemical Supercapacitor. J. Phys. Chem. C 2008, 112, 14843–14847. 10.1021/jp8049558.

[ref219] Yu G.; Hu L.; Vosgueritchian M.; Wang H.; Xie X.; McDonough J. R.; Cui X.; Cui Y.; Bao Z. Solution-Processed Graphene/MnO 2 Nanostructured Textiles for High-Performance Electrochemical Capacitors. Nano Lett. 2011, 11, 2905–2911. 10.1021/nl2013828. 21667923

[ref220] da Silveira Firmiano E. G.; Rabelo A. C.; Dalmaschio C. J.; Pinheiro A. N.; Pereira E. C.; Schreiner W. H.; Leite E. R. Supercapacitor Electrodes Obtained by Directly Bonding 2D MoS 2 on Reduced Graphene Oxide. Adv. Energy Mater. 2014, 4, 1301380 10.1002/aenm.201301380.

[ref221] Zhao J.; Wu J.; Li B.; Du W.; Huang Q.; Zheng M.; Xue H.; Pang H. Facile synthesis of polypyrrole nanowires for high-performance supercapacitor electrode materials. Progress in Natural Science: Materials International 2016, 26, 237–242. 10.1016/j.pnsc.2016.05.015.

[ref222] Huang Z.; Ji Z.; Feng Y.; Wang P.; Huang Y. Flexible and stretchable polyaniline supercapacitor with a high rate capability. Polym. Int. 2021, 70, 437–442. 10.1002/pi.5982.

[ref223] Österholm A. M.; Ponder J. F.; Kerszulis J. A.; Reynolds J. R. Solution Processed PEDOT Analogues in Electrochemical Supercapacitors. ACS Applied Materials Interfaces 2016, 8, 13492–13498. 10.1021/acsami.6b02434. 27195798

[ref224] Naoi K.; Simon P. New materials and new confgurations for advanced electrochemical capacitors. Electrochemical Society Interface 2008, 17, 34–37. 10.1149/2.F04081IF.

[ref225] Shao Y.; El-Kady M. F.; Sun J.; Li Y.; Zhang Q.; Zhu M.; Wang H.; Dunn B.; Kaner R. B. Design and Mechanisms of Asymmetric Supercapacitors. Chem. Rev. 2018, 118, 9233–9280. 10.1021/acs.chemrev.8b00252. 30204424

[ref226] Iqbal M. Z.; Faisal M. M.; Ali S. R. Integration of supercapacitors and batteries towards high-performance hybrid energy storage devices. International Journal of Energy Research 2021, 45, 1449–1479. 10.1002/er.5954.

[ref227] Yang Y.; Hoang M. T.; Bhardwaj A.; Wilhelm M.; Mathur S.; Wang H. Perovskite solar cells based self-charging power packs: Fundamentals, applications and challenges. Nano Energy 2022, 94, 106910 10.1016/j.nanoen.2021.106910.

[ref228] Muzaffar A.; Ahamed M. B.; Deshmukh K.; Thirumalai J. A review on recent advances in hybrid supercapacitors: Design, fabrication and applications. Renewable and Sustainable Energy Reviews 2019, 101, 123–145. 10.1016/j.rser.2018.10.026.

[ref229] Zhang M.; Fan H.; Gao Y.; Zhao N.; Wang C.; Ma J.; Ma L.; Yadav A. K.; Wang W.; Vincent Lee W. S.; Xiong T.; Xue J.; Xia Z. Preaddition of Cations to Electrolytes for Aqueous 2. 2 V High Voltage Hybrid Supercapacitor with Superlong Cycling Life and Its Energy Storage Mechanism. ACS Appl. Mater. Interfaces 2020, 12, 17659–17668. 10.1021/acsami.0c03908. 32202755

[ref230] Dubal D. P.; Chodankar N. R.; Kim D. H.; Gomez-Romero P. Towards flexible solid-state supercapacitors for smart and wearable electronics. Chem. Soc. Rev. 2018, 47, 2065–2129. 10.1039/C7CS00505A. 29399689

[ref231] Yang Y.; Zhu T.; Shen L.; Liu Y.; Zhang D.; Zheng B.; Gong K.; Zheng J.; Gong X. Recent progress in the all-solid-state flexible supercapacitors. SmartMat 2022, 3, 349–383. 10.1002/smm2.1103.

[ref232] Zhong C.; Deng Y.; Hu W.; Qiao J.; Zhang L.; Zhang J. A review of electrolyte materials and compositions for electrochemical supercapacitors. Chem. Soc. Rev. 2015, 44, 7484–7539. 10.1039/C5CS00303B. 26050756

[ref233] Chen S.; Qiu L.; Cheng H. M. Carbon-Based Fibers for Advanced Electrochemical Energy Storage Devices. Chem. Rev. 2020, 120, 2811–2878. 10.1021/acs.chemrev.9b00466. 32073258

[ref234] Jiang D. E.; Jin Z.; Henderson D.; Wu J. Solvent effect on the pore-size dependence of an organic electrolyte supercapacitor. J. Phys. Chem. Lett. 2012, 3, 1727–1731. 10.1021/jz3004624. 26291850

[ref235] Yang Y.; Zhu T.; Shen L.; Liu Y.; Zhang D.; Zheng B.; Gong K.; Zheng J.; Gong X. Recent progress in the all-solid-state flexible supercapacitors. SmartMat 2022, 3, 349–383. 10.1002/smm2.1103.

[ref236] Chen H.; Ling M.; Hencz L.; Ling H. Y.; Li G.; Lin Z.; Liu G.; Zhang S. Exploring Chemical, Mechanical, and Electrical Functionalities of Binders for Advanced Energy-Storage Devices. Chem. Rev. 2018, 118, 8936–8982. 10.1021/acs.chemrev.8b00241. 30133259

[ref237] Akin M.; Zhou X. Recent advances in solid-state supercapacitors: From emerging materials to advanced applications. International Journal of Energy Research 2022, 46, 10389–10452. 10.1002/er.7918.

[ref238] Deng L.; Wang J.; Zhu G.; Kang L.; Hao Z.; Lei Z.; Yang Z.; Liu Z. H. RuO2/graphene hybrid material for high performance electrochemical capacitor. J. Power Sources 2014, 248, 407–415. 10.1016/j.jpowsour.2013.09.081.

[ref239] Liu X.; Shang P.; Zhang Y.; Wang X.; Fan Z.; Wang B.; Zheng Y. Three-dimensional and stable polyaniline-grafted graphene hybrid materials for supercapacitor electrodes. Journal of Materials Chemistry A 2014, 2, 15273–15278. 10.1039/C4TA03077J.

[ref240] Sinha P.; Kar K. K.; Naskar A. K. A Flexible, Redox-Active, Aqueous Electrolyte-Based Asymmetric Supercapacitor with High Energy Density Based on Keratin-Derived Renewable Carbon. Adv. Mater. Technol. 2022, 7, 2200133 10.1002/admt.202200133.

[ref241] Yazar S.; Arvas M. B.; Sahin Y. An ultrahigh-energy density and wide potential window aqueous electrolyte supercapacitor built by polypyrrole/aniline 2-sulfonic acid modified carbon felt electrode. International Journal of Energy Research 2022, 46, 8042–8060. 10.1002/er.7706.

[ref242] Zhang S. W.; Yin B. S.; Liu X. X.; Gu D. M.; Gong H.; Wang Z. B. A high energy density aqueous hybrid supercapacitor with widened potential window through multi approaches. Nano Energy 2019, 59, 41–49. 10.1016/j.nanoen.2019.02.001.

[ref243] Wu X.; Huang B.; Wang Q.; Wang Y. Wide potential and high energy density for an asymmetric aqueous supercapacitor. Journal of Materials Chemistry A 2019, 7, 19017–19025. 10.1039/C9TA06428A.

[ref244] Gong X.; Xu H.; Zhang M.; Cheng X.; Wu Y.; Zhang H.; Yan H.; Dai Y.; Zheng J. C. 2.4 V high performance supercapacitors enabled by polymer-strengthened 3 m aqueous electrolyte. J. Power Sources 2021, 505, 230078 10.1016/j.jpowsour.2021.230078.

[ref245] Zhang D.; Yang B.; She W.; Gao S.; Wang J.; Wang Y.; Wang K.; Li H.; Han L. Simultaneously Achieving High Energy and Power Density for Ultrafast-Charging Supercapacitor Built by a Semi-Graphitic Hierarchical Porous Carbon Nanosheet and a High-Voltage Alkaline Aqueous Electrolyte. J. Power Sources 2021, 506, 230103 10.1016/j.jpowsour.2021.230103.

[ref246] Hou Y.; Chen L.; Liu P.; Kang J.; Fujita T.; Chen M. Nanoporous Metal Based Flexible Asymmetric Pseudocapacitors. J. Mater. Chem. A 2014, 2, 10910–10916. 10.1039/C4TA00969J.

[ref247] Bu X.; Su L.; Dou Q.; Lei S.; Yan X. A Low-Cost “Water-in-Salt” Electrolyte for a 2.3 V High-Rate Carbon-Based Supercapacitor. J. Mater. Chem. A 2019, 7, 7541–7547. 10.1039/C9TA00154A.

[ref248] Rogier C.; Pognon G.; Galindo C.; Nguyen G. T. M.; Vancaeyzeele C.; Aubert P. H. MoO3-Carbon Nanotube Negative Electrode Designed for a Fully Hybrid Asymmetric Metal Oxide-Based Pseudocapacitor Operating in an Organic Electrolyte. ACS Applied Energy Materials 2022, 5, 9361–9372. 10.1021/acsaem.2c00632.

[ref249] Brandt A.; Isken P.; Lex-Balducci A.; Balducci A. Adiponitrile-Based Electrochemical Double Layer Capacitor. J. Power Sources 2012, 204, 213–219. 10.1016/j.jpowsour.2011.12.025.

[ref250] Gandla D.; Zhang F.; Tan D. Q. Advantage of Larger Interlayer Spacing of a Mo2Ti2C3MXene Free-Standing Film Electrode toward an Excellent Performance Supercapacitor in a Binary Ionic Liquid-Organic Electrolyte. ACS Omega 2022, 7, 7190–7198. 10.1021/acsomega.1c06761. 35252709 PMC8892664

[ref251] Perricone E.; Chamas M.; Cointeaux L.; Leprêtre J. C.; Judeinstein P.; Azais P.; Béguin F.; Alloin F. Investigation of Methoxypropionitrile as Co-Solvent for Ethylene Carbonate Based Electrolyte in Supercapacitors. A Safe and Wide Temperature Range Electrolyte. Electrochim. Acta 2013, 93, 1–7. 10.1016/j.electacta.2013.01.084.

[ref252] Yu X.; Ruan D.; Wu C.; Wang J.; Shi Z. Spiro-(1,1’)-Bipyrrolidinium Tetrafluoroborate Salt as High Voltage Electrolyte for Electric Double Layer Capacitors. J. Power Sources 2014, 265, 309–316. 10.1016/j.jpowsour.2014.04.144.

[ref253] Jänes A.; Eskusson J.; Thomberg T.; Lust E. Supercapacitors Based on Propylene Carbonate with Small Addition of Different Sulfur Containing Organic Solvents. J. Electrochem. Soc. 2014, 161, A1284 10.1149/2.0691409jes.

[ref254] Qian W.; Sun F.; Xu Y.; Qiu L.; Liu C.; Wang S.; Yan F. Human Hair-Derived Carbon Flakes for Electrochemical Supercapacitors. Energy Environ. Sci. 2014, 7, 379–386. 10.1039/C3EE43111H.

[ref255] Väli R.; Laheäär A.; Jänes A.; Lust E. Characteristics of Non-Aqueous Quaternary Solvent Mixture and Na-salts Based Supercapacitor Electrolytes in a Wide Temperature Range. Electrochim. Acta 2014, 121, 294–300. 10.1016/j.electacta.2013.12.149.

[ref256] Park J.; Kim B.; Yoo Y.-E.; Chung H.; Kim W. Energy-Density Enhancement of Carbon-Nanotube-Based Supercapacitors with Redox Couple in Organic Electrolyte. ACS Appl. Mater. Interfaces 2014, 6, 19499–19503. 10.1021/am506258s. 25425124

[ref257] Li C.; Zhang X.; Wang K.; Sun X.; Liu G.; Li J.; Tian H.; Li J.; Ma Y. Scalable Self-Propagating High-Temperature Synthesis of Graphene for Supercapacitors with Superior Power Density and Cyclic Stability. Adv. Mater. 2017, 29, 1604690 10.1002/adma.201604690. 27943446

[ref258] Nguyen Q. D.; Patra J.; Hsieh C.-T.; Li J.; Dong Q.-F.; Chang J.-K. Supercapacitive Properties of Micropore- and Mesopore-Rich Activated Carbon in Ionic-Liquid Electrolytes with Various Constituent Ions. ChemSusChem 2018, 12, 449–456. 10.1002/cssc.201802489. 30548119

[ref259] Khan I. A.; Shah F. U. Fluorine-Free Ionic Liquid-Based Electrolyte for Supercapacitors Operating at Elevated Temperatures. ACS Sustainable Chem. Eng. 2020, 8, 10212–10221. 10.1021/acssuschemeng.0c02568.

[ref260] Wang X.; Zhou H.; Sheridan E.; Walmsley J. C.; Ren D.; Chen D. Geometrically Confined Favourable Ion Packing for High Gravimetric Capacitance in Carbon–Ionic Liquid Supercapacitors. Energy Environ. Sci. 2016, 9, 232–239. 10.1039/C5EE02702K.

[ref261] Wang X.; Mehandzhiyski A. Y.; Arstad B.; Van Aken K. L.; Mathis T. S.; Gallegos A.; Tian Z.; Ren D.; Sheridan E.; Grimes B. A.; Jiang D.-e.; Wu J.; Gogotsi Y.; Chen D. Selective Charging Behavior in an Ionic Mixture Electrolyte-Supercapacitor System for Higher Energy and Power. J. Am. Chem. Soc. 2017, 139, 18681–18687. 10.1021/jacs.7b10693. 29185334

[ref262] Navalpotro P.; Palma J.; Anderson M.; Marcilla R. High Performance Hybrid Supercapacitors by Using Para-Benzoquinone Ionic Liquid Redox Electrolyte. J. Power Sources 2016, 306, 711–717. 10.1016/j.jpowsour.2015.12.103.

[ref263] Sato T.; Masuda G.; Takagi K. Electrochemical Properties of Novel Ionic Liquids for Electric Double Layer Capacitor Applications. Electrochim. Acta 2004, 49, 3603–3611. 10.1016/j.electacta.2004.03.030.

[ref264] Hussain S.; Liu T.; Javed M. S.; Aslam N.; Shaheen N.; Zhao S.; Zeng W.; Wang J. Amaryllis-like NiCo2S4 Nanoflowers for High-Performance Flexible Carbon-Fiber-Based Solid-State Supercapacitor. Ceram. Int. 2016, 42, 11851–11857. 10.1016/j.ceramint.2016.04.107.

[ref265] Wang L.; Arif M.; Duan G.; Chen S.; Liu X. A High Performance Quasi-Solid-State Supercapacitor Based on CuMnO2 Nanoparticles. J. Power Sources 2017, 355, 53–61. 10.1016/j.jpowsour.2017.04.054.

[ref266] Vijayakumar V.; Anothumakkool B.; Torris A. T. A.; Nair S. B.; Badiger M. V.; Kurungot S. An All-Solid-State-Supercapacitor Possessing a Non-Aqueous Gel Polymer Electrolyte Prepared Using a UV-assisted in Situ Polymerization Strategy. J. Mater. Chem. A 2017, 5, 8461–8476. 10.1039/C7TA01514C.

[ref267] Miao F.; Shao C.; Li X.; Wang K.; Liu Y. Flexible Solid-State Supercapacitors Based on Freestanding Nitrogen-Doped Porous Carbon Nanofibers Derived from Electrospun Polyacrylonitrile@polyaniline Nanofibers. J. Mater. Chem. A 2016, 4, 4180–4187. 10.1039/C6TA00015K.

[ref268] Shao L.; Wang Q.; Ma Z.; Ji Z.; Wang X.; Song D.; Liu Y.; Wang N. A High-Capacitance Flexible Solid-State Supercapacitor Based on Polyaniline and Metal-Organic Framework (UiO-66) Composites. J. Power Sources 2018, 379, 350–361. 10.1016/j.jpowsour.2018.01.028.

[ref269] Kang D. A.; Kim K.; Karade S. S.; Kim H.; Hak Kim J. High-Performance Solid-State Bendable Supercapacitors Based on PEGBEM-g-PAEMA Graft Copolymer Electrolyte. Chemical Engineering Journal 2020, 384, 123308 10.1016/j.cej.2019.123308.

[ref270] Lu C.; Chen X. In Situ Synthesized PEO/NBR Composite Ionogels for High-Performance All-Solid-State Supercapacitors. Chem. Commun. 2019, 55, 8470–8473. 10.1039/C9CC03401C. 31265012

[ref271] Zhang X.; Kar M.; Mendes T. C.; Wu Y.; MacFarlane D. R. Supported Ionic Liquid Gel Membrane Electrolytes for Flexible Supercapacitors. Adv. Energy Mater. 2018, 8, 1702702 10.1002/aenm.201702702.

[ref272] Sahoo S.; Krishnamoorthy K.; Pazhamalai P.; Mariappan V. K.; Manoharan S.; Kim S.-J. High Performance Self-Charging Supercapacitors Using a Porous PVDF-ionic Liquid Electrolyte Sandwiched between Two-Dimensional Graphene Electrodes. J. Mater. Chem. A 2019, 7, 21693–21703. 10.1039/C9TA06245A.

[ref273] DiCarmine P. M.; Schon T. B.; McCormick T. M.; Klein P. P.; Seferos D. S. Donor–Acceptor Polymers for Electrochemical Supercapacitors: Synthesis, Testing, and Theory. J. Phys. Chem. C 2014, 118, 8295–8307. 10.1021/jp5016214.

[ref274] Ramasamy C.; Palma J.; Anderson M. A 3-V Electrochemical Capacitor Study Based on a Magnesium Polymer Gel Electrolyte by Three Different Carbon Materials. J. Solid State Electrochem 2014, 18, 2903–2911. 10.1007/s10008-014-2557-1.

[ref275] Costa C. M.; Lee Y. H.; Kim J. H.; Lee S. Y.; Lanceros-Méndez S. Recent advances on separator membranes for lithium-ion battery applications: From porous membranes to solid electrolytes. Energy Storage Materials 2019, 22, 346–375. 10.1016/j.ensm.2019.07.024.

[ref276] Saal A.; Hagemann T.; Schubert U. S. Polymers for Battery Applications—Active Materials, Membranes, and Binders. Adv. Energy Mater. 2021, 11, 2001984 10.1002/aenm.202001984.

[ref277] Lee H.; Yanilmaz M.; Toprakci O.; Fu K.; Zhang X. A review of recent developments in membrane separators for rechargeable lithium-ion batteries. Energy Environ. Sci. 2014, 7, 3857–3886. 10.1039/C4EE01432D.

[ref278] Stepniak I.; Galinski M.; Nowacki K.; Wysokowski M.; Jakubowska P.; Bazhenov V. V.; Leisegang T.; Ehrlich H.; Jesionowski T. A novel chitosan/sponge chitin origin material as a membrane for supercapacitors-preparation and characterization. RSC Adv. 2016, 6, 4007–4013. 10.1039/C5RA22047E.

[ref279] Sahoo S.; Ratha S.; Rout C. S.; Nayak S. K. Self-charging supercapacitors for smart electronic devices: a concise review on the recent trends and future sustainability. J. Mater. Sci. 2022, 57, 4399–4440. 10.1007/s10853-022-06875-9.

[ref280] K N.; Rout C. S. Photo-powered integrated supercapacitors: a review on recent developments, challenges and future perspectives. Journal of Materials Chemistry A 2021, 9, 8248–8278. 10.1039/D1TA00444A.

[ref281] Sun Y.; Yan X. Recent Advances in Dual-Functional Devices Integrating Solar Cells and Supercapacitors. Solar RRL 2017, 1, 1700002 10.1002/solr.201700002.

[ref282] Berestok T.; Diestel C.; Ortlieb N.; Buettner J.; Matthews J.; Schulze P. S. C.; Goldschmidt J. C.; Glunz S. W.; Fischer A. High-Efficiency Monolithic Photosupercapacitors: Smart Integration of a Perovskite Solar Cell with a Mesoporous Carbon Double-Layer Capacitor. Solar RRL 2021, 5, 2100662 10.1002/solr.202100662.

[ref283] Todorov T.; Gunawan O.; Guha S. A road towards 25 Perovskite tandem solar cells. Molecular Systems Design and Engineering 2016, 1, 370–376. 10.1039/C6ME00041J.

[ref284] Ouyang Z.; Lou S. N.; Lau D.; Chen J.; Lim S.; Hsiao P.; Wang D.; Amal R.; Ng Y. H.; Lennon A. Monolithic Integration of Anodic Molybdenum Oxide Pseudocapacitive Electrodes on Screen-Printed Silicon Solar Cells for Hybrid Energy Harvesting-Storage Systems. Adv. Energy Mater. 2017, 7, 1602325 10.1002/aenm.201602325.

[ref285] Sun Y.; Yan X. Recent Advances in Dual-Functional Devices Integrating Solar Cells and Supercapacitors. Solar RRL 2017, 1, 1700002 10.1002/solr.201700002.

[ref286] Devadiga D.; Selvakumar M.; Shetty P.; Santosh M. S. Recent progress in dye sensitized solar cell materials and photo-supercapacitors: A review. J. Power Sources 2021, 493, 229698 10.1016/j.jpowsour.2021.229698.

[ref287] Maddala G.; Ambapuram M.; Tankasala V.; Mitty R. Optimal Dye Sensitized Solar Cell and Photocapacitor Performance with Efficient Electrocatalytic SWCNH Assisted Carbon Electrode. ACS Applied Energy Materials 2021, 4, 11225–11233. 10.1021/acsaem.1c02087.

[ref288] Shi C.; Dong H.; Zhu R.; Li H.; Sun Y.; Xu D.; Zhao Q.; Yu D. An ”all-in-one” mesh-typed integrated energy unit for both photoelectric conversion and energy storage in uniform electrochemical system. Nano Energy 2015, 13, 670–678. 10.1016/j.nanoen.2015.03.032.

[ref289] Liu Z.; Zhong Y.; Sun B.; Liu X.; Han J.; Shi T.; Tang Z.; Liao G. Novel Integration of Perovskite Solar Cell and Supercapacitor Based on Carbon Electrode for Hybridizing Energy Conversion and Storage. ACS Applied Materials Interfaces 2017, 9, 22361–22368. 10.1021/acsami.7b01471. 28614655

[ref290] Skunik-Nuckowska M.; Grzejszczyk K.; Kulesza P. J.; Yang L.; Vlachopoulos N.; Häggman L.; Johansson E.; Hagfeldt A. Integration of solid-state dye-sensitized solar cell with metal oxide charge storage material into photoelectrochemical capacitor. J. Power Sources 2013, 234, 91–99. 10.1016/j.jpowsour.2013.01.101.

[ref291] Hsu C. Y.; Chen H. W.; Lee K. M.; Hu C. W.; Ho K. C. A dye-sensitized photo-supercapacitor based on PProDOT-Et2 thick films. J. Power Sources 2010, 195, 6232–6238. 10.1016/j.jpowsour.2009.12.099.

[ref292] Lau S. C.; Lim H. N.; Ravoof T. B.; Yaacob M. H.; Grant D. M.; MacKenzie R. C.; Harrison I.; Huang N. M. A three-electrode integrated photo-supercapacitor utilizing graphene-based intermediate bifunctional electrode. Electrochim. Acta 2017, 238, 178–184. 10.1016/j.electacta.2017.04.003.

[ref293] Narayanan R.; Kumar P. N.; Deepa M.; Srivastava A. K. Combining Energy Conversion and Storage: A Solar Powered Supercapacitor. Electrochim. Acta 2015, 178, 113–126. 10.1016/j.electacta.2015.07.121.

[ref294] Das A.; Deshagani S.; Kumar R.; Deepa M. Bifunctional Photo-Supercapacitor with a New Architecture Converts and Stores Solar Energy as Charge. ACS Applied Materials Interfaces 2018, 10, 35932–35945. 10.1021/acsami.8b11399. 30251828

[ref295] Zheng R.; Li H.; Hu Z.; Wang L.; Lü W.; Li F. Photo-supercapacitor based on quantum dot-sensitized solar cells and active carbon supercapacitors. Journal of Materials Science: Materials in Electronics 2022, 33, 22309–22318. 10.1007/s10854-022-09010-1.

[ref296] Yang Z.; Li L.; Luo Y.; He R.; Qiu L.; Lin H.; Peng H. An integrated device for both photoelectric conversion and energy storage based on free-standing and aligned carbon nanotube film. J. Mater. Chem. A 2013, 1, 954–958. 10.1039/C2TA00113F.

[ref297] Kim J.; Lee S. M.; Hwang Y.-H.; Lee S.; Park B.; Jang J.-H.; Lee K. A highly efficient self-power pack system integrating supercapacitors and photovoltaics with an area-saving monolithic architecture. Journal of Materials Chemistry A 2017, 5, 1906–1912. 10.1039/C6TA09117B.

[ref298] Xu J.; Ku Z.; Zhang Y.; Chao D.; Fan H. J. Integrated Photo-Supercapacitor Based on PEDOT Modified Printable Perovskite Solar Cell. Advanced Materials Technologies 2016, 1, 1600074 10.1002/admt.201600074.

[ref299] Wee G.; Salim T.; Lam Y. M.; Mhaisalkar S. G.; Srinivasan M. Printable photo-supercapacitor using single-walled carbon nanotubes. Energy Environ. Sci. 2011, 4, 413–416. 10.1039/C0EE00296H.

[ref300] Lechêne B. P.; Cowell M.; Pierre A.; Evans J. W.; Wright P. K.; Arias A. C. Organic solar cells and fully printed super-capacitors optimized for indoor light energy harvesting. Nano Energy 2016, 26, 631–640. 10.1016/j.nanoen.2016.06.017.

[ref301] Jin Y.; Sun L.; Qin L.; Liu Y.; Li Z.; Zhou Y.; Zhang F. Solution-processed solar-charging power units made of organic photovoltaic modules and asymmetric super-capacitors. Appl. Phys. Lett. 2021, 118, 203902 10.1063/5.0044652.

[ref302] Xu X.; Li S.; Zhang H.; Shen Y.; Zakeeruddin S. M.; Graetzel M.; Cheng Y.-B.; Wang M. A Power Pack Based on Organometallic Perovskite Solar Cell and Supercapacitor. ACS Nano 2015, 9, 1782–1787. 10.1021/nn506651m. 25611128

[ref303] Scalia A.; Varzi A.; Lamberti A.; Jacob T.; Passerini S. Portable High Voltage Integrated Harvesting-Storage Device Employing Dye-Sensitized Solar Module and All-Solid-State Electrochemical Double Layer Capacitor. Front. Chem. 2018, 6, 1–8. 10.3389/fchem.2018.00443. 30320074 PMC6167942

[ref304] Chien C. T.; Hiralal P.; Wang D. Y.; Huang I. S.; Chen C. C.; Chen C. W.; Amaratunga G. A. Graphene-Based Integrated Photovoltaic Energy Harvesting/Storage Device. Small 2015, 11, 2929–2937. 10.1002/smll.201403383. 25703342

[ref305] Dong P.; Rodrigues M. T. F.; Zhang J.; Borges R. S.; Kalaga K.; Reddy A. L.; Silva G. G.; Ajayan P. M.; Lou J. A flexible solar cell/supercapacitor integrated energy device. Nano Energy 2017, 42, 181–186. 10.1016/j.nanoen.2017.10.035.

[ref306] Westover A. S.; Share K.; Carter R.; Cohn A. P.; Oakes L.; Pint C. L. Direct Integration of a Supercapacitor into the Backside of a Silicon Photovoltaic Device. Appl. Phys. Lett. 2014, 104, 213905 10.1063/1.4880211.

[ref307] Thekkekara L. V.; Jia B.; Zhang Y.; Qiu L.; Li D.; Gu M. On-Chip Energy Storage Integrated with Solar Cells Using a Laser Scribed Graphene Oxide Film. Appl. Phys. Lett. 2015, 107, 031105 10.1063/1.4927145.

[ref308] Liu R.; Wang J.; Sun T.; Wang M.; Wu C.; Zou H.; Song T.; Zhang X.; Lee S.-T.; Wang Z. L.; Sun B. Silicon Nanowire/Polymer Hybrid Solar Cell-Supercapacitor: A Self-Charging Power Unit with a Total Efficiency of 10.5. Nano Lett. 2017, 17, 4240–4247. 10.1021/acs.nanolett.7b01154. 28586231

[ref309] Liu H.; Li M.; Kaner R. B.; Chen S.; Pei Q. Monolithically Integrated Self-Charging Power Pack Consisting of a Silicon Nanowire Array/Conductive Polymer Hybrid Solar Cell and a Laser-Scribed Graphene Supercapacitor. ACS Appl. Mater. Interfaces 2018, 10, 15609–15615. 10.1021/acsami.8b00014. 29692171

[ref310] Yilmaz M.; Hsu S. H.; Raina S.; Howell M.; Kang W. P. Integrated photocapacitors based on dye-sensitized TiO 2 /FTO as photoanode and MnO2 coated micro-array CNTs as supercapacitor counter electrode with TEABF4 electrolyte. J. Renewable Sustainable Energy 2018, 10, 063503 10.1063/1.5050038.

[ref311] Liang J.; Zhu G.; Wang C.; Wang Y.; Zhu H.; Hu Y.; Lv H.; Chen R.; Ma L.; Chen T.; Jin Z.; Liu J. MoS 2 -Based All-Purpose Fibrous Electrode and Self-Powering Energy Fiber for Efficient Energy Harvesting and Storage. Adv. Energy Mater. 2017, 7, 1601208 10.1002/aenm.201601208.

[ref312] Chen H.-W.; Hsu C.-Y.; Chen J.-G.; Lee K.-M.; Wang C.-C.; Huang K.-C.; Ho K.-C. Plastic Dye-Sensitized Photo-Supercapacitor Using Electrophoretic Deposition and Compression Methods. J. Power Sources 2010, 195, 6225–6231. 10.1016/j.jpowsour.2010.01.009.

[ref313] Xu J.; Wu H.; Lu L.; Leung S.-F.; Chen D.; Chen X.; Fan Z.; Shen G.; Li D. Integrated Photo-supercapacitor Based on Bi-polar TiO2 Nanotube Arrays with Selective One-Side Plasma-Assisted Hydrogenation. Adv. Funct. Mater. 2014, 24, 1840–1846. 10.1002/adfm.201303042.

[ref314] Dong P.; Rodrigues M.-T. F.; Zhang J.; Borges R. S.; Kalaga K.; Reddy A. L. M.; Silva G. G.; Ajayan P. M.; Lou J. A Flexible Solar Cell/Supercapacitor Integrated Energy Device. Nano Energy 2017, 42, 181–186. 10.1016/j.nanoen.2017.10.035.

[ref315] Kulesza P. J.; Skunik-Nuckowska M.; Grzejszczyk K.; Vlachopoulos N.; Yang L.; Häggman L.; Hagfeldt A. Development of Solid-State Photo-Supercapacitor by Coupling Dye-Sensitized Solar Cell Utilizing Conducting Polymer Charge Relay with Proton-Conducting Membrane Based Electrochemical Capacitor. ECS Trans 2013, 50, 235 10.1149/05043.0235ecst.

[ref316] Lau S. C.; Lim H. N.; Ravoof T. B. S. A.; Yaacob M. H.; Grant D. M.; MacKenzie R. C. I.; Harrison I.; Huang N. M. A Three-Electrode Integrated Photo-Supercapacitor Utilizing Graphene-Based Intermediate Bifunctional Electrode. Electrochim. Acta 2017, 238, 178–184. 10.1016/j.electacta.2017.04.003.

[ref317] Scalia A.; Varzi A.; Lamberti A.; Jacob T.; Passerini S. Portable High Voltage Integrated Harvesting-Storage Device Employing Dye-Sensitized Solar Module and All-Solid-State Electrochemical Double Layer Capacitor. Front. Chem. 2018, 6. 10.3389/fchem.2018.00443 PMC6167942 30320074

[ref318] Scalia A.; Varzi A.; Lamberti A.; Tresso E.; Jeong S.; Jacob T.; Passerini S. High Energy and High Voltage Integrated Photo-Electrochemical Double Layer Capacitor. Sustainable Energy Fuels 2018, 2, 968–977. 10.1039/C8SE00003D.

[ref319] Ojha M.; Wu B.; Deepa M. NiCo Metal-Organic Framework and Porous Carbon Interlayer-Based Supercapacitors Integrated with a Solar Cell for a Stand-Alone Power Supply System. ACS Appl. Mater. Interfaces 2020, 12, 42749–42762. 10.1021/acsami.0c10883. 32840351

[ref320] Solís-Cortés D.; Navarrete-Astorga E.; Schrebler R.; Peinado-Pérez J. J.; Martín F.; Ramos-Barrado J. R.; Dalchiele E. A. A Solid-State Integrated Photo-Supercapacitor Based on ZnO Nanorod Arrays Decorated with Ag2S Quantum Dots as the Photoanode and a PEDOT Charge Storage Counter-Electrode. RSC Adv. 2020, 10, 5712–5721. 10.1039/C9RA10635A. 35497434 PMC9049565

[ref321] Zhang Z.; Chen X.; Chen P.; Guan G.; Qiu L.; Lin H.; Yang Z.; Bai W.; Luo Y.; Peng H. Integrated Polymer Solar Cell and Electrochemical Supercapacitor in a Flexible and Stable Fiber Format. Adv. Mater. 2014, 26, 466–470. 10.1002/adma.201302951. 24174379

[ref322] Tuukkanen S.; Välimäki M.; Lehtimäki S.; Vuorinen T.; Lupo D. Behaviour of one-step spray-coated carbon nanotube supercapacitor in ambient light harvester circuit with printed organic solar cell and electrochromic display. Sci. Rep. 2016, 6, 22967 10.1038/srep22967. 26957019 PMC4783710

[ref323] Liu R.; Takakuwa M.; Li A.; Inoue D.; Hashizume D.; Yu K.; Umezu S.; Fukuda K.; Someya T. An Efficient Ultra-Flexible Photo-Charging System Integrating Organic Photovoltaics and Supercapacitors. Adv. Energy Mater. 2020, 10, 2000523 10.1002/aenm.202000523.

[ref324] Qin L.; Jiang J.; Tao Q.; Wang C.; Persson I.; Fahlman M.; Persson P. O. Å.; Hou L.; Rosen J.; Zhang F. A Flexible Semitransparent Photovoltaic Supercapacitor Based on Water-Processed MXene Electrodes. J. Mater. Chem. A 2020, 8, 5467–5475. 10.1039/D0TA00687D.

[ref325] Chien C.-T.; Hiralal P.; Wang D.-Y.; Huang I.-S.; Chen C.-C.; Chen C.-W.; Amaratunga G. A. J. Graphene-Based Integrated Photovoltaic Energy Harvesting/Storage Device. Small 2015, 11, 2929–2937. 10.1002/smll.201403383. 25703342

[ref326] Jin Y.; Sun L.; Qin L.; Liu Y.; Li Z.; Zhou Y.; Zhang F. Solution-Processed Solar-Charging Power Units Made of Organic Photovoltaic Modules and Asymmetric Super-Capacitors. Appl. Phys. Lett. 2021, 118, 203902 10.1063/5.0044652.

[ref327] Sun J.; Gao K.; Lin X.; Gao C.; Ti D.; Zhang Z. Laser-Assisted Fabrication of Microphotocapacitors with High Energy Density and Output Voltage. ACS Appl. Mater. Interfaces 2021, 13, 419–428. 10.1021/acsami.0c16677. 33386055

[ref328] Liu R.; Liu C.; Fan S. A photocapacitor based on organometal halide perovskite and PANI/CNT composites integrated using a CNT bridge. Journal of Materials Chemistry A 2017, 5, 23078–23084. 10.1039/C7TA06297D.

[ref329] Liang J.; Zhu G.; Lu Z.; Zhao P.; Wang C.; Ma Y.; Xu Z.; Wang Y.; Hu Y.; Ma L.; Chen T.; Tie Z.; Liu J.; Jin Z. Integrated perovskite solar capacitors with high energy conversion efficiency and fast photo-charging rate. Journal of Materials Chemistry A 2018, 6, 2047–2052. 10.1039/C7TA09099D.

[ref330] Yang Y.; Fan L.; Pham N. D.; Yao D.; Wang T.; Wang Z.; Wang H. Self-charging flexible solar capacitors based on integrated perovskite solar cells and quasi-solid-state supercapacitors fabricated at low temperature. J. Power Sources 2020, 479, 229046 10.1016/j.jpowsour.2020.229046.

[ref331] Zhang F.; Li W.; Xu Z.; Ye M.; Xu H.; Guo W.; Liu X. Highly flexible and scalable photo-rechargeable power unit based on symmetrical nanotube arrays. Nano Energy 2018, 46, 168–175. 10.1016/j.nanoen.2018.01.041.

[ref332] Xu J.; Ku Z.; Zhang Y.; Chao D.; Fan H. J. Integrated Photo-Supercapacitor Based on PEDOT Modified Printable Perovskite Solar Cell. Adv. Mater. Technol. 2016, 1, 1600074 10.1002/admt.201600074.

[ref333] Zhou F.; Ren Z.; Zhao Y.; Shen X.; Wang A.; Li Y. Y.; Surya C.; Chai Y. Perovskite Photovoltachromic Supercapacitor with All-Transparent Electrodes. ACS Nano 2016, 10, 5900–5908. 10.1021/acsnano.6b01202. 27159013

[ref334] Liu Z.; Zhong Y.; Sun B.; Liu X.; Han J.; Shi T.; Tang Z.; Liao G. Novel Integration of Perovskite Solar Cell and Supercapacitor Based on Carbon Electrode for Hybridizing Energy Conversion and Storage. ACS Appl. Mater. Interfaces 2017, 9, 22361–22368. 10.1021/acsami.7b01471. 28614655

[ref335] Du P.; Hu X.; Yi C.; Liu H. C.; Liu P.; Zhang H.-L.; Gong X. Self-Powered Electronics by Integration of Flexible Solid-State Graphene-Based Supercapacitors with High Performance Perovskite Hybrid Solar Cells. Adv. Funct. Mater. 2015, 25, 2420–2427. 10.1002/adfm.201500335.

[ref336] Liang J.; Zhu G.; Wang C.; Zhao P.; Wang Y.; Hu Y.; Ma L.; Tie Z.; Liu J.; Jin Z. An All-Inorganic Perovskite Solar Capacitor for Efficient and Stable Spontaneous Photocharging. Nano Energy 2018, 52, 239–245. 10.1016/j.nanoen.2018.07.060.

[ref337] Pazoki M.; Cappel U. B.; Johansson E. M. J.; Hagfeldt A.; Boschloo G. Characterization techniques for dye-sensitized solar cells. Energy Environmental Science 2017, 10, 672–709. 10.1039/C6EE02732F.

[ref338] Kar K. K. Springer series in materials science 300 handbook of nanocomposite supercapacitor materials I; Springer, 2020; p 378.

[ref339] Song Z.; Wu J.; Sun L.; Zhu T.; Deng C.; Wang X.; Li G.; Du Y.; Chen Q.; Sun W.; Fan L.; Chen H.; Lin J.; Lan Z. Nano Energy Photocapacitor integrating perovskite solar cell and symmetrical supercapacitor generating a conversion storage efficiency over 20. Nano Energy 2022, 100, 107501 10.1016/j.nanoen.2022.107501.

[ref340] Ehrler B.; Alarcón-Lladó E.; Tabernig S. W.; Veeken T.; Garnett E. C.; Polman A. Photovoltaics reaching for the shockley-queisser limit. ACS Energy Letters 2020, 5, 3029–3033. 10.1021/acsenergylett.0c01790.

[ref341] Lechene B. P.; Clerc R.; Arias A. C. Theoretical analysis and characterization of the energy conversion and storage efficiency of photo-supercapacitors. Sol. Energy Mater. Sol. Cells 2017, 172, 202–212. 10.1016/j.solmat.2017.07.034.

[ref342] Wang L.; Wen L.; Tong Y.; Wang S.; Hou X.; An X.; Dou S. X.; Liang J. Photo-rechargeable batteries and supercapacitors: Critical roles of carbon-based functional materials. Carbon Energy 2021, 3, 225–252. 10.1002/cey2.105.

[ref343] Huang S.; Zhu X.; Sarkar S.; Zhao Y. Challenges and opportunities for supercapacitors. APL Mater. 2019, 7, 100901 10.1063/1.5116146.

[ref344] Fedorov M. V.; Kornyshev A. A. Ionic liquids at electrified interfaces. Chem. Rev. 2014, 114, 2978–3036. 10.1021/cr400374x. 24588221

[ref345] Yang Y.; Hoang M. T.; Bhardwaj A.; Wilhelm M.; Mathur S.; Wang H. Perovskite solar cells based self-charging power packs: Fundamentals, applications and challenges. Nano Energy 2022, 94, 106910 10.1016/j.nanoen.2021.106910.

[ref346] Jeanmairet G.; Rotenberg B.; Salanne M. Microscopic Simulations of Electrochemical Double-Layer Capacitors. Chem. Rev. 2022, 122, 10860–10898. 10.1021/acs.chemrev.1c00925. 35389636 PMC9227719

[ref347] Zhang S.; Pan N. Supercapacitors performance evaluation. Adv. Energy Mater. 2015, 5, 1401401 10.1002/aenm.201401401.

[ref348] Raga S. R.; Barea E. M.; Fabregat-Santiago F. Analysis of the origin of open circuit voltage in dye solar cells. J. Phys. Chem. Lett. 2012, 3, 1629–1634. 10.1021/jz3005464. 26285719

[ref349] Yum J.-H.; Baranoff E.; Kessler F.; Moehl T.; Ahmad S.; Bessho T.; Marchioro A.; Ghadiri E.; Moser J.-E.; Yi C.; Nazeeruddin M. K.; Grätzel M. A cobalt complex redox shuttle for dye-sensitized solar cells with high open-circuit potentials. Nat. Commun. 2012, 3, 631 10.1038/ncomms1655. 22252555 PMC3272578

[ref350] Snaith H. J. Estimating the maximum attainable efficiency in Dye-sensitized solar cells. Adv. Funct. Mater. 2010, 20, 13–19. 10.1002/adfm.200901476.

[ref351] Marinado T.; Nonomura K.; Nissfolk J.; Karlsson M. K.; Hagberg D. P.; Sun L.; Mori S.; Hagfeldt A. How the nature of triphenylamine-polyene dyes in dye-sensitized solar cells affects the open-circuit voltage and electron lifetimes. Langmuir 2010, 26, 2592–2598. 10.1021/la902897z. 19863060

[ref352] Zhang W.; Wu Y.; Bahng H. W.; Cao Y.; Yi C.; Saygili Y.; Luo J.; Liu Y.; Kavan L.; Moser J.-E.; Hagfeldt A.; Tian H.; Zakeeruddin S. M.; Zhu W.-H.; Grätzel M. Comprehensive control of voltage loss enables 11.7. Environmental Science 2018, 11, 1779–1787. 10.1039/C8EE00661J.

[ref353] Elumalai N. K.; Uddin A. Open circuit voltage of organic solar cells: An in-depth review. Energy Environ. Sci. 2016, 9, 391–410. 10.1039/C5EE02871J.

[ref354] Yao J.; Kirchartz T.; Vezie M. S.; Faist M. A.; Gong W.; He Z.; Wu H.; Troughton J.; Watson T.; Bryant D.; Nelson J. Quantifying losses in open-circuit voltage in solution-processable solar cells. Phys. Rev. Appl. 2015, 4, 1–10. 10.1103/PhysRevApplied.4.014020.

[ref355] Widmer J.; Tietze M.; Leo K.; Riede M. Open-circuit voltage and effective gap of organic solar cells. Adv. Funct. Mater. 2013, 23, 5814–5821. 10.1002/adfm.201301048.

[ref356] Vandewal K.; Tvingstedt K.; Gadisa A.; Inganäs O.; Manca J. V. On the origin of the open-circuit voltage of polymer-fullerene solar cells. Nat. Mater. 2009, 8, 904–909. 10.1038/nmat2548. 19820700

[ref357] Azzouzi M.; Kirchartz T.; Nelson J. Factors Controlling Open-Circuit Voltage Losses in Organic Solar Cells. Trends in Chemistry 2019, 1, 49–62. 10.1016/j.trechm.2019.01.010.

[ref358] Credgington D.; Durrant J. R. Insights from transient optoelectronic analyses on the open-circuit voltage of organic solar cells. J. Phys. Chem. Lett. 2012, 3, 1465–1478. 10.1021/jz300293q. 26285623

[ref359] Guo Z.; Jena A. K.; Kim G. M.; Miyasaka T. The high open-circuit voltage of perovskite solar cells: a review. Energy Environ. Sci. 2022, 15, 3171–3222. 10.1039/D2EE00663D.

[ref360] Krückemeier L.; Rau U.; Stolterfoht M.; Kirchartz T. How to Report Record Open-Circuit Voltages in Lead-Halide Perovskite Solar Cells. Adv. Energy Mater. 2020, 10, 1902573 10.1002/aenm.201902573.

[ref361] Shao Y.; Yuan Y.; Huang J. Correlation of energy disorder and open-circuit voltage in hybrid perovskite solar cells. Nature Energy 2016, 1, 1–6. 10.1038/nenergy.2015.1.

[ref362] Wheeler S.; Bryant D.; Troughton J.; Kirchartz T.; Watson T.; Nelson J.; Durrant J. R. Transient Optoelectronic Analysis of the Impact of Material Energetics and Recombination Kinetics on the Open-Circuit Voltage of Hybrid Perovskite Solar Cells. J. Phys. Chem. C 2017, 121, 13496–13506. 10.1021/acs.jpcc.7b02411.

[ref363] Tress W.; Yavari M.; Domanski K.; Yadav P.; Niesen B.; Correa Baena J. P.; Hagfeldt A.; Graetzel M. Interpretation and evolution of open-circuit voltage, recombination, ideality factor and subgap defect states during reversible light-soaking and irreversible degradation of perovskite solar cells. Energy Environ. Sci. 2018, 11, 151–165. 10.1039/C7EE02415K.

[ref364] Liang J.; Wang D. W. Design Rationale and Device Configuration of Lithium-Ion Capacitors. Adv. Energy Mater. 2022, 12, 2200920 10.1002/aenm.202200920.

[ref365] Ding J.; Hu W.; Paek E.; Mitlin D. Review of Hybrid Ion Capacitors: From Aqueous to Lithium to Sodium. Chem. Rev. 2018, 118, 6457–6498. 10.1021/acs.chemrev.8b00116. 29953230

[ref366] Dubal D. P.; Ayyad O.; Ruiz V.; Gómez-Romero P. Hybrid energy storage: the merging of battery and supercapacitor chemistries. Chem. Soc. Rev. 2015, 44, 1777–1790. 10.1039/C4CS00266K. 25623995

[ref367] Vlad A.; Singh N.; Rolland J.; Melinte S.; Ajayan P. M.; Gohy J. F. Hybrid supercapacitor-battery materials for fast electrochemical charge storage. Sci. Rep. 2014, 4, 1–7. 10.1038/srep04315. PMC3945924 24603843

[ref368] Jiang J. M.; Li Z. W.; Zhang Z. T.; Wang S. J.; Xu H.; Zheng X. R.; Chen Y. X.; Ju Z. C.; Dou H.; Zhang X. G. Recent advances and perspectives on prelithiation strategies for lithium-ion capacitors. Rare Metals 2022, 41, 3322–3335. 10.1007/s12598-022-02050-w.

[ref369] Xu Z.; Li M.; Sun W.; Tang T.; Lu J.; Wang X. An Ultrafast, Durable, and High-Loading Polymer Anode for Aqueous Zinc-Ion Batteries and Supercapacitors. Adv. Mater. 2022, 34, 2200077 10.1002/adma.202200077. 35355338

[ref370] Chen G. Z. Supercapacitor and supercapattery as emerging electrochemical energy stores. International Materials Reviews 2017, 62, 173–202. 10.1080/09506608.2016.1240914.

[ref371] Zhang S.; Li C.; Zhang X.; Sun X.; Wang K.; Ma Y. High Performance Lithium-Ion Hybrid Capacitors Employing Fe 3 O 4 – Graphene Composite Anode and Activated Carbon Cathode. ACS Applied Materials Interfaces 2017, 9, 17136–17144. 10.1021/acsami.7b03452. 28474525

[ref372] Amatucci G. G.; Badway F.; Du Pasquier A.; Zheng T. An Asymmetric Hybrid Nonaqueous Energy Storage Cell. J. Electrochem. Soc. 2001, 148, A930 10.1149/1.1383553.

[ref373] Liang J.; Wang D. W. Design Rationale and Device Configuration of Lithium-Ion Capacitors. Adv. Energy Mater. 2022, 12, 2200920 10.1002/aenm.202200920.

[ref374] IEA. Energy Harvesting Technologies for Buildings; IEA, 2020.

[ref375] Hittinger E.; Jaramillo P. Internet of things: Energy boon or bane?. Science 2019, 364, 326–328. 10.1126/science.aau8825. 31023909

[ref376] Krishnamoorthy R.; Soubache I. D.; Jain S. Wireless Communication Based Evaluation of Power Consumption for Constrained Energy System. Wireless Personal Commun. 2022, 127, 737 10.1007/s11277-021-08402-6.

[ref377] Gupta N.; Vaisla K. S.; Kumar R. Design of a Structured Hypercube Network Chip Topology Model for Energy Efficiency in Wireless Sensor Network Using Machine Learning. SN Comput. Sci. 2021, 2, 1–13. 10.1007/s42979-021-00766-7.

[ref378] Wang C.; Gu J.; Sanjuán Martínez O.; González Crespo R. Economic and environmental impacts of energy efficiency over smart cities and regulatory measures using a smart technological solution. Sustainable Energy Technologies Assessments 2021, 47, 101422 10.1016/j.seta.2021.101422.

[ref379] Bhushan S.; Kumar M.; Kumar P.; Stephan T.; Shankar A.; Liu P. FAJIT: a fuzzy-based data aggregation technique for energy efficiency in wireless sensor network. Complex Intelligent Systems 2021, 7, 997–1007. 10.1007/s40747-020-00258-w.

[ref380] Aldegheishem A.; Anwar M.; Javaid N.; Alrajeh N.; Shafiq M.; Ahmed H. Towards Sustainable Energy Efficiency with Intelligent Electricity Theft Detection in Smart Grids Emphasising Enhanced Neural Networks. IEEE Access 2021, 9, 25036–25061. 10.1109/ACCESS.2021.3056566.

[ref381] Brebels J.; Klider K. C.; Kelchtermans M.; Verstappen P.; Van Landeghem M.; Van Doorslaer S.; Goovaerts E.; Garcia J. R.; Manca J.; Lutsen L.; Vanderzande D.; Maes W. Low bandgap polymers based on bay-annulated indigo for organic photovoltaics: Enhanced sustainability in material design and solar cell fabrication. Org. Electron. 2017, 50, 264–272. 10.1016/j.orgel.2017.07.037.

[ref382] Grifoni F.; Bonomo M.; Naim W.; Barbero N.; Alnasser T.; Dzeba I.; Giordano M.; Tsaturyan A.; Urbani M.; Torres T.; Barolo C.; Sauvage F. Toward Sustainable, Colorless, and Transparent Photovoltaics: State of the Art and Perspectives for the Development of Selective Near-Infrared Dye-Sensitized Solar Cells. Adv. Energy Mater. 2021, 11, 2101598 10.1002/aenm.202101598.

[ref383] Grisorio R.; De Marco L.; Baldisserri C.; Martina F.; Serantoni M.; Gigli G.; Suranna G. P. Sustainability of organic dye-sensitized solar cells: The role of chemical synthesis. ACS Sustainable Chem. Eng. 2015, 3, 770–777. 10.1021/acssuschemeng.5b00108.

[ref384] Charles R. G.; Davies M. L.; Douglas P. Third generation photovoltaics — Early intervention for circular economy and a sustainable future. 2016 Electronics Goes Green 2016+ (EGG); IEEE: New York, 2016; pp 1–8.

[ref385] Parisi M. L.; Maranghi S.; Vesce L.; Sinicropi A.; Di Carlo A.; Basosi R. Prospective life cycle assessment of third-generation photovoltaics at the pre-industrial scale: A long-term scenario approach. Renewable and Sustainable Energy Reviews 2020, 121, 109703 10.1016/j.rser.2020.109703.

[ref386] Vohra V. Can Polymer Solar Cells Open the Path to Sustainable and Efficient Photovoltaic Windows Fabrication?. Chem. Rec. 2019, 19, 1166–1178. 10.1002/tcr.201800072. 30251409

[ref387] Victoria M.; Haegel N.; Peters I. M.; Sinton R.; Jäger-Waldau A.; del Cañizo C.; Breyer C.; Stocks M.; Blakers A.; Kaizuka I.; Komoto K.; Smets A. Solar photovoltaics is ready to power a sustainable future. Joule 2021, 5, 1041–1056. 10.1016/j.joule.2021.03.005.

[ref388] Krebs-Moberg M.; Pitz M.; Dorsette T. L.; Gheewala S. H. Third generation of photovoltaic panels: A life cycle assessment. Renewable Energy 2021, 164, 556–565. 10.1016/j.renene.2020.09.054.

[ref389] Choudhary P.; Srivastava R. K. Sustainability perspectives- a review for solar photovoltaic trends and growth opportunities. Journal of Cleaner Production 2019, 227, 589–612. 10.1016/j.jclepro.2019.04.107.

[ref390] Gressler S.; Part F.; Scherhaufer S.; Obersteiner G.; Huber-Humer M. Advanced materials for emerging photovoltaic systems – Environmental hotspots in the production and end-of-life phase of organic, dye-sensitized, perovskite, and quantum dots solar cells. Sustainable Materials and Technologies 2022, 34, e00501 10.1016/j.susmat.2022.e00501.

[ref391] Mariotti N.; Bonomo M.; Fagiolari L.; Barbero N.; Gerbaldi C.; Bella F.; Barolo C. Recent advances in eco-friendly and cost-effective materials towards sustainable dye-sensitized solar cells. Green Chem. 2020, 22, 7168–7218. 10.1039/D0GC01148G.

[ref392] Yu P.; Zhang Z.; Zheng L.; Teng F.; Hu L.; Fang X. A Novel Sustainable Flour Derived Hierarchical Nitrogen-Doped Porous Carbon/Polyaniline Electrode for Advanced Asymmetric Supercapacitors. Adv. Energy Mater. 2016, 6, 1601111 10.1002/aenm.201601111.

[ref393] Ling Z.; Wang Z.; Zhang M.; Yu C.; Wang G.; Dong Y.; Liu S.; Wang Y.; Qiu J. Sustainable Synthesis and Assembly of Biomass-Derived B/N Co-Doped Carbon Nanosheets with Ultrahigh Aspect Ratio for High-Performance Supercapacitors. Adv. Funct. Mater. 2016, 26, 111–119. 10.1002/adfm.201504004.

[ref394] Wang Z.; Tammela P.; Strømme M.; Nyholm L. Cellulose-based Supercapacitors: Material and Performance Considerations. Adv. Energy Mater. 2017, 7, 1700130 10.1002/aenm.201700130.

[ref395] Xie Q.; Bao R.; Zheng A.; Zhang Y.; Wu S.; Xie C.; Zhao P. Sustainable Low-Cost Green Electrodes with High Volumetric Capacitance for Aqueous Symmetric Supercapacitors with High Energy Density. ACS Sustainable Chem. Eng. 2016, 4, 1422–1430. 10.1021/acssuschemeng.5b01417.

[ref396] Vijayakumar M.; Bharathi Sankar A.; Sri Rohita D.; Rao T. N.; Karthik M. Conversion of Biomass Waste into High Performance Supercapacitor Electrodes for Real-Time Supercapacitor Applications. ACS Sustainable Chem. Eng. 2019, 7, 17175–17185. 10.1021/acssuschemeng.9b03568.

[ref397] Altinci O. C.; Demir M. Beyond Conventional Activating Methods, a Green Approach for the Synthesis of Biocarbon and Its Supercapacitor Electrode Performance. Energy Fuels 2020, 34, 7658–7665. 10.1021/acs.energyfuels.0c01103.

[ref398] Wang C.; Xiong Y.; Wang H.; Sun Q. All-round utilization of biomass derived all-solid-state asymmetric carbon-based supercapacitor. J. Colloid Interface Sci. 2018, 528, 349–359. 10.1016/j.jcis.2018.05.103. 29860203

[ref399] Karaman C.; Karaman O.; Atar N.; Yola M. L. Sustainable electrode material for high-energy supercapacitor: Biomass-derived graphene-like porous carbon with three-dimensional hierarchically ordered ion highways. Phys. Chem. Chem. Phys. 2021, 23, 12807–12821. 10.1039/D1CP01726H. 34059859

[ref400] Poizot P.; Gaubicher J.; Renault S.; Dubois L.; Liang Y.; Yao Y. Opportunities and Challenges for Organic Electrodes in Electrochemical Energy Storage. Chem. Rev. 2020, 120, 6490–6557. 10.1021/acs.chemrev.9b00482. 32207919

